# Post-surgery interventions for hip fracture: a systematic review of randomized controlled trials

**DOI:** 10.1186/s12891-023-06512-9

**Published:** 2023-05-25

**Authors:** Jie Kie Phang, Zhui Ying Lim, Wan Qi Yee, Cheryl Yan Fang Tan, Yu Heng Kwan, Lian Leng Low

**Affiliations:** 1 Centre for Population Health Research and Implementation (CPHRI), SingHealth Regional Health System, SingHealth, Singapore, Singapore; 2 Population Health & Integrated Care Office (PHICO), Singapore General Hospital, Singapore, Singapore; 3 Bright Vision Community Hospital, SingHealth Community Hospitals, Singapore, Singapore; 4 SingHealth Internal Medicine Residency Programme, Singapore, Singapore; 5 Program in Health Systems and Services Research, Duke-NUS Medical School, Singapore, Singapore; 6 Department of Pharmacy, National University of Singapore, Singapore, Singapore; 7 Department of Family Medicine & Continuing Care, Singapore General Hospital, Singapore, Singapore; 8 SingHealth Duke-NUS Family Medicine Academic Clinical Program, Duke-NUS Medical School, Singapore, Singapore; 9 Department of Post-Acute & Continuing Care, SingHealth Community Hospitals, 10 Hospital Boulevard, Singapore, 168852 Singapore

**Keywords:** Hip fracture, Systematic review, Rehabilitation, Post-surgery, Post-operative

## Abstract

**Background:**

Interventions provided after hip fracture surgery have been shown to reduce mortality and improve functional outcomes. While some systematic studies have evaluated the efficacy of post-surgery interventions, there lacks a systematically rigorous examination of all the post-surgery interventions which allows healthcare providers to easily identify post-operative interventions most pertinent to patient’s recovery.

**Objectives:**

We aim to provide an overview of the available evidence on post-surgery interventions provided in the acute, subacute and community settings to improve outcomes for patients with hip fractures.

**Methods:**

We performed a systematic literature review guided by the Preferred Reporting Items for Systematic review and Meta-Analysis (PRISMA). We included articles that were (1) randomized controlled trials (RCTs), (2) involved post-surgery interventions that were conducted in the acute, subacute or community settings and (3) conducted among older patients above 65 years old with any type of non-pathological hip fracture that was surgically treated, and who were able to walk without assistance prior to the fracture. We excluded (1) non–English language articles, (2) abstract-only publications, (3) articles with only surgical interventions, (4) articles with interventions that commenced pre-surgery or immediately upon completion of surgery or blood transfusion, (5) animal studies. Due to the large number of RCTs identified, we only included “good quality” RCTs with Jadad score ≥ 3 for data extraction and synthesis.

**Results:**

Our literature search has identified 109 good quality RCTs on post-surgery interventions for patients with fragility hip fractures. Among the 109 RCTs, 63% of the identified RCTs (n = 69) were related to rehabilitation or medication/nutrition supplementation, with the remaining RCTs focusing on osteoporosis management, optimization of clinical management, prevention of venous thromboembolism, fall prevention, multidisciplinary approaches, discharge support, management of post-operative anemia as well as group learning and motivational interviewing. For the interventions conducted in inpatient and outpatient settings investigating medication/nutrition supplementation, all reported improvement in outcomes (ranging from reduced postoperative complications, reduced length of hospital stay, improved functional recovery, reduced mortality rate, improved bone mineral density and reduced falls), except for a study investigating anabolic steroids. RCTs involving post-discharge osteoporosis care management generally reported improved osteoporosis management except for a RCT investigating multidisciplinary post-fracture clinic led by geriatrician with physiotherapist and occupational therapist. The trials investigating group learning and motivational interviewing also reported positive outcome respectively. The other interventions yielded mixed results. The interventions in this review had minor or no side effects reported.

**Conclusions:**

The identified RCTs regarding post-surgery interventions were heterogeneous in terms of type of interventions, settings and outcome measures. Combining interventions across inpatient and outpatient settings may be able to achieve better outcomes such as improved physical function recovery and improved nutritional status recovery. For example, nutritional supplementation could be made available for patients who have undergone hip fracture surgery in the inpatient settings, followed by post-discharge outpatient osteoporosis care management. The findings from this review can aid in clinical practice by allowing formulation of thematic program with combination of interventions as part of bundled care to improve outcome for patients who have undergone hip fracture surgery.

**Supplementary Information:**

The online version contains supplementary material available at 10.1186/s12891-023-06512-9.

## Introduction

Hip fracture is an important medical condition associated with adverse outcomes, including mortality [1]. The incidence of hip fractures is expected to increase due to ageing populations worldwide - the number of hip fractures occurring in the world each year will rise from 1.66 million in 1990 to 6.26 million by 2050 [2]. Only a minority of individuals fully regained their pre-fracture functional level [3]. Elder patients suffering from proximal femoral fractures were more likely to develop depressive symptoms, that further impeded on functional recovery and increased mortality [4]. With higher incidence and associated poor outcomes, the impact of hip fractures on the healthcare system is expected to become increasingly costly.

Interventions provided at different stages after a hip fracture has shown to reduce mortality and improve functional outcomes for the patient. For example, having less than 48 h to surgery after admission could decrease 30-day mortality by 41% and of one-year mortality by 32% [5], and time to surgery is a predictor of achieving independent mobility one week postoperatively [6]. Prophylactic treatment for blood clotting and infection and better operative treatment with fewer technical failures have been shown to result in shorter hospitalisation [4], hence patients were more likely to regain their basic activities of daily life [7]. Post-operative interventions that have been shown to improve function after a hip fracture include home-based rehabilitation [8], comprehensive geriatric care [9], and individualised occupational training [10].

Although surgical and peri-surgical interventions are important to reduce mortality after fracture, a significant number of patients demonstrate permanent disability and dependency even after a successful repair [4]. This points to the importance of post-operative interventions when it comes to improving patients’ outcomes [10]. However, there is a myriad of interventions offered post-surgery across various settings from acute to subacute to community, that aims to improve on different outcomes for patients [4]. There are existing systematic reviews evaluating efficacy of specific post-hip fracture interventions such as occupational therapy [11], electrical stimulation [12], rehabilitation practices [13], lower-limb progressive resistance exercise [14].

In this study, we aim to provide an overview of the available literature on interventions provided post-surgery in the acute, subacute and community settings to improve outcomes for patients with hip fractures using a systematic review. With the consolidation of evidence-based information on wider range of post-hip fracture interventions as compared to previous studies, this study will enable easier comparison by the healthcare providers on the effects of the interventions. The systematic rigorous examination of all the post-hip fracture interventions will allow healthcare providers to easily identify post-operative interventions most pertinent to a patient’s recovery.

## Methods

We performed a systematic literature review guided by the Preferred Reporting Items for Systematic review and Meta-Analysis (PRISMA) 2009 [15]. The PRISMA 2009 checklist can be found in Supplemental Table 1. This study has been registered in Open Science Framework (registration DOI: 10.17605/OSF.IO/2JWEF).

### Search strategy

We identified potentially relevant articles using PubMed®, Embase®, Cochrane Library® and ClinicalTrials.gov searches. Literature review start date was unrestricted and was current as of June 2020. A search strategy (Supplemental Table 2) of two components was used: (1) disease terms and (2) randomized controlled trials (RCTs). The disease terms were adapted from a previously published paper regarding hip fractures [16]. We also reviewed reference lists and searched previous reviews on similar topics.

### Inclusion/exclusion criteria

Two authors (ZYL and WQY) independently screened the titles of selected articles and excluded duplicates and those obviously irrelevant. Two authors reviewed abstracts and full-text articles against prespecified eligibility criteria. We included RCTs of post-surgery interventions conducted in the acute, subacute or community settings, among older patients above 65 years old with any type of non-pathological hip fracture that was surgically treated, and who were able to walk without assistance prior to the fracture. We did not exclude trials that included younger participants if the mean age minus one standard deviation or median age was greater than 65 years.

We also included trials that involved community-dwelling older people who underwent hip fracture surgery. We excluded non–English language articles and abstract-only publications, surgical related RCTs, RCTs with interventions that commenced pre-surgery or immediately upon completion of surgery, blood transfusion, if participants did not undergo surgery, non-randomized trials and animal trials. The references of all selected relevant articles were manually searched to obtain additional relevant publications. Any disagreement was resolved by discussion to reach consensus.

### Data extraction and quality assessment

Two investigators (ZYL and WQY) extracted study data, and another investigator (JKP) verified the accuracy of the data extracted. The data items extracted were: sample size, age, experimental design, characteristics of the intervention in all trial arms including type, dose of therapy and settings, primary and secondary outcome measures and findings.

Two investigators (ZYL and WQY) independently assessed the quality of each study using the Jadad scoring system [17]. The Jadad scale is a scoring system that has three items adding up to a maximum score of 5. Zero, one, or two points can be given for randomization and double-blinding; zero or one point for the description of drop-outs and withdrawals. It should be noted that for Jadad scoring system, double blinding was considered appropriate if it was stated or implied that neither the evaluator nor the subject could identify the intervention being assessed [17].

The Jadad scoring system is relatively straightforward to apply and was chosen because it has been shown to present the best validity and reliability evidence for assessment of methodological quality of RCTs [18]. Given the large number of RCTs of post-surgery interventions among patients with fragility hip fractures, only RCTs with a Jadad score of at least 3 were included in the review. Risk of bias was assessed using Jadad scoring.

### Data presentation

We presented the interventions by types and their settings, as well as the primary and secondary outcome measures, findings and comments for each trial, to allow readers to understand the benefit and anticipated outcome for each type of intervention. We also present the control used in each study. For this review, placebos are defined as inactive substances used to compare results with active substances while sham treatments refer to false treatments for procedures.

## Results

**Fig. 1:**
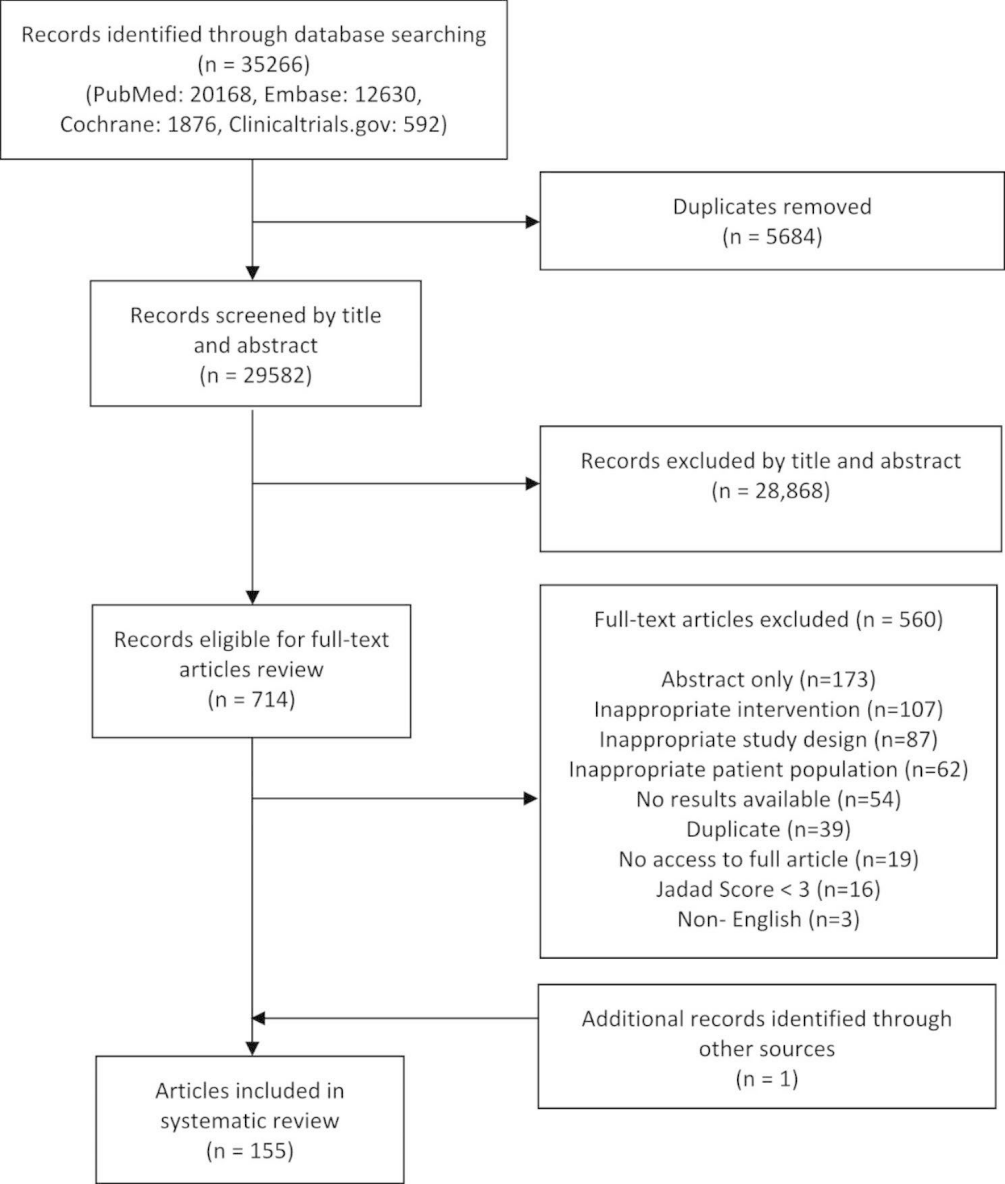
Flow chart on selection of articles for review

As shown in Fig. 1, we identified 35,266 records from our searches in Embase®, PubMed®, Cochrane Library® and ClinicalTrials.gov. After removing 5684 duplicates, 29,582 articles remained. Of these, 714 articles were deemed relevant after title and abstract screening. Of the 715 articles included in full-text screening, 560 articles were excluded. A total of 154 articles met the inclusion criteria. We identified 1 additional article from hand-searching of other sources.

Our search has identified 109 good quality RCTs on post-surgery interventions for patients with fragility hip fractures: 39 RCTs on rehabilitation, 30 that used medication/nutrition/supplementation, 6 RCTs for osteoporosis management, 9 RCTs on optimizing clinical management, 8 RCTs to prevent venous thromboembolism, 4 RCTs to prevent falls, 7 RCTs that used multidisciplinary approaches, 1 RCT on supported discharge, 3 RCTs on managing post-operative anaemia and 2 RCTs on other interventions like group learning and motivational interviewing. These have been categorized according to intervention type and their settings in Table 1, with more details of outcome measures and findings in Supplementary Table 3. The reasons for exclusion at the full-text screening stage can be found in Supplementary Table 4.

**Table 1:** Intervention types and settings of selected RCTs to improve outcomes post hip fracture surgery

RCT intervention type (n)	RCT intervention focus area, intervention group setting		
	Inpatient	Outpatient	Inpatient – Outpatient
Rehabilitation (n = 39) 56 articles	• *Rehabilitation department / ward* ○ Aerobic training [25] ○ Specialised physical rehabilitation [32] ○ Weight bearing exercise [27]* ○ Treadmill training [28, 29] * • *Orthopaedics ward* ○ Progressive strength training [23] * ○ Transcutaneous electrical nerve stimulation [19] • *Geriatric ward* ○ Specialised geriatric rehabilitation [32] ○ Progressive high intensity training [24] • *Trauma unit* ○ Intensive physiotherapy [22] • *Non-specified ward* ○ Neurostimulation [20] ○ Individualized occupational therapy [31] ○ Balance task-specific training [26] ○ Early ambulation [21] ○ Accelerated rehabilitation [30] *	• *Indoor exercise facilities* ○ Progressive resistance training [33, 34] / resistance training [35, 36, 143, 144] • *Outpatient clinic* ○ Strength training [37, 38] / resistance training [39] ○ Physical training and self-efficacy [40] ○ Intensive training [41] * • *Home* ○ Home physiotherapy [8, 42] / strength training [49–51] / high intensity exercises [52] / weight bearing [145, 146] ○ Self-efficacy based exercise [147] ○ Multicomponent home physiotherapy [54] * ○ Telerehabilitation [148] • *Home / institutional care* ○ Weight bearing exercise [53]	• *Inpatient to home* ○ Extended physical therapy plus supplementation [149, 150] ○ Electrical stimulation [55–57] * ○ Individualized occupational therapy [58, 59] ○ Higher dose, weight bearing [151–153] ○ Cognitive behavioural therapy with / without sensor monitoring [61, 62] • *Inpatient to nursing home / home / day hospital* ○ Accelerated rehabilitation [60]
Medication, nutrition and supplementation (n = 30) 38 articles	• *Non-specified ward* ○ Nutritional support [63–65] ○ Dietetic assistants [70] ○ Growth hormone [71] • *Rehabilitation / Orthopedic ward* ○ Nutritional support [66–69] ○ Essential amino acid supplementation [73] ○ Anabolic steroids [72] * ○ Vitamin D [74]	• *Home* ○ Essential amino acid supplementation [76] ○ Vitamin D supplementation [77]	• *Inpatient to home / nursing home / care facility* ○ Growth hormone secretagogue mimetic [88] * ○ Essential amino acid supplementation [75] * ○ Nutritional support [91–93, 154–156] * ○ Nutritional support with dietetic counselling [83, 84] ○ Bisphosphonates [89, 90] * ○ Bone anabolic drug [85–87] ○ Vitamin D loading dose [94–96] * ○ Vitamin D and calcium [157] ○ Anabolic steroids [78–81] ○ Intranasal calcitonin [82]
Optimizing clinical management (n = 9) 11 articles	• *Non-specified ward* ○ Physiotherapy education [97] ○ Management of pressure ulcers [105] * ○ Management of pain [158, 159] ○ Management of postoperative delirium [102] ○ Management of incontinence [103] * ○ Dislocation precautionary measures [104] * • *Post-acute care unit* ○ Enhanced medical rehabilitation [98] • *Geriatric unit* ○ Management of postoperative delirium [101] • *Orthopedic unit* ○ Cognitive functioning / depression management [99, 100] *	Nil	Nil
Prevention of venous thromboembolism (VTE) (n = 8) 9 articles	• *Non-specified ward* ○ Factor inhibitors / antithrombotic [106, 160, 161] * ○ Antithrombotic, factor inhibitor [107, 162] * ○ Ancrod [108]	Nil	• *Inpatient to home / outpatient* ○ Fondaparinux Sodium [109, 110] ○ Antithrombotic [111]
Multidisciplinary, multifactorial programme (n = 7) 13 articles	• *Geriatric Unit* ○ Geriatric vs. orthopedic care [112] * • *Orthopedic rehabilitation* ○ Orthopedic + Geriatric care [113, 114] • *Interdisciplinary care ward* ○ Interdisciplinary care [115] *	• *Home* ○ Multidisciplinary [118, 119] • *Multidisciplinary clinic* ○ Geriatrician, Physiotherapist, Occupational therapist [116, 117] *	• *Inpatient to home* ○ Intensive geriatric rehabilitation [120, 121] ○ Early discharge supported by Geriatric interdisciplinary team [122–124]
Osteoporosis management / Fracture prevention post-discharge (n = 6) 15 articles	Nil	• *Outpatient* ○ Yearly bisphosphonates [163–170] ○ Vitamin D and / or calcium [129] * ○ Osteoporosis case manager [125, 126]	• *Inpatient to home* ○ Primary care and patient empowerment [127] ○ Vitamin D and / or calcium [171, 172] • *Inpatient to outpatient (clinic / primary care)* ○ Osteoporosis management [128]
Falls prevention (inpatient, post-discharge) (n = 4) 7 articles	• *Post-acute Geriatric Rehabilitation Unit* ○ Multicomponent cognitive behavioural intervention [173] ○ Multidisciplinary care [130–133]	• *Home* ○ Follow up call [174]	• *Inpatient and home* ○ Home assessment visit pre-discharge [134]
Post-operative Anaemia (n = 3) 3 articles	• *Non-specified ward* ○ Oral iron therapy [135] *	Nil	• *Inpatient to home* ○ Oral iron therapy [136, 137] *
Supported discharge (n = 1) 1 article	Nil	Nil	• *Inpatient to home* ○ Gerontologic advanced practice nurse care [138]
Others (group learning, motivational interviewing) (n = 2) 2 articles	Nil	• *Unspecified outpatient setting* ○ Group learning and exercise [139] • *Home* ○ Motivational interviewing [140]	Nil
	Total:	109 studies (155 articles)	

*At least one trial with no positive outcome

### Rehabilitation (n = 39)

There are 56 articles reporting 39 unique rehabilitation interventions, consisting of exercises, nerve stimulation, specialized rehabilitation (occupational therapy or geriatric rehabilitation), early rehabilitation, multi-component rehabilitation, self-efficacy and telerehabilitation. Around 46% (n = 18) of rehabilitation interventions were carried out in the outpatient setting, 33% (n = 13) in the inpatient setting and the remaining conducted in transition of care from inpatient to outpatient setting (n = 8).

#### Inpatient

For the 2 studies investigating nerve stimulation interventions in inpatient settings [19, 20], both showed improvements in pain level and functional recovery. In addition, early ambulation as soon as possible on post operative day 1 or 2 showed better functional recovery and reduced length of stay as compared to delayed ambulation commencing on postoperative day 3 or 4 [21].

For exercise interventions (intensive physiotherapy [22], progressive strength training [23, 24], aerobic training [25], balance exercise [26], weight bearing exercise [27], treadmill training [28, 29]) conducted in inpatient settings, the number of interventional therapy sessions ranged from 5 to 15 during the inpatient stay.

A twice weekly progressive quadriceps training conducted over 12 sessions conversely reported a large increase in leg extensor power and reduced disability [24]. Upper body aerobic training and balance exercises showed improvements in functional performance [25], and balance task-specific training also improved pain and quality of life [26]. An intensive physiotherapy that includes 2 additional daily sessions on top of usual care did not improve functional performance but showed better scores in the level of assistance required and reduced hospital length of stay [22].

A study investigating the effects of weight-bearing and non-weight-bearing exercise on strength, balance, gait and functional performance among older inpatients found that there was little difference between groups in the extent of improvement [27]. Another study showed that adaptability treadmill training, conventional treadmill training and usual physical therapy resulted in similar effects on walking ability, fear of falling and fall incidence in older adults rehabilitating from a fall- related hip fracture [28, 29]. Physiotherapy with 5 sessions of strength training using ankle weight cuffs did not demonstrate additional improvements compared to physiotherapy without strength training in reducing the knee-extension strength deficit [23].

The study that involved accelerated rehabilitation by interdisciplinary team did not show any differences in activities of daily living (ADL) or gait outcomes, possibly due to the study’s premature termination [30]. The 2 studies involving occupational therapy or geriatric rehabilitation showed no significant improvement in activities of daily living, walking ability and independence, but showed lower levels of emotional distress from the start of treatment, decreased fatigue [31], lower mortality and more patients who were at home post-treatment [32].

#### Outpatient

There were 18 RCTs investigating rehabilitation performed at outpatient settings, with most demonstrating positive effect in at least one outcome.

For resistance therapy in indoor exercise facility, the studies improvements in strength, physical function and disability [33–36]. For physical training in outpatient clinic, most studies reported positive outcomes in terms of improvement in physical activity [37–40]. One study investigated telerehabilitation in outpatient settings, which reported significant improvements in mobility functions and had good compliance rates [148].

One study reported that 5 home visits by a physiotherapist after patients’ discharge from acute hospital showed greater ambulation ability than 1 month of conventional institution-based rehabilitation [42]. A 6-month home exercise program with 3 home visits by physiotherapist reported a modest improvement in physical function at 6 months for patients who had completed standard rehabilitation, but increased self-efficacy after 6 months [43, 44]. A 12-month individually tailored home rehabilitation program with 5 to 6 home visits by a physiotherapist reported significant improvements in mobility recovery, more apparently in balance and physical function in the long-term rather than short-term [8, 45–48].

The evidence on the effect of strength training interventions on gait and physical function were not consistent, with one study involving a 10-week intervention (twice a week, 30–40 min each session) reporting an improvement along with force production [49], but another intervention lasting 12 months involving strength training (≥ 3 days per week) and aerobics (≥ 2 days per week) reported increase in activity level but no significant improvement in gait and physical function [50, 51].

One study consisting of 20 home visits by physiotherapist reported improved isometric force of the fractured limb in both moderate and high-intensity exercise groups [52]. A study consisting of weight-bearing exercises (at least once a day) for 1 month in the home or institutional care setting reported significant increase in lower limb strength and walking velocity [53].

Multicomponent rehabilitation consisting of home visits by physical therapist supplemented with nutritional counselling; and daily vitamin D (2000 IU), calcium (600 mg), and multivitamins reported no significant improvement in community ambulation, possibly due to insufficient dose of exercise received by some participants as well as low adherence [54]. An 8-week intensive rehabilitation consisting of circuit training concomitant with an individualised balance and gait training programme reported no significant improvement in outcome due to low compliance in the control and study groups [41].

#### Transition of care from inpatient to outpatient

There were 3 studies exploring electrical stimulation interventions in the transition of inpatient to outpatient setting [55–57]. Two studies reported improvements in outcomes, including significant improvements in fracture healing and reduced pain (for pulsed electro-magnetic fields given for at least 8 h/day within 7 days from surgery for 90 days) [57], as well as improvement in recovery of walking speed and better postural stability (for neuromuscular stimulators worn daily for 3 h for 6 weeks commencing 1 week after surgery) [55]. However, electrical stimulation given for 6 weeks (5 days/week as inpatient and twice weekly once discharged, 18 min/session) reported no significant improvement in leg extensor power and disability [56].

There were 2 studies investigating specialized rehabilitation involving occupational therapy which started from inpatient and carried on to the outpatient setting [58, 59], which reported significant improvement in daily functioning, which is not observed in inpatient settings [31, 32]. There was 1 study exploring early rehabilitation in the transition of inpatient to outpatient setting [60], which reported reduction in length of hospital stay, and improvement in functional recovery. This is in line with one study exploring early rehabilitation in inpatient setting [21]. A cognitive behavioral therapy-based occupational therapy coupled with sensor monitoring reported significant improvement in patient reported daily functioning [61, 62].

### Medication, nutrition and supplementation (n = 30)

There are 38 articles reporting 30 medication, nutrition and supplementation interventions which were conducted in inpatient, outpatient and inpatient-outpatient settings. 60% (n = 18) of medication or nutrition supplementation interventions were carried out from inpatient to outpatient setting, 33% (n = 10) in the inpatient setting and the remaining 7% (n = 2) in the outpatient setting.

#### Inpatient

The 10 interventions conducted in inpatient settings investigated nutritional support [63–69], dietetic assistants [70], growth hormone supplementation [71], anabolic steroid [72], essential amino acid supplementation [73] and vitamin D supplementation [74], all of which reported improvement in outcomes (ranging from reduced postoperative complications [63, 64, 65], reduced length of hospital stay [63], improved functional recovery [75], reduced mortality rate [64, 65, 70], improved BMD [74] and reduced falls [74]), except for a study investigating anabolic steroids (nandrolone 2 mg/kg by weekly injection for 4 weeks) which reported minimal benefit on biochemical parameters, grip strength, rehabilitation outcomes, length of stay or functional endpoints [72].

#### Outpatient

There were 2 RCTs conducted in the outpatient settings exploring oral supplementation in addition to home rehabilitative program reported beneficial outcomes. Essential amino acid supplementation was effective in improving function and decreasing disability, in particular sarcopenic patients [76], while vitamin D supplementation reported increased survival rate and reduced medical complications [77].

#### Transition of care from inpatient to outpatient

There were 18 RCTs conducted in the inpatient to outpatient setting. These RCTs yielded mixed results. While RCTs exploring anabolic steroid [78–81], intranasal calcitonin [82], nutritional support with dietetic counselling [83, 84] and bone anabolic drug supplementation [85–87] reported beneficial outcomes, the other interventions yielded negative or mixed results.

One RCT using daily growth hormone secretagogue mimetic [88] for 24 weeks with supplemental vitamin D and multidisciplinary rehabilitation was terminated early due to adverse experiences, making the risk benefit of the drug unacceptable. Two RCTs evaluating bisphosphonate supplementation (35 mg/week), with daily calcium and vitamin D, reported no significant differences in fracture healing, incidence of complications or short-term functional recovery [89, 90].

Studies investigating protein supplementation reported mixed results, with only 2 out of the 5 RCTs reporting conclusive positive outcomes in terms of significant improvement in serum levels of insulin-like growth factor-I and muscle strength, with reduced proximal femur bone loss and length of stay in rehabilitation hospital (intervention: oral protein supplement of 65 g/day, 5 days/week for 6 months) [91], as well as beneficial effects on total body BMD, total hip BMD, hand grip strength, and health-related quality of life (intervention: liquid supplementation with 40 g of protein and 600 kcal daily for six months, in addition to bisphosphonates once weekly for 12 months) [92, 93].

For the 2 RCTs investigating vitamin D loading dose, one RCT with loading dose (250,000 IU) of vitamin D3 within 96 h or up to 7 days post-surgery, with oral maintenance vitamin D3 and calcium, reported higher percentage of replete 25-OHD, reduced rates of falls and reduced pain levels [94, 95]. However, another RCT exploring loading dose (100,000 IU) in addition to daily vitamin D (1,000 IU) had no significant improvement in serum 25-hydroxy vitamin D levels [96].

### Optimizing clinical management (n = 9)

There are 11 articles reporting 9 RCTs on optimization of clinical management interventions in inpatient settings, of which 5 reported positive results.

#### Inpatient

Inpatient physiotherapy education and engagement enhanced with the use of technology and by integrating behavioural skills for therapists into their OT/PT practice has shown to increase patient satisfaction and ability to recall physiotherapy information [97].

Continuous-flow cryocompression therapy (CFCT) applied in the acute recovery phase has demonstrated its effectiveness in reducing pain levels [132, 133]. Integration of a set of behavioural skills for therapists into their OT/PT practice was effective in increasing therapy intensity, therapy engagement, and functional outcomes [98]. In contrary to depression management which did not show any beneficial effect [99, 100], the 2 delirium management interventions reported positive outcomes in terms of significant reduction postoperative delirium [101, 102].

The other interventions with no positive outcomes included multifactorial best practice case management model for incontinence management [103], standard postoperative hip precautions (which reported no significant differences in the risk of dislocation, patient reported outcome and complications) [104], and high-protein nutritional supplement enriched with arginine, zinc and antioxidants dose of 400 ml/day for pressure ulcer management [105].

### Prevention of venous thromboembolism (n = 8)

There are 9 articles reporting 8 RCTs investigating prevention of venous thromboembolism interventions.

#### Inpatient & transition of care from inpatient to outpatient

All interventions for the prevention of venous thromboembolism (VTE) started during inpatient stay during the early acute recovery phases, with 62.5% of the interventions (semuloparin [106], phenindione [107], ancrod [108], fondaparinux sodium [109, 110], enoxaparin [111]) reporting effectiveness in reducing incidence of VTE.

### Multidisciplinary, multifactorial programme (n = 7)

There are 13 articles reporting 7 multidisciplinary, multifactorial programme interventions, with 3 in inpatient settings, and 2 in the outpatient setting and 2 conducted in the inpatient to outpatient setting.

#### Inpatient

There were 3 RCTs conducted in the inpatient setting involving geriatricians for post-surgery care, with the involvement of orthopaedic surgeon / generalist / allied health (physiotherapist, occupation therapist, social worker) / nurse specialist [112–115]. Only the RCT involving geriatrician-generalist-orthopaedic specialist reported a significant reduction in length of hospital stay, and improvement in functional independence and independent living [113, 114].

#### Outpatient

There were 2 RCTs conducted in the outpatient settings. A multidisciplinary post-fracture clinic led by geriatrician with physiotherapist and occupational therapist [116, 117] reported no significant improvement in sedentary time or physical activity. Home visit by a home rehabilitation interdisciplinary team (team coordinator, physiotherapist, occupational therapist, speech pathologist, social worker and therapy aid) and weekly case conferences with a specialist in rehabilitation medicine or a geriatrician, reported significant increase in physical independence and confidence, and reduced caregiver burden [118, 119].

#### Transition of care from inpatient to outpatient

There were 2 RCTs conducted transitioning from inpatient to outpatient settings. Intensive rehabilitation in a geriatric ward combined with occupational therapist evaluation for daily living aids and home visits by physiotherapist, reported several significant improvements including reduced length of hospital stay, increased independence in instrumental activities of daily living (IADL) and more patients with mild or moderate dementia being able to return to community and independent living [120, 121].

Geriatric interdisciplinary home rehabilitation team (geriatrician, nurse, occupational therapist, physiotherapists, social worker and dietician) aiming for early discharge with comprehensive geriatric assessment and frequent home visits during the first days after discharge, reported a significant reduction in length of hospital stay, but not significant improvements in walking ability or reduced complications and readmissions [122–124].

### Osteoporosis management / fracture prevention post-discharge (n = 6)

There are 15 articles reporting 6 osteoporosis management/ fracture prevention interventions. 50% (n = 3) of osteoporosis management/ fracture prevention interventions were carried out in the outpatient setting and the others were conducted in the inpatient to outpatient setting (n = 3).

All RCTs involving post-discharge osteoporosis care management, either through case manager [125, 126], primary care physician [127] or osteoporosis care clinic [128], reported improved osteoporosis management post-discharge. The findings from one study in outpatient settings did not support routine oral supplementation with calcium (1000 mg per day) and vitamin D3 (800 IU per day), either alone or in combination, for the prevention of further fractures in previously mobile elderly people [129].

### Falls prevention (n = 4)

There are 7 articles reporting 4 falls prevention interventions, with 2 in inpatient settings, and 1 each for outpatient and inpatient to outpatient setting. Two RCTs reported positive results in falls prevention – (1) An inpatient geriatric rehabilitation [130–133] and (2) a home visit by an occupational therapist prior to discharge [134]. Interventions in both inpatient and outpatient settings could be valuable in preventing falls and injuries post hip fracture surgery.

### Post-operative anaemia (n = 3)

There are 3 RCTs investigating oral iron therapy for patients post-hip fracture, in which one RCT was conducted in inpatient setting (325 mg/day of oral ferrous sulphate for the duration of the hospitalization [135]), while the other two RCTs were conducted in inpatient to home setting (200 mg oral ferrous sulphate 2 times/day for 28 days [136] and 200 mg oral ferrous sulphate 3 times/day for 28 days [137]). Among the three RCTs, only 1 study (200 mg oral ferrous sulphate 3 times/day for 28 days) reported positive outcome in terms of significant improvement in haemoglobin levels [137].

### Supported discharge (n = 1)

Nursing intervention model consisting of a gerontologic advanced practice nurse (GAPN) post-acute care coordinator for 6 months of care activities reported significant improvements in most ADLs and IADLs (mobility, household chores and personal care) [138]. Care activities included interactions with patients once/week in the first month post-discharge and twice/week until 6 months after surgery, and communication with primary physician, surgeon and staff in various facilities and documenting patients’ progress [138].

### Others (group learning, motivational interviewing) (n = 2)

A 10-week group learning RCT for participants in groups of 5–8, led by a geriatric team (dietician, occupational therapist, physician, physiotherapist and social worker) conducting education on osteoporosis and falls prevention, as well as physical training, reported a significant improvement in the ability to resume meaningful social life and reduced difficulties in ADL [139]. Motivational interviewing by physiotherapist for 8 sessions (30 min/session) reported significant improvements in physical activity, self-efficacy and health-related quality of life, and reduced anxiety and depression [140].

## Discussion

### Principal findings

This review is the first to summarise all the post-hip fracture surgery interventions in the acute, subacute and community settings. The studies presented in this systemic review were heterogenous in terms of settings, interventions, disease, measures used to assess outcomes, and efficacy of the post-hip fracture interventions.

For all the 10 categories of interventions, the evidence base either contains too few trials or contains trials with contradictory findings which preclude any definitive summary. However, for medical practitioners in the acute, subacute and community setting interested in improving certain post-hip fracture outcomes, this review provides comprehensive summary on the possible interventions that may be useful. The interventions in this review had minor or no side effects reported. Notable adverse reactions were observed in patients treated with growth hormone secretagogue mimetic MK-0677 [88], which led to early termination of the trial.

For some studies, the lack of positive outcomes may be attributed to the low compliance to the intervention. For example, the authors suggested that the lack of significant improvement in community ambulation after home visits by physiotherapist may be due to insufficient dose of exercise received by some participants as well as lower adherence rate in this group [54].

It was also postulated that an 8-week intensive rehabilitation consisting of circuit training concomitant with an individualised balance and gait training programme reported no significant improvement in rehabilitation due to low compliance in the control and study groups [41]. This highlights the need to consider feasibility, and monitor the fidelity and compliance to the intervention in future studies for more conclusive evidence on the efficacy or effectiveness of the post- hip fracture interventions.

### Comparison to prior work

In this review, we found that RCTs involving post-discharge osteoporosis care management, either through case manager [126,125], primary care physician 127] or osteoporosis care clinic [128], reported improved osteoporosis management. Except for a RCT investigating multidisciplinary post-fracture clinic led by geriatrician with physiotherapist and occupational therapist [116, 117] which reported no significant improvement in sedentary time or physical activity, the other RCTs reported the positive effects of occupational therapy on daily functions and emotions, which is in agreement with findings from another systematic review [11].

For the 2 RCTs involving nerve stimulation in inpatient settings, both showed improvements in pain level and functional recovery, suggesting that nerve stimulation could play a valuable role in reducing the length of inpatient stay [20], and the effect of nerve stimulation on long-term functional outcomes should be explored further [19].

A recent study has shown that patients who underwent a blood transfusion had lower preoperative haemoglobin levels and longer durations of surgical treatment [141]. Our review included three RCTs investigating post-operative anaemia, and only 1 study (200 mg oral ferrous sulphate 3 times/day for 28 days) reported positive outcome in terms of significant improvement in haemoglobin levels [137].

A recent review on physical therapy for patients with femoral neck fracture has found that the most effective intervention appears to be exercise of progressive resistance [142]. This aligns with the findings from studies by Binder et al. [33] and Host et al. [34], both included in this review, which demonstrated that extended outpatient rehabilitation that includes progressive resistance training improves physical function.

### Strengths and Limitations

The findings from this review can aid in clinical practice by allowing formulation of thematic program with combination of interventions as part of bundled care to improve outcome for patients who have undergone hip fracture surgery. For example, nerve stimulation could be made available for patients who have undergone hip fracture surgery in the inpatient settings, followed by post-discharge outpatient osteoporosis care management.

However, future research on optimal duration and dosage/ frequency of each type of intervention may be warranted as our review has demonstrated that the beneficial effect of the exercise intervention did not seem to be correlated to duration of exercise. For example, one study involving a 10-week intervention (twice a week, 30–40 min each session) reporting an improvement along with force production [49], but another intervention lasting 12 months involving strength training (≥ 3 days per week) and aerobics (≥ 2 days per week) reported increase in activity level but no significant improvement in gait and physical function [50, 51].

There are limitations in this study. Firstly, publication bias may result in overestimation of the efficacy or effectiveness of the post-hip fracture surgery interventions. In addition, study design e.g., dosage, frequency and duration of treatment was heterogenous among the studies included in this review, making it difficult to compare results across studies. The heterogeneities of the follow-up period, enrolment time after fracture, and variable outcomes also preclude the possibility of performing a meta-analysis in this study.

Moreover, as the search in the scholarly literature was restricted to articles published before June 2020, our review may have excluded studies published after the cut-off date. Nevertheless, the findings from this review can serve as foundation for future research on post-surgery interventions for hip fracture.

Lastly, the Jadad scale may have shortcomings such as incompleteness and the use of an additive (not multiplicative) scoring system that allows compensation for weaknesses [175]. However, previous studies have demonstrated the reliability and validity of the Jadad scale [176, 177].

## Conclusion

The identified RCTs regarding post-hip fracture surgery interventions were heterogeneous in terms of type of interventions, settings and outcome measures. Post-hip fracture surgery interventions should span from acute inpatient to post-discharge outpatient care as part of bundled treatment for patients to achieve better improvement in outcomes such as improved physical function recovery, lower rate of complications, and shortening of length of stay. For example, nutritional supplementation could be made available for patients who have undergone hip fracture surgery in the inpatient settings, followed by post-discharge outpatient osteoporosis care management. The findings from this review can aid clinicians and researchers by allowing formulation of thematic program with combination of interventions as part of bundled care to improve outcome for patients who have undergone hip fracture surgery.

## Electronic supplementary material

Below is the link to the electronic supplementary material.

Supplementary Material 1

## Data Availability

All data generated or analyzed during this study are included in this published article and its supplementary information files.

## References

[1] Gullberg B, Johnell O, Kanis JA (1997). World-wide projections for hip fracture. Osteoporos Int.

[2] Cooper C, Campion G, Melton LJ (1992). Hip fractures in the elderly: a world-wide projection. Osteoporos Int.

[3] Leibson CL, Tosteson ANA, Gabriel SE (2002). Mortality, disability, and nursing home use for persons with and without hip fracture: a population-based study. J Am Geriatr Soc.

[4] Craik RL (1994). Disability following hip fracture. Phys Ther.

[5] Shiga T, Wajima Z, Ohe Y (2008). Is operative delay associated with increased mortality of hip fracture patients? Systematic review, meta-analysis, and meta-regression. Can J Anaesth.

[6] Fitzgerald M, Blake C, Askin D (2018). Mobility one week after a hip fracture - can it be predicted?. Int J Orthop Trauma Nurs.

[7] Moerman S, Mathijssen NM, Tuinebreijer WE (2018). Less than one-third of hip fracture patients return to their prefracture level of instrumental activities of daily living in a prospective cohort study of 480 patients. Geriatr Gerontol Int.

[8] Edgren J, Salpakoski A, Sihvonen SE (2015). Effects of a home-based physical rehabilitation program on physical disability after hip fracture: a randomized controlled trial. J Am Med Dir Assoc.

[9] Prestmo A, Saltvedt I, Helbostad JL (2016). Who benefits from orthogeriatric treatment? Results from the Trondheim hip-fracture trial. BMC Geriatr.

[10] Alexiou KI, Roushias A, Varitimidis SE, Malizos KN (2018). Quality of life and psychological consequences in elderly patients after a hip fracture: a review. Clin Interv Aging.

[11] Lee SY, Jung SH, Lee S-U (2019). Is occupational therapy after hip fracture surgery effective in improving function?: a systematic review and meta-analysis of randomized controlled studies. Am J Phys Med Rehabil.

[12] Davison P, Wilkinson R, Miller J, Auais M. A systematic review of using electrical stimulation to improve clinical outcomes after hip fractures. null. 2021;1–19. 10.1080/09593985.2021.1894620. 10.1080/09593985.2021.1894620 33890541

[13] Chudyk AM, Jutai JW, Petrella RJ, Speechley M (2009). Systematic review of hip fracture rehabilitation practices in the elderly. Arch Phys Med Rehabil.

[14] Lee SY, Yoon B-H, Beom J (2017). Effect of lower-limb progressive resistance exercise after hip fracture surgery: a systematic review and meta-analysis of randomized controlled studies. J Am Med Dir Assoc.

[15] Moher D, Liberati A, Tetzlaff J, Altman DG (2009). Preferred reporting items for systematic reviews and meta-analyses: the PRISMA statement. BMJ.

[16] Guay J, Parker MJ, Griffiths R, Kopp S (2017). Peripheral nerve blocks for hip fractures. Cochrane Database Syst Rev.

[17] Jadad AR, Moore RA, Carroll D (1996). Assessing the quality of reports of randomized clinical trials: is blinding necessary?. Control Clin Trials.

[18] Olivo SA, Macedo LG, Gadotti IC (2008). Scales to assess the quality of randomized controlled trials: a systematic review. Phys Ther.

[19] Elboim-Gabyzon M, Andrawus Najjar S, Shtarker H (2019). Effects of transcutaneous electrical nerve stimulation (TENS) on acute postoperative pain intensity and mobility after hip fracture: a double-blinded, randomized trial. Clin Interv Aging.

[20] Gorodetskyi IG, Gorodnichenko AI, Tursin PS (2007). Non-invasive interactive neurostimulation in the post-operative recovery of patients with a trochanteric fracture of the femur. A randomised, controlled trial. J Bone Joint Surg Br.

[21] Oldmeadow LB, Edwards ER, Kimmel LA (2006). No rest for the wounded: early ambulation after hip surgery accelerates recovery. ANZ J Surg.

[22] Kimmel LA, Liew SM, Sayer JM, Holland AE (2016). HIP4Hips (high intensity physiotherapy for hip fractures in the acute hospital setting): a randomised controlled trial. Med J Aust.

[23] Kronborg L, Bandholm T, Palm H (2017). Effectiveness of acute in-hospital physiotherapy with knee-extension strength training in reducing strength deficits in patients with a hip fracture: a randomised controlled trial. PLoS ONE.

[24] Mitchell SL, Stott DJ, Martin BJ, Grant SJ (2001). Randomized controlled trial of quadriceps training after proximal femoral fracture. Clin Rehabil.

[25] Mendelsohn ME, Overend TJ, Connelly DM, Petrella RJ (2008). Improvement in aerobic fitness during rehabilitation after hip fracture. Arch Phys Med Rehabil.

[26] Monticone M, Ambrosini E, Brunati R (2018). How balance task-specific training contributes to improving physical function in older subjects undergoing rehabilitation following hip fracture: a randomized controlled trial. Clin Rehabil.

[27] Sherrington C, Lord SR, Herbert RD (2003). A randomised trial of weight-bearing versus non-weight-bearing exercise for improving physical ability in inpatients after hip fracture. Aust J Physiother.

[28] van Ooijen MW, Roerdink M, Trekop M (2013). Functional gait rehabilitation in elderly people following a fall-related hip fracture using a treadmill with visual context: design of a randomized controlled trial. BMC Geriatr.

[29] van Ooijen MW, Roerdink M, Trekop M, et al. The efficacy of treadmill training with and without projected visual context for improving walking ability and reducing fall incidence and fear of falling in older adults with fall-related hip fracture: a randomized controlled trial. BMC Geriatr. 2016;16. 10.1186/s12877-016-0388-x. 10.1186/s12877-016-0388-x PMC5198499 28031021

[30] Uy C, Kurrle SE, Cameron ID (2008). Inpatient multidisciplinary rehabilitation after hip fracture for residents of nursing homes: a randomised trial. Australas J Ageing.

[31] Martín-Martín LM, Valenza-Demet G, Jiménez-Moleón JJ (2014). Effect of occupational therapy on functional and emotional outcomes after hip fracture treatment: a randomized controlled trial. Clin Rehabil.

[32] Lahtinen A, Leppilahti J, Harmainen S (2015). Geriatric and physically oriented rehabilitation improves the ability of independent living and physical rehabilitation reduces mortality: a randomised comparison of 538 patients. Clin Rehabil.

[33] Binder EF, Brown M, Sinacore DR (2004). Effects of extended outpatient rehabilitation after hip fracture: a randomized controlled trial. JAMA.

[34] Host HH, Sinacore DR, Bohnert KL (2007). Training-induced strength and functional adaptations after hip fracture. Phys Ther.

[35] Portegijs E, Kallinen M, Rantanen T (2008). Effects of resistance training on lower-extremity impairments in older people with hip fracture. Arch Phys Med Rehabil.

[36] Edgren J, Rantanen T, Heinonen A (2012). Effects of progressive resistance training on physical disability among older community-dwelling people with history of hip fracture. Aging Clin Exp Res.

[37] Sylliaas H, Brovold T, Wyller TB, Bergland A (2011). Progressive strength training in older patients after hip fracture: a randomised controlled trial. Age Ageing.

[38] Sylliaas H, Brovold T, Wyller TB, Bergland A (2012). Prolonged strength training in older patients after hip fracture: a randomised controlled trial. Age Ageing.

[39] Singh NA, Quine S, Clemson LM (2012). Effects of high-intensity progressive resistance training and targeted multidisciplinary treatment of frailty on mortality and nursing home admissions after hip fracture: a randomized controlled trial. J Am Med Dir Assoc.

[40] Suwanpasu S, Aungsuroch Y, Jitpanya C (2014). Post-surgical physical activity enhancing program for elderly patients after hip fracture: a randomized controlled trial. Asian Biomed.

[41] Peterson MGE, Ganz SB, Allegrante JP, Cornell CN (2004). High-intensity Exercise Training following hip fracture. Top Geriatric Rehabilitation.

[42] Kuisma R (2002). A randomized, controlled comparison of home versus institutional rehabilitation of patients with hip fracture. Clin Rehabil.

[43] Latham NK, Harris BA, Bean JF (2014). Effect of a home-based exercise program on functional recovery following rehabilitation after hip fracture: a randomized clinical trial. JAMA.

[44] Chang F-H, Latham NK, Ni P, Jette AM (2015). Does self-efficacy mediate functional change in older adults participating in an exercise program after hip fracture? A randomized controlled trial. Arch Phys Med Rehabil.

[45] Salpakoski A, Törmäkangas T, Edgren J (2014). Effects of a multicomponent home-based physical rehabilitation program on mobility recovery after hip fracture: a randomized controlled trial. J Am Med Dir Assoc.

[46] Turunen K, Salpakoski A, Edgren J (2017). Physical activity after a hip fracture: Effect of a Multicomponent Home-Based Rehabilitation Program-A secondary analysis of a Randomized Controlled Trial. Arch Phys Med Rehabil.

[47] ss TH, Edgren J, Salpakoski A (2019). Effects of a home-based physical Rehabilitation Program on tibial bone structure, density, and Strength after hip fracture: a secondary analysis of a Randomized Controlled Trial. JBMR Plus.

[48] Portegijs E, Rantakokko M, Edgren J (2013). Effects of a rehabilitation program on perceived environmental barriers in older patients recovering from hip fracture: a randomized controlled trial. Biomed Res Int.

[49] Mangione KK, Craik RL, Palombaro KM (2010). Home-based leg-strengthening exercise improves function 1 year after hip fracture: a randomized controlled study. J Am Geriatr Soc.

[50] Orwig DL, Hochberg M, Yu-Yahiro J (2011). Delivery and outcomes of a yearlong home exercise program after hip fracture: a randomized controlled trial. Arch Intern Med.

[51] Yu-Yahiro JA, Resnick B, Orwig D (2009). Design and implementation of a home-based exercise program post-hip fracture: the Baltimore hip studies experience. PM R.

[52] Mangione KK, Craik RL, Tomlinson SS, Palombaro KM (2005). Can elderly patients who have had a hip fracture perform moderate- to high-intensity exercise at home?. Phys Ther.

[53] Sherrington C, Lord SR (1997). Home exercise to improve strength and walking velocity after hip fracture: a randomized controlled trial. Arch Phys Med Rehabil.

[54] Magaziner J, Mangione KK, Orwig D (2019). Effect of a Multicomponent Home-Based physical therapy intervention on Ambulation after hip fracture in older adults. JAMA.

[55] Lamb SE, Oldham JA, Morse RE, Evans JG (2002). Neuromuscular stimulation of the quadriceps muscle after hip fracture: a randomized controlled trial. Arch Phys Med Rehabil.

[56] Braid V, Barber M, Mitchell SL (2008). Randomised controlled trial of electrical stimulation of the quadriceps after proximal femoral fracture. Aging Clin Exp Res.

[57] Faldini C, Cadossi M, Luciani D (2010). Electromagnetic bone growth stimulation in patients with femoral neck fractures treated with screws: prospective randomized double-blind study. Curr Orthop Pract.

[58] Hagsten B, Svensson O, Gardulf A (2004). Early individualized postoperative occupational therapy training in 100 patients improves ADL after hip fracture: a randomized trial. Acta Orthop Scand.

[59] Hagsten B, Svensson O, Gardulf A (2006). Health-related quality of life and self-reported ability concerning ADL and IADL after hip fracture: a randomized trial. Acta Orthop.

[60] Cameron ID, Lyle DM, Quine S (1993). Accelerated rehabilitation after proximal femoral fracture: a randomized controlled trial. Disabil Rehabil.

[61] Pol MC, Ter Riet G, van Hartingsveldt M (2017). Effectiveness of sensor monitoring in an occupational therapy rehabilitation program for older individuals after hip fracture, the SO-HIP trial: study protocol of a three-arm stepped wedge cluster randomized trial. BMC Health Serv Res.

[62] Pol MC, Ter Riet G, van Hartingsveldt M (2019). Effectiveness of sensor monitoring in a rehabilitation programme for older patients after hip fracture: a three-arm stepped wedge randomised trial. Age Ageing.

[63] Anbar R, Beloosesky Y, Cohen J (2014). Tight calorie control in geriatric patients following hip fracture decreases complications: a randomized, controlled study. Clin Nutr.

[64] Eneroth M, Olsson U-B, Thorngren K-G (2005). Insufficient fluid and energy intake in hospitalised patients with hip fracture. A prospective randomised study of 80 patients. Clin Nutr.

[65] Eneroth M, Olsson U-B, Thorngren K-G (2006). Nutritional supplementation decreases hip fracture-related complications. Clin Orthop Relat Res.

[66] Malafarina V, Uriz-Otano F, Gil-Guerrero L (2013). Study protocol: high-protein nutritional intervention based on β-hydroxy-β-methylbutirate, vitamin D3 and calcium on obese and lean aged patients with hip fractures and sarcopenia. The HIPERPROT-GER study. Maturitas.

[67] Malafarina V, Uriz-Otano F, Malafarina C (2017). Effectiveness of nutritional supplementation on sarcopenia and recovery in hip fracture patients. A multi-centre randomized trial. Maturitas.

[68] Mw M et al. J W, E W, (2013) Clinical benefits of oral nutritional supplementation for elderly hip fracture patients: a single blind randomised controlled trial. In: Age and ageing. https://pubmed.ncbi.nlm.nih.gov/22685164/. Accessed 11 Feb 2021. 10.1093/ageing/afs078 22685164

[69] Niitsu M, Ichinose D, Hirooka T (2016). Effects of combination of whey protein intake and rehabilitation on muscle strength and daily movements in patients with hip fracture in the early postoperative period. Clin Nutr.

[70] Duncan DG, Beck SJ, Hood K, Johansen A (2006). Using dietetic assistants to improve the outcome of hip fracture: a randomised controlled trial of nutritional support in an acute trauma ward. Age Ageing.

[71] Hedström M, Sääf M, Brosjö E (2004). Positive effects of short-term growth hormone treatment on lean body mass and BMC after a hip fractureA double-blind placebo-controlled pilot study in 20 patients. Acta Orthop Scand.

[72] Sloan JP, Wing P, Dian L, Meneilly GS (1992). A pilot study of anabolic steroids in Elderly patients with hip fractures. J Am Geriatr Soc.

[73] Rondanelli M, Guido D, Faliva MA (2020). Effects of essential amino acid supplementation on pain in the elderly with hip fractures: a pilot, double-blind, placebo-controlled, randomised clinical trial. J Biol Regul Homeost Agents.

[74] Harwood RH, Sahota O, Gaynor K (2004). A randomised, controlled comparison of different calcium and vitamin D supplementation regimens in elderly women after hip fracture: the Nottingham Neck of Femur (NONOF) Study. Age Ageing.

[75] Aquilani R, Zuccarelli GC, Condino AM, et al. Despite inflammation, supplemented essential amino acids may improve circulating levels of Albumin and Haemoglobin in patients after hip fractures. Nutrients. 2017;9. 10.3390/nu9060637. 10.3390/nu9060637 PMC5490616 28635634

[76] Invernizzi M, de Sire A, D’Andrea F (2019). Effects of essential amino acid supplementation and rehabilitation on functioning in hip fracture patients: a pilot randomized controlled trial. Aging Clin Exp Res.

[77] Laiz A, Malouf J, Marin A (2017). Impact of 3-Monthly vitamin D Supplementation Plus Exercise on Survival after surgery for osteoporotic hip fracture in adult patients over 50 years: a pragmatic Randomized, partially blinded, controlled trial. J Nutr Health Aging.

[78] Tidermark J, Ponzer S, Carlsson P (2004). Effects of protein-rich supplementation and nandrolone in lean elderly women with femoral neck fractures. Clin Nutr.

[79] Tengstrand B, Cederholm T, Söderqvist A, Tidermark J (2007). Effects of protein-rich supplementation and nandrolone on bone tissue after a hip fracture. Clin Nutr.

[80] Carlsson P, Tidermark J, Ponzer S (2005). Food habits and appetite of elderly women at the time of a femoral neck fracture and after nutritional and anabolic support. J Hum Nutr Diet.

[81] Hedström M, Sjöberg K, Brosjö E (2002). Positive effects of anabolic steroids, vitamin D and calcium on muscle mass, bone mineral density and clinical function after a hip fracture. A randomised study of 63 women. J Bone Joint Surg Br.

[82] Huusko TM, Karppi P, Kautiainen H (2002). Randomized, double-blind, clinically controlled trial of intranasal calcitonin treatment in patients with hip fracture. Calcif Tissue Int.

[83] Wyers CE, Breedveld-Peters JJ, Reijven PL (2010). Efficacy and cost-effectiveness of nutritional intervention in elderly after hip fracture: design of a randomized controlled trial. BMC Public Health.

[84] Wyers CE, Reijven PLM, Breedveld-Peters JJL (2018). Efficacy of nutritional intervention in Elderly after hip fracture: a Multicenter Randomized Controlled Trial. J Gerontol A Biol Sci Med Sci.

[85] Bhandari M, Jin L, See K (2016). Does Teriparatide improve femoral Neck Fracture Healing: results from a randomized placebo-controlled trial. Clin Orthop Relat Res.

[86] Aspenberg P, Malouf J, Tarantino U (2016). Effects of Teriparatide compared with Risedronate on Recovery after Pertrochanteric Hip fracture: results of a Randomized, Active-Controlled, double-blind clinical trial at 26 weeks. J Bone Joint Surg Am.

[87] Malouf-Sierra J, Tarantino U, García-Hernández PA (2017). Effect of Teriparatide or Risedronate in Elderly patients with a recent pertrochanteric hip fracture: final results of a 78-Week randomized clinical trial. J Bone Miner Res.

[88] Adunsky A, Chandler J, Heyden N (2011). MK-0677 (ibutamoren mesylate) for the treatment of patients recovering from hip fracture: a multicenter, randomized, placebo-controlled phase IIb study. Arch Gerontol Geriatr.

[89] Kim T-Y, Ha Y-C, Kang B-J (2012). Does early administration of bisphosphonate affect fracture healing in patients with intertrochanteric fractures?. J Bone Joint Surg Br volume.

[90] Unnanuntana A, Laohaprasitiporn P, Jarusriwanna A. Effect of bisphosphonate initiation at week 2 versus week 12 on short-term functional recovery after femoral neck fracture: a randomized controlled trial. Arch Osteoporos. 2017;12. 10.1007/s11657-017-0321-8. 10.1007/s11657-017-0321-8 PMC5346124 28283937

[91] Schürch MA, Rizzoli R, Slosman D (1998). Protein supplements increase serum insulin-like growth factor-I levels and attenuate proximal femur bone loss in patients with recent hip fracture. A randomized, double-blind, placebo-controlled trial. Ann Intern Med.

[92] Flodin L, Sääf M, Cederholm T (2014). Additive effects of nutritional supplementation, together with bisphosphonates, on bone mineral density after hip fracture: a 12-month randomized controlled study. Clin Interv Aging.

[93] Flodin L, Cederholm T, Sääf M, et al. Effects of protein-rich nutritional supplementation and bisphosphonates on body composition, handgrip strength and health-related quality of life after hip fracture: a 12-month randomized controlled study. BMC Geriatr. 2015;15. 10.1186/s12877-015-0144-7. 10.1186/s12877-015-0144-7 PMC4647612 26572609

[94] Mak JCS, Mason R, Klein L, Cameron ID (2011). Improving mobility and reducing disability in older people through early high-dose vitamin D replacement following hip fracture: a protocol for a randomized controlled trial and economic evaluation. Geriatr Orthop Surg Rehabil.

[95] Mak JC, Mason RS, Klein L, Cameron ID. An initial loading-dose vitamin D versus placebo after hip fracture surgery: randomized trial. BMC Musculoskelet Disord. 2016;17. 10.1186/s12891-016-1174-9. 10.1186/s12891-016-1174-9 PMC4982117 27515154

[96] Papaioannou A, Kennedy CC, Giangregorio L (2011). A randomized controlled trial of vitamin D dosing strategies after acute hip fracture: no advantage of loading doses over daily supplementation. BMC Musculoskelet Disord.

[97] Dallimore R-K, Asinas-Tan ML, Chan D (2017). A randomised, double-blinded clinical study on the efficacy of multimedia presentation using an iPad for patient education of postoperative hip surgery patients in a public hospital in Singapore. Singap Med J.

[98] Lenze EJ, Host HH, Hildebrand MW (2012). Enhanced medical rehabilitation increases therapy intensity and engagement and improves functional outcomes in postacute rehabilitation of older adults: a randomized-controlled trial. J Am Med Dir Assoc.

[99] Oude Voshaar RC, Banerjee S, Horan M (2006). Fear of falling more important than pain and depression for functional recovery after surgery for hip fracture in older people. Psychol Med.

[100] Burns A, Banerjee S, Morris J (2007). Treatment and prevention of depression after surgery for hip fracture in older people: randomized, controlled trials. J Am Geriatr Soc.

[101] Lundström M, Olofsson B, Stenvall M (2007). Postoperative delirium in old patients with femoral neck fracture: a randomized intervention study. Aging Clin Exp Res.

[102] Papadopoulos G, Pouangare M, Papathanakos G (2014). The effect of ondansetron on postoperative delirium and cognitive function in aged orthopedic patients. Minerva Anestesiol.

[103] Parkinson L, Chiarelli P, Byrne J (2007). Continence promotion for older hospital patients following surgery for fractured neck of femur: pilot of a randomized controlled trial. Clin Interv Aging.

[104] Jobory A, Rolfson O, Åkesson KE (2019). Hip precautions not meaningful after hemiarthroplasty due to hip fracture. Cluster-randomized study of 394 patients operated with direct anterolateral approach. Injury.

[105] Houwing RH, Rozendaal M, Wouters-Wesseling W (2003). A randomised, double-blind assessment of the effect of nutritional supplementation on the prevention of pressure ulcers in hip-fracture patients. Clin Nutr.

[106] Fisher WD, Agnelli G, George DJ (2013). Extended venous thromboembolism prophylaxis in patients undergoing hip fracture surgery - the SAVE-HIP3 study. Bone Joint J.

[107] Hamilton HW, Crawford JS, Gardiner JH, Wiley AM (1970). Venous thrombosis in patients with fracture of the upper end of the femur. J Bone Joint Surg Br volume.

[108] Lowe GD, Campbell AF, Meek DR (1978). Subcutaneous ancrod in prevention of deep-vein thrombosis after operation for fractured neck of femur. Lancet.

[109] Eriksson BI, Lassen MR, PENTasaccharide in HIp-FRActure Surgery Plus Investigators (2003). Duration of prophylaxis against venous thromboembolism with fondaparinux after hip fracture surgery: a multicenter, randomized, placebo-controlled, double-blind study. Arch Intern Med.

[110] Dobesh PP (2003). Novel concepts: emerging data and the role of extended prophylaxis following hip fracture surgery. Am J Health Syst Pharm.

[111] Tang Y, Wang K, Shi Z (2017). A RCT study of Rivaroxaban, low-molecular-weight heparin, and sequential medication regimens for the prevention of venous thrombosis after internal fixation of hip fracture. Biomed Pharmacother.

[112] Galvard H, Samuelsson SM (1995). Orthopedic or geriatric rehabilitation of hip fracture patients: a prospective, randomized, clinically controlled study in Malmö. Swed Aging (Milano).

[113] Kennie DC, Reid J, Richardson IR (1988). Effectiveness of geriatric rehabilitative care after fractures of the proximal femur in elderly women: a randomised clinical trial. BMJ.

[114] Reid J, Kennie DC (1989). Geriatric rehabilitative care after fractures of the proximal femur: one year follow up of a randomised clinical trial. BMJ.

[115] Naglie G, Tansey C, Kirkland JL (2002). Interdisciplinary inpatient care for elderly people with hip fracture: a randomized controlled trial. CMAJ.

[116] Cook WL, Khan KM, Bech MH (2011). Post-discharge management following hip fracture - get you back to B4: a parallel group, randomized controlled trial study protocol. BMC Geriatr.

[117] Zusman EZ, Dawes M, Fleig L (2019). Older adults’ sedentary behavior and physical activity after hip fracture: results from an Outpatient Rehabilitation Randomized Controlled Trial. J Geriatr Phys Ther.

[118] Crotty M, Whitehead CH, Gray S, Finucane PM (2002). Early discharge and home rehabilitation after hip fracture achieves functional improvements: a randomized controlled trial. Clin Rehabil.

[119] Crotty M, Whitehead C, Miller M, Gray S (2003). Patient and caregiver outcomes 12 months after home-based therapy for hip fracture: a randomized controlled trial. Arch Phys Med Rehabil.

[120] Huusko TM, Karppi P, Avikainen V (2000). Randomised, clinically controlled trial of intensive geriatric rehabilitation in patients with hip fracture: subgroup analysis of patients with dementia. BMJ.

[121] Huusko TM, Karppi P, Avikainen V (2002). Intensive geriatric rehabilitation of hip fracture patients: a randomized, controlled trial. Acta Orthop Scand.

[122] Karlsson Ã, Berggren M, Gustafson Y (2016). Effects of Geriatric Interdisciplinary Home Rehabilitation on walking ability and length of Hospital stay after hip fracture: a Randomized Controlled Trial. J Am Med Dir Assoc.

[123] Karlsson Ã, Lindelöf N, Olofsson B (2020). Effects of Geriatric Interdisciplinary Home Rehabilitation on Independence in Activities of Daily living in older people with hip fracture: a Randomized Controlled Trial. Arch Phys Med Rehabil.

[124] Berggren M, Karlsson Ã, Lindelöf N (2019). Effects of geriatric interdisciplinary home rehabilitation on complications and readmissions after hip fracture: a randomized controlled trial. Clin Rehabil.

[125] Majumdar SR, Beaupre LA, Harley CH (2007). Use of a case manager to improve osteoporosis treatment after hip fracture: results of a randomized controlled trial. Arch Intern Med.

[126] Beaupre LA, Morrish DW, Hanley DA (2011). Oral bisphosphonates are associated with reduced mortality after hip fracture. Osteoporos Int.

[127] Davis JC, Guy P, Ashe MC (2007). HipWatch: osteoporosis investigation and treatment after a hip fracture: a 6-month randomized controlled trial. J Gerontol A Biol Sci Med Sci.

[128] Miki RA, Oetgen ME, Kirk J, Insogna KL, Lindskog DM.Orthopaedic management improves the rate of early osteoporosis treatment after hip fracture. A randomized clinical trial. In: The Journal of bone and joint surgery. 2008 Nov 1;90(11):2346–53. 10.2106/JBJS.G.01246. 10.2106/JBJS.G.01246 18978403

[129] Grant AM, Avenell A, Campbell MK (2005). Oral vitamin D3 and calcium for secondary prevention of low-trauma fractures in elderly people (randomised evaluation of calcium or vitamin D, RECORD): a randomised placebo-controlled trial. Lancet.

[130] Stenvall M, Olofsson B, Lundström M (2007). A multidisciplinary, multifactorial intervention program reduces postoperative falls and injuries after femoral neck fracture. Osteoporos Int.

[131] Stenvall M, Olofsson B, Nyberg L (2007). Improved performance in activities of daily living and mobility after a multidisciplinary postoperative rehabilitation in older people with femoral neck fracture: a randomized controlled trial with 1-year follow-up. J Rehabil Med.

[132] Berggren M, Stenvall M, Olofsson B, Gustafson Y (2008). Evaluation of a fall-prevention program in older people after femoral neck fracture: a one-year follow-up. Osteoporos Int.

[133] Olofsson B, Stenvall M, Lundström M (2007). Malnutrition in hip fracture patients: an intervention study. J Clin Nurs.

[134] Lockwood KJ, Harding KE, Boyd JN, Taylor NF (2019). Predischarge home visits after hip fracture: a randomized controlled trial. Clin Rehabil.

[135] Zauber NP, Zauber AG, Gordon FJ (1992). Iron supplementation after femoral head replacement for patients with normal iron stores. JAMA.

[136] Parker MJ (2010). Iron supplementation for anemia after hip fracture surgery: a randomized trial of 300 patients. J Bone Joint Surg Am.

[137] Prasad N, Rajamani V, Hullin D, Murray JM (2009). Post-operative anaemia in femoral neck fracture patients: does it need treatment? A single blinded prospective randomised controlled trial. Injury.

[138] Krichbaum K (2007). GAPN postacute care coordination improves hip fracture outcomes. West J Nurs Res.

[139] Elinge E, Löfgren B, Gagerman E, Nyberg L (2003). A Group Learning Programme for Old people with hip fracture: a randomized study. Scand J Occup Ther.

[140] O’Halloran PD, Shields N, Blackstock F (2016). Motivational interviewing increases physical activity and self-efficacy in people living in the community after hip fracture: a randomized controlled trial. Clin Rehabil.

[141] Testa G, Montemagno M, Vescio A (2023). Blood-transfusion risk factors after Intramedullary nailing for extracapsular femoral Neck fracture in Elderly Patients. J Funct Morphology Kinesiol.

[142] Avola M, Mangano GRA, Testa G (2020). Rehabilitation strategies for patients with femoral neck fractures in sarcopenia: a narrative review. J Clin Med.

[143] Portegijs E, Read S, Pakkala I (2014). Sense of coherence: effect on adherence and response to resistance training in older people with hip fracture history. J Aging Phys Act.

[144] Pakkala I, Read S, Sipilä S (2012). Effects of intensive strength-power training on sense of coherence among 60-85-year-old people with hip fracture: a randomized controlled trial. Aging Clin Exp Res.

[145] Sherrington C, Lord SR, Herbert RD (2004). A randomized controlled trial of weight-bearing versus non-weight-bearing exercise for improving physical ability after usual care for hip fracture. Arch Phys Med Rehabil.

[146] Taraldsen K, Thingstad P, Døhl Ø (2019). Short and long-term clinical effectiveness and cost-effectiveness of a late-phase community-based balance and gait exercise program following hip fracture. The EVA-Hip Randomised Controlled Trial. PLoS ONE.

[147] Resnick B, Orwig D, Yu-Yahiro J (2007). Testing the effectiveness of the exercise plus program in older women post-hip fracture. Ann Behav Med.

[148] Kalron A, Tawil H, Peleg-Shani S, Vatine J-J (2018). Effect of telerehabilitation on mobility in people after hip surgery: a pilot feasibility study. Int J Rehabil Res.

[149] Bischoff-Ferrari HA, Dawson-Hughes B, Platz A (2010). Effect of high-dosage cholecalciferol and extended physiotherapy on complications after hip fracture: a randomized controlled trial. Arch Intern Med.

[150] Stemmle J, Marzel A, Chocano-Bedoya PO (2019). Effect of 800 IU Versus 2000 IU vitamin D3 with or without a simple home Exercise Program on Functional Recovery after hip fracture: a Randomized Controlled Trial. J Am Med Dir Assoc.

[151] Moseley AM, Sherrington C, Lord SR (2009). Mobility training after hip fracture: a randomised controlled trial. Age Ageing.

[152] Nightingale EJ, Sturnieks D, Sherrington C (2010). Impaired weight transfer persists at least four months after hip fracture and rehabilitation. Clin Rehabil.

[153] Woodward LM, Clemson L, Moseley AM (2014). Most functional outcomes are similar for men and women after hip fracture: a secondary analysis of the enhancing mobility after hip fracture trial. BMC Geriatr.

[154] Botella-Carretero JI, Iglesias B, Balsa JA (2008). Effects of oral nutritional supplements in normally nourished or mildly undernourished geriatric patients after surgery for hip fracture: a randomized clinical trial. JPEN J Parenter Enteral Nutr.

[155] Espaulella J, Guyer H, Diaz-Escriu F (2000). Nutritional supplementation of elderly hip fracture patients. A randomized, double-blind, placebo-controlled trial. Age Ageing.

[156] Neumann M, Friedmann J, Roy M-A, Jensen GL (2004). Provision of high-protein supplement for patients recovering from hip fracture. Nutrition.

[157] Hitz MF, Jensen J-EB, Eskildsen PC (2007). Bone mineral density and bone markers in patients with a recent low-energy fracture: effect of 1 y of treatment with calcium and vitamin D. Am J Clin Nutr.

[158] Leegwater NC, Nolte PA, de Korte N (2016). The efficacy of continuous-flow cryo and cyclic compression therapy after hip fracture surgery on postoperative pain: design of a prospective, open-label, parallel, multicenter, randomized controlled, clinical trial. BMC Musculoskelet Disord.

[159] Leegwater NC, Bloemers FW, de Korte N (2017). Postoperative continuous-flow cryocompression therapy in the acute recovery phase of hip fracture surgery-A randomized controlled clinical trial. Injury.

[160] Eriksson BI, Dahl OE, Lassen MR (2008). Partial factor IXa inhibition with TTP889 for prevention of venous thromboembolism: an exploratory study. J Thromb Haemost.

[161] Fuji T, Fujita S, Kawai Y (2014). Safety and efficacy of edoxaban in patients undergoing hip fracture surgery. Thromb Res.

[162] Lassen MR, Fisher W, Mouret P (2012). Semuloparin for prevention of venous thromboembolism after major orthopedic surgery: results from three randomized clinical trials, SAVE-HIP1, SAVE-HIP2 and SAVE-KNEE. J Thromb Haemost.

[163] Adachi JD, Lyles KW, Colón-Emeric CS (2011). Zoledronic acid results in better health-related quality of life following hip fracture: the HORIZON-Recurrent fracture trial. Osteoporos Int.

[164] Colón-Emeric CS, Caminis J, Suh TT (2004). The HORIZON recurrent fracture trial: design of a clinical trial in the prevention of subsequent fractures after low trauma hip fracture repair. Curr Med Res Opin.

[165] Prieto-Alhambra D, Judge A, Arden NK (2014). Fracture prevention in patients with cognitive impairment presenting with a hip fracture: secondary analysis of data from the HORIZON recurrent fracture trial. Osteoporos Int.

[166] Lyles KW, Colón-Emeric CS, Magaziner JS (2007). Zoledronic acid and clinical fractures and mortality after hip fracture. N Engl J Med.

[167] Eriksen EF, Lyles KW, Colón-Emeric CS (2009). Antifracture efficacy and reduction of mortality in relation to timing of the first dose of zoledronic acid after hip fracture. J Bone Miner Res.

[168] Colón-Emeric C, Nordsletten L, Olson S (2011). Association between timing of zoledronic acid infusion and hip fracture healing. Osteoporos Int.

[169] Boonen S, Orwoll E, Magaziner J (2011). Once-yearly zoledronic acid in older men compared with women with recent hip fracture. J Am Geriatr Soc.

[170] Magaziner JS, Orwig DL, Lyles KW (2014). Subgroup variations in bone mineral density response to zoledronic acid after hip fracture. J Bone Miner Res.

[171] Glendenning P, Chew GT, Seymour HM (2009). Serum 25-hydroxyvitamin D levels in vitamin D-insufficient hip fracture patients after supplementation with ergocalciferol and cholecalciferol. Bone.

[172] Glendenning P, Chew GT, Inderjeeth CA (2013). Calculated free and bioavailable vitamin D metabolite concentrations in vitamin D-deficient hip fracture patients after supplementation with cholecalciferol and ergocalciferol. Bone.

[173] Scheffers-Barnhoorn MN, van Eijk M, van Haastregt JCM (2019). Effects of the FIT-HIP intervention for fear of falling after hip fracture: a cluster-randomized controlled Trial in Geriatric Rehabilitation. J Am Med Dir Assoc.

[174] Di Monaco M, De Toma E, Gardin L (2015). A single postdischarge telephone call by an occupational therapist does not reduce the risk of falling in women after hip fracture: a randomized controlled trial. Eur J Phys Rehabil Med.

[175] Berger VW, Alperson SY. A general framework for the evaluation of clinical trial quality. Reviews on recent clinical trials. 2009 May 1;4(2):79-88. 10.2174/157488709788186021. 10.2174/157488709788186021 PMC2694951 19463104

[176] Olivo SA, Macedo LG, Gadotti IC, Fuentes J, Stanton T, Magee DJ. Scales to assess the quality of randomized controlled trials: a systematic review. Phys Ther. 2008;88:156–75. 10.2522/ptj.20070147. 10.2522/ptj.20070147 18073267

[177] Jadad AR, Moore RA, Carroll D, Jenkinson C, Reynolds DJ, Gavaghan DJ, et al. Assessing the quality of reports of randomized clinical trials: is blinding necessary? Control Clin Trials. 1996;17:1–12. 10.1016/0197-2456(95)00134-4. 10.1016/0197-2456(95)00134-4 8721797

