# Supplementary material for: Post-surgery interventions for hip fracture: a systematic review of randomized controlled trials
Source: BMC Musculoskelet Disord. 2023 May 25;24:417. doi: 10.1186/s12891-023-06512-9 (PMC10210378; doi:10.1186/s12891-023-06512-9)
Supplement: Supplementary file 1 — Supplementary Material 1 [file 12891_2023_6512_MOESM1_ESM.docx]

**Supplemental Table 1:** PRISMA 2009 checklist

| **Section/topic** | **#** | **Checklist item** | **Reported on page #** |
| --- | --- | --- | --- |
| Risk of bias across studies | 15 | Specify any assessment of risk of bias that may affect the cumulative evidence (e.g., publication bias, selective reporting within studies). | 4 |
| Additional analyses | 16 | Describe methods of additional analyses (e.g., sensitivity or subgroup analyses, meta-regression), if done, indicating which were pre-specified. | N/A |
| **RESULTS** | | |  |
| Study selection | 17 | Give numbers of studies screened, assessed for eligibility, and included in the review, with reasons for exclusions at each stage, ideally with a flow diagram. | 4  Fig. 1 |
| Study characteristics | 18 | For each study, present characteristics for which data were extracted (e.g., study size, PICOS, follow-up period) and provide the citations. | 4 |
| Risk of bias within studies | 19 | Present data on risk of bias of each study and, if available, any outcome level assessment (see item 12). | N/A |
| Results of individual studies | 20 | For all outcomes considered (benefits or harms), present, for each study: (a) simple summary data for each intervention group (b) effect estimates and confidence intervals, ideally with a forest plot. | Tables 1, Suppl table 3 |
| Synthesis of results | 21 | Present results of each meta-analysis done, including confidence intervals and measures of consistency. | N/A |
| Risk of bias across studies | 22 | Present results of any assessment of risk of bias across studies (see Item 15). | N/A |
| Additional analysis | 23 | Give results of additional analyses, if done (e.g., sensitivity or subgroup analyses, meta-regression [see Item 16]). | N/A |
| **DISCUSSION** | | |  |
| Summary of evidence | 24 | Summarize the main findings including the strength of evidence for each main outcome; consider their relevance to key groups (e.g., healthcare providers, users, and policy makers). | 9 |
| Limitations | 25 | Discuss limitations at study and outcome level (e.g., risk of bias), and at review-level (e.g., incomplete retrieval of identified research, reporting bias). | 10 |
| Conclusions | 26 | Provide a general interpretation of the results in the context of other evidence, and implications for future research. | 10 |
| **FUNDING** | | |  |
| Funding | 27 | Describe sources of funding for the systematic review and other support (e.g., supply of data); role of funders for the systematic review. | 1 |

**Supplemental Table 2**: Search strategy used for this study

| Database | Search Strategy | |
| --- | --- | --- |
| PubMed® | 1 | randomized controlled trial[Publication Type]  controlled clinical trial[Publication Type]  clinical trial[Publication Type]  clinical trials as topic[MeSH Terms]  clinical study[Publication Type]  multicenter study[Publication Type]  pragmatic clinical trial[Publication Type]  random*[Title/Abstract]  random allocation[MeSH Terms]  placebo[Title/Abstract] |
|  | 2 | Hip fractures[MeSH Terms] |
|  | 3 | femur*[Title/Abstract]  femoral*[Title/Abstract]  trochant*[Title/Abstract]  pertrochant*[Title/Abstract]  subtrochant*[Title/Abstract]  intracapsular*[Title/Abstract]  extracapsular*[Title/Abstract] |
|  | 4 | #2 OR #3 |
|  | 5 | #1 AND #4 |
|  |  | Search results: 20168 |
| Embase® | 1 | 'crossover procedure'/exp  'double-blind procedure'/exp  'randomized controlled trial'/exp  'single-blind procedure'/exp  (random* OR  factorial* OR crossover* OR cross NEXT/1 over* OR placebo* OR doubl* NEAR/1 blind* OR singl* NEAR/1 blind* OR assign* OR allocat* OR volunteer*):de,ab,ti |
|  | 2 | ‘hip fracture’/exp |
|  | 3 | ((hip* OR femur* OR femoral* OR trochant* OR pertrochant* OR intertrochant* OR subtrochant* OR intracapsular* OR extracapsular*) AND fracture*):de,ab,ti |
|  | 4 | #1 AND (#2 OR #3) |
|  |  | Search results: 12630 |
| Cochrane Library ® | 1 | ("randomized controlled trial" OR "randomised controlled trial" OR "controlled clinical trial" OR "randomized controlled trial as topic" OR "controlled clinical trial as topic" OR "doubl* blind" OR "singl* blind"):pt |
|  | 2 | (((hip* or femur* or femoral* or trochant* or pertrochant* or subtrochant* or intracapsular* or extracapsular*) NEAR/4 fracture*)):ti,ab,kw |
|  | 3 | [Hip Fractures] explode all trees |
|  | 4 | #2 OR #3 |
|  | 5 | #1 AND #4 |
|  |  | Search results: 1876 |
| ClinicalTrials.gov |  | "Hip Fractures" OR (((hip* or femur* or femoral* or trochant* or pertrochant* or subtrochant* or intracapsular* or extracapsular*) NEAR/4 fracture*)) |
|  |  | Search results: 592 |

**Supplemental Table 3**: Characteristics of included RCTs

| **Study (Objective)** | **N; mean age (years)** | **Intervention (design; experimental and control; setting)** | | | **Primary Outcomes Measures** | **Secondary Outcomes Measures** | **Findings and Comments** |
| --- | --- | --- | --- | --- | --- | --- | --- |
|  |  | **Design** | **Experimental (*setting*)** | **Control (*setting*)** |  |  |  |
| **Rehabilitation** | | | | | | | |
| Binder et al.[33], Host et al. [34]  (To determine whether extended outpatient rehabilitation that includes progressive resistance training improves physical function and reduces disability compared with low-intensity home exercise among physically frail elderly patients with hip fracture.) | 90; 80.5 | 1:1 | Physical therapy (*indoor exercise facility*); 2 approximately 3-month-long phases of exercise training (2nd phase include progressive resistance training), thrice per week. | Home exercise (*home and indoor exercise facility*); prescribed a home exercise program, attended 1-hour monthly group training session, and asked to perform exercises at home thrice a week, for a period of 2 consecutive 3-mth interval. | Total scores on modified Physical Performance Test (PPT), Functional Status Questionnaire, physical function subscale (FSQ), activities of daily living skills | Skeletal muscle strength, gait, balance, quality of life (QoL) and body composition | ↑ physical function, QoL and strength  ↓ disability  After 6 months of extended outpatient rehabilitation in a supervised exercise setting that includes progressive training, compared with low-intensity home exercise. |
| Bischoff-Ferrari et al. [149], Stemmle et al. [150]  (To determine the additive benefit of extended physiotherapy (PT) and cholecalciferol  therapy, 2000 IU/d, on the rate of falls and hospital readmissions in the first 12 months after acute hip fracture.) | 173; 84 | 1:1:1:1 | 1. 2000 IU/day cholecalciferol + standard PT (*inpatient and home*); 2000 IU/day cholecalciferol taken in the morning and at night, and 30min/day of standard PT during acute care with no home program.  2. 2000 IU/day cholecalciferol + extended PT (*inpatient and home*); 2000 IU/day cholecalciferol taken in the morning and at night, and standard PT plus additional 30min of home program instruction each day during acute care. After discharge, home program to be followed for 30min/day.  3. 800 IU/day cholecalciferol + extended PT (*inpatient and home*); 800 IU/day cholecalciferol taken in the morning and at night, plus 1200 IU/day placebo taken in the morning. PT as described in group 2. | 800 IU/day cholecalciferol + standard PT (*inpatient and home*); 800 IU/day cholecalciferol taken in the morning and at night. 30min/day of standard PT during acute care with no home program. | Rate of falls | Rate of hospital admissions (12-month follow-up) | ↓ falls with extended PT  ↓ hospital readmission with supplementation of cholecalciferol, 2000 IU/d  ↑ functional recovery with extended PT and 800IU vitamin D3, compared with no home exercise or 2000IU vitamin D3.  ↔ subjective physical functioning with all interventions |
| Braid et al. [56]  (To determine whether 6 weeks of supplementary  cyclic electrical stimulation applied to the quadriceps of the fractured leg increases leg extensor power and decreases disability in elderly patients rehabilitating  after proximal femoral fracture.) | 26; 80.5 | 1:1 | Electrical Stimulation (ES) Group (*inpatient and home*); 6 weeks of supplementary electrical stimulation of quadriceps (5 days/week as inpatient and twice weekly once discharged, 18 minutes per session), plus standard inpatient physiotherapy. | Control Group (*inpatient*); standard inpatient physiotherapy | Change in leg extensor power measured (Nottingham Power Rig) | 1. Functional mobility (Elderly Mobility Scale)  2. Disability (Barthel Index)  3. Health status (Nottingham Health Profile) | ↔ leg extensor power and disability in frail elderly, possibly due to a comparatively lower tolerance of stimulation intensity, increased electrical impedance around the thigh or post-operative tissue oedema etc. |
| Cameron et al. [60]  (To determine if accelerated  rehabilitation would be associated with improved level  of physical independence, a reduced length of hospital stay, improved accommodation status after discharge  from hospital, and no additional burden on carers.) | 252; 85 | 1:1:1 | Accelerated rehabilitation (*inpatient, nursing home, outpatient and home*); early assessment of rehabilitation goals, early commencement (usually within 24 hours of surgery), greater emphasis on retraining physical independence, closer family caregiver contact and more detailed discharge planning. Rehabilitation continues post-discharge till they have reached their pre-morbid level of functioning or plateau at a lower level of function. | Conventional care (*inpatient*); standard treatment provided at the study hospital, usually commencing after 24 hours of surgery. Rehabilitation continues post-discharge till they have reached their pre-morbid level of functioning or plateau at a lower level of function. | 1. Physical independence  2. Length of hospital stay  3. Improved accommodation status after discharge from hospital  4. Caregiver burden | Nil | ↓ length of hospital stay  modest short-term improvement in physical independence and accommodation status. |
| Elboim-Gabyzon et al. [19]  (To examine the effect of incorporating  Transcutaneous electrical nerve stimulation (TENS) treatment on pain intensity, and mobility, with standard rehabilitation care during the acute post-operative phase following Gamma-nail surgical fixation of extracapsular hip  fractures.) | 41; 79.2 | 1:1 | Active TENS (*inpatient*); standard interdisciplinary postoperative treatment; electrical stimulation (bi-phasic symmetric waveform at a continuous frequency of 100Hz and phase duration of 200µsec, intensity was gradually adjusted up to a strong but comfortable 1 level, as reported by subject) was administered each morning for 30 minutes for a total of 5 treatments. | Sham TENS (*inpatient*); standard interdisciplinary postoperative treatment; clinical stimulator TENS device was used and turned on so that subject would see a green light, but no current was delivered. | Pain intensity at rest, at night and during ambulation | Sit-to-stand timing | ↓ pain intensity during walking  ↑ functional gait recovery  Following Gamma nail surgical fixation of extracapsular hip fracture. |
| Faldini et al. [57]  (To determine the effect of pulsed electro-magnetic fields (PEMF)  on time to healing, onset of osteonecrosis of the femoral head  and pain in patients with intracapsular femoral neck fracture.) | 77; 69 | 6:7 | Intervention (*inpatient and home*); patients given a stimulator and instructed to use it for at least 8 h/day for 90 days. Active stimulator generated pulsed electromagnetic field of 75 Hz, 1.3 m/s duration of impulse, 2mTesla peak value of magnetic field. | Placebo (*inpatient and home*); patients given a placebo stimulator and instructed to use it for at least 8 h/day for 90 days. | Facture healing, onset of osteonecrosis, pain | Patient compliance | ↑ fracture healing and ↓ pain in active compliant patients compared with placebo group  ↔ onset of osteonecrosis |
| Gorodetskyi et al. [20]  (To determine if a non-invasive, interactive neurostimulation  device could reduce pain and the time required to achieve functional independence  in older patients recovering from stabilization of fractures to the trochanteric area of the femur.) | 60; 71.2 | 1:1 | Active non-invasive interactive neurostimulation (NIN) therapy group (*inpatient*); standard interdisciplinary postoperative care plus non-invasive neurostimulation  therapy within 24 hours of surgery, for 10 days. Daily treatment for a total of 20 to 30 minutes combined, at the three sites close to the surgical incision, as well as the corresponding areas on the contralateral side using the InterX 5000, high peak amplitude averaging 17 volts on the skin with a low current of about 6 mA. | Sham group (*inpatient*); standard interdisciplinary postoperative care plus treatment using a sham device for the same duration as intervention group. | 1. Visual analogue scale for pain  2. Brief pain inventory and Keterolac for post-operative control of pain  3. An overall assessment of progress by the surgeon using a Likert scale. | Nil | ↑ recovery in range of movement and ↓ pain by the 4^th^ post-operative day.  A similar improvement in pain was delayed until the 10^th^ post-operative day in the sham group, suggesting that non-invasive neurostmulation therapy could play a valuable role in reducing length of hospital stay. |
| Hagsten et al. [58, 59]  (To study the  effects of an early, individualized, post-operative Occupational therapy (OT) training program.) | 100; 80 | 1:1 | OT group (*inpatient and home*); same postoperative care at the ward during the first 3−4 days after surgery. After the first self-assessment, followed by individualized training by an occupational therapist for 45–60 min each weekday morning. During the stay, occupational therapist and the patient visited the patient’s home to determine how to prepare and adapt the home environment for maximum independence. | Control group (*inpatient*); conventional care. | 1. Activities of daily living (ADL) and instrumental activities of daily living (IADL) ability assessed by occupational therapist  2. Self-reported Disability Rating Index (DRI)  3. Self-reported health related quality of life (HRQL) | Possible fear of performing ADL and pain level during performance. | ↑ ADL and IADL abilities – dressing, toilet visits and bathing/hygiene  ↔ HRQL  Individualized occupational training increases the possibility of patients returning to their homes with greater independence and autonomy. |
| Kalron et al. [148]  (To evaluate the effects of  telerehabilitation on mobility in people following hip surgery.) | 32; 66.5 | 1:1 | Telerehabilitation (*Home*); provided with video clips of common rehabilitation exercises focusing on the lower limbs, to be performed 3 sessions/week for 6 consecutive weeks, 40-50 minutes/session. | Control (*Home*); received an exercise booklet with exercises similar to those of the telerehabilitation group, to be performed 3 sessions/week for 6 consecutive weeks, 40-50 minutes/session. | 1. Timed Up and Go test  2. 2-min walk test  3. 10-m walk test  4. Sit to stand test  5. Walking speed  6. Mean step length  7. Compliance | Nil | ↑ mobility functions  ↑ compliance rates |
| Kimmel et al. [22]  (To investigate the effects of intensive acute hospital  physiotherapy for patients with isolated hip fractures.) | 92; 81.3 | 1:1 | Intensive physiotherapy (*Inpatient*); usual care plus 2 additional daily sessions by an allied health assistant and a physiotherapist. | Usual care (*Inpatient*); daily physiotherapy according to usual practice, 7 days per week. | Modified Iowa Level of Assistance (mILOA) score, post-operative Day 5. | 1. Timed Up and Go (TUG) test  2. Acute hospital length of stay (LOS), inpatient rehabilitation LOS and combined hospital LOS  3. Inpatient complications  4. Re-admissions  5. Time to physical readiness for discharge  6. Discharge destination  7. Pain scores before and after physiotherapy | ↑ (better) mILOA score at Day 5  ↔ functional mobility at Day 5, pain levels or opioid pain relief requirements  ↓ hospital LOS by more than 10 days without increasing the rates of complications or readmissions |
| Kronborg et al. [23]  (To examine whether 1) acute  in-hospital physiotherapy (PT) with addition of progressive knee-extension strength training (ST) of the fractured limb is more effective in reducing the knee-extension strength deficit at follow-up compared to PT without additional ST and, 2) if patients following the ST intervention present larger improvements in  physical function compared to PT only. | 90; 79.5 | 1:1 | ST (*Inpatient*); routine PT treatment and additional daily individual progressive knee-extension ST, 3 x 10 repetitions, of fractured limb using ankle weight cuffs conducted by ward physical therapist during hospital stay, over a mean period of 6 days. | PT (*Inpatient* ); routine PT treatment during hospital stay, over a mean period of 6 days | Change in maximal isometric knee-extension strength in fractured limb in percentage of the non-fractured limb from inclusion to post-operative day 10 or discharge | Time up and go test, gait spend, fear of falling, basic mobility capacity, pain | ↔ strength, given that only 5 exercise sessions on average were completed, but may still be clinically important for fragile patients in the acute phase, where the ability to participate in functional exercise is compromised. |
| Kuisma et al. [42]  (To compare ambulation outcomes between home and institutional  rehabilitation of patients with hip fracture.) | 81; 75 | 1:1 | Study (*Home*); patients discharged directly home from the acute hospital and visited by a physiotherapist an average of 4.6 times | Control (*rehabilitation hospital*); patients discharged to a rehabilitation centre for further treatment lasting on average 36.2 days and receive physiotherapy daily | Ambulatory ability | Nil | ↑ ambulation ability (community ambulation and walking on flat surface) at discharge and completion of rehabilitation, indicating that domiciliary physiotherapy (with home support) is an effective alternative to institutional care. |
| Lahtinen et al. [32]  (To examine effects of physical and geriatric rehabilitation on institutionalisation and mortality  after hip fracture.) | 538; 78.1 | 1:1:1 | 1. Geriatric rehabilitation (*inpatient geriatric ward*); geriatric department chaired by a geriatrician and was focused on both the physical training as well as the associated geriatric problems.  2. Physical rehabilitation (*inpatient rehabilitation unit*); rehabilitation unit of a private hospital, chaired and run by a neurologist with special qualification in rehabilitation of disabilities in locomotor function. Duration of rehabilitation was a maximum of 3 weeks. | Control group (*inpatient*); routine basic level of rehabilitation in the local health centre hospitals. | 1. Social status  2. Residential status  3. Walking ability  4. Use of walking aids  5. Pain in the hip  6. Activities of daily living (ADL)  7. Mortality | Nil | ↓ mortality at 4 and 12 months for patients who underwent specialised physical rehabilitation  ↑ number of patients in both intervention groups were at home at 4 months  ↔ ADL functions or walking ability at either 4 months or 12 months in all 3 groups |
| Lamb et al. [55]  (To study the feasibility and effect of neuromuscular  stimulation on recovery of mobility after surgical fixation for hip fracture.) | 24; 83.7 | 1:1 | Patterned neuromuscular stimulation (PNMS) (*Home*); stimulators were worn for 3 hours per day for 6 weeks (total 84 hours of PNMS), output was a constant, balanced, asymmetric biphasic pulsed current with a pulse width of 300µs and a maximum output intensity of 100mA. Both groups received routine physical therapy as inpatients. | Placebo stimulation (*Home*); single 300µs pulse delivered every 3 minutes, which produced a strong sensory stimulus but negligible muscle activation. | Recovery of walking speed and ability, postural stability, lower-limb muscle power, and pain at 7 and 13 weeks after surgery | Nil | ↔ walking speed between both groups after 6 weeks of stimulation, but after stimulation was ceased, ↑ in recovery of walking speed in PNMS group.  ↑ postural stability after 7 weeks of stimulation  ↔ leg extensor power during or after stimulation period, and pain scores  It is possible to speed the early recovery of mobility, but it is premature to suggest that PNMS is the optimal method and should be adopted for routine clinical use. |
| Latham et al. [43], Chang et al. [44]  (To determine whether a home exercise program with minimal contact with a physical therapist improved function after formal hip fracture rehabilitation ended.) | 232; 78.1 | 1:1 | Intervention Exercise Program (*Home*); home exercise program 3 times per week for 6 months, taught in 3 home visits conducted by a physical therapist, including monthly telephone calls and a DVD version of the program. | Attention Control Group (*Home*); registered dieticians provided nutritional education for cardiovascular health based on the Dietary Guidelines for Americans during a single home visit, followed by a series of telephone calls and mailings. | 1. Physical function at 6 months after randomization.  2. Basic mobility and daily activity functioning and perceived self-efficacy. | Lower extremity isometric muscle strength, balance, self-efficacy, adverse events and exercise adherence | Modest improvement in physical function at 6 months.  ↓ self-efficacy over time in the control group, while in the intervention group, it slightly decreased from baseline to 6 month but increased from 6 month to 9 month. Self-efficacy may play a partial mediating role in maintaining the effect of the intervention. |
| Magaziner et al. [54]  (To compare a multicomponent home-based physical therapy intervention  (training) with an active control on ability to walk in the community.) | 210; 80.8 | 1:1 | 1. Training group (*Home*); 16 weeks of 60-minute in-home visits provided by physical therapists, focused on lower extremity strength, endurance, balance, and function.  2. Active control group (*Home*); 16 weeks of 60-minute in-home visits provided by physical therapists, which included 22 seated active range-of-motion exercises and sensory-level TENS unit application to lower extremity muscle groups.  Both groups received 2000 IU of vitamin D3, 600mg of calcium and a multivitamin daily for 40 weeks, and nutritional counselling at baseline by a registered dietician. | Nil | Community ambulation (16 weeks after randomization) as assessed by the 6-minute walk test (walking 300m or more in 6 minutes) | 1. Endurance  2. Dynamic balance  3. Isometric quadriceps strength on nonfractured side  4. Gait speed  5. Physical performance  6. Lower extremity function  7. Increase of 50m or more on 6-minute walk test | ↔ community ambulation after 16 weeks, possibly due to insufficient dose of exercise received by some participants as well as lower adherence rate in the training group. |
| Mangione et al. [49]  (To examine the effectiveness of a short term leg strengthening exercise program  compared to attentional control on improving strength, walking abilities, and function one year  after hip fracture.) | 26; 80.8 | 1:1 | Exercise intervention (*Home*); Twice a week (30-40 minutes each session) for 10 weeks for 20 total sessions. Consists of strengthening exercises for the hip extensors, hip abductors, knee extensors, and ankle plantar flexors bilaterally. | Control (*Home*); conventional transcutaneous electrical stimulation (TENS) for 7 minutes for a total of 21 minutes each session. | 1. Isometric force production of lower extremity muscles  2. Usual and fast gait speed  3. Six-minute walk (6-MW) distance  4. Modified physical performance test (mPPT)  5. SF-36 physical function | Nil | ↑ force production, gait speed and endurance, and physical performance one year after hip fracture (progressive resistance training for the leg muscles that began 6 months after hip fracture).  Such an exercise program can be translated into the home setting. |
| Mangione et al. [52]  (To determine the effects and feasibility of a home exercise program of moderate- or high-intensity exercise, and the relationship of depression and physical recovery.) | 33; 78.5 | 1:1:1 | Total of 20 visits in subjects’ homes by physiotherapist, each session was 30-40 minutes, 2 times per week for the first 2 months and 1 time per week for the third month  1. Aerobic training group; 2-3 minutes of warm-up active ROM exercise followed by walking or upper- and lower-extremity active ROM exercises for 20 minutes to keep the heart rate elevated.  2. Resistance training group; performed using a portable progressive-resistive exercise machine and body weight. | Control group; received biweekly mailings from health institution on a variety of non-exercise topics and asked not to begin any new exercise programs until the study was completed. | 1. Maximum voluntary isometric lower-extremity force  2. 6-minute walk distance  3. Free gait speed  4. Self-reported physical function | 1. Feasibility of such an exercise program  2. Relationship of depression and physical recovery | ↑ isometric force of the involved limb in both intervention groups  High- and moderate-intensity exercise can be performed at home for elderly people with a hip fracture, did not appear to produce adverse events and adherence to training was excellent.  Depression may play a role in the level of improvement attained. |
| Martín-Martín et al. [31]  (To explore whether an occupational therapy intervention combined with physiotherapy  rehabilitation improved hip fracture patient outcomes regarding emotional distress, fatigue, independence  and function.) | 122; 82 | 1:1 | Combined Treatment group (CT) (*inpatient*); individual occupational therapy was added to the SC program following occupational therapy practice guidelines, to provide strategies for autonomy recovery as early as possible. | Standard care group (SC) (*inpatient*); standard medical and physical therapy treatment without any  occupational therapy intervention. | 1. Patients’ emotional distress (GHQ-28)  2. Perceived fatigue (the first item of the BASDAI)  3. Level of independence (Modified Barthel Index)  4. Function  (Harris Hip Score), up to 6 months after intervention. | Nil | ↓ emotional distress from the start of treatment  ↓ fatigue at 6 months  Slight ↑ in function and independence levels |
| Mendelsohn et al. [25]  (To evaluate the effect of an upper-body exercise  program on cardiorespiratory fitness in older adults with hip  fracture during inpatient rehabilitation. | 20; 81.3 (Training group) | 1:1 | Training Group (*inpatient*); 3 sessions a week for 4 weeks. Each session included a warm-up period (5min) at  no resistance (0W), an endurance phase (20min), and a cooldown  period (5min) at 0W. | Control Group (*inpatient*); standard rehabilitation care program. | Aerobic capacity (VO_2_peak) | 1. Timed Up & Go (TUG) test  2. Berg Balance Scale (BBS)  3. FIM instrument  4. two-minute  walk test (2MWT)  5. ten-minute walk test  (10MWT) | ↑ in VO_2_peak and all physical function measures, suggesting that upper body aerobic training may enhance rehabilitation care. |
| Mitchell et al. [24]  (To determine whether systematic progressive high-intensity  quadriceps training increases leg extensor power and reduces disability in patients rehabilitating after proximal femoral fracture.) | 80; 80.05 | 1:1 | Quadriceps training group (*inpatient*); standard care plus twice weekly progressive training of their quadriceps in both the recently fractured and the unaffected leg, for 12 sessions. | Control group (*inpatient*); physiotherapy (5 days per week) for approximately 20 minutes per day. | 1. Leg extensor power  2. Functional mobility  3. Disability  4. Quality of life | Nil | ↑ leg extensor power  ↓ disability |
| Monticone et al. [26]  (To evaluate the efficacy of a rehabilitation programme including balance task-specific training in improving physical function, pain, activities of daily living (ADL), balance and quality of life in subjects  after a hip fracture.) | 52; 77.5 | 1:1 | Experimental group (*inpatient*); balance task-specific exercises while standing with open and closed eyes, and additional exercises such as moving from a sitting to a standing position, ascending/descending stairs and climbing obstacles. Ergonomic advice was also provided to facilitate the modification of their daily living activities. | Control group (*inpatient*); open kinetic chain exercises in the supine position on the couch, with ergonomic advice to facilitate the modification of their daily living activities. | Physical function using the self-reported Western Ontario and McMaster Universities Osteoarthritis Index (WOMAC) | 1. Pain  2. Balance  3. Activities of Daily Living (ADLs)  4. Quality of life | ↑ in all outcomes, significantly for physical function. |
| Moseley et al. [151], Woodward et al. [153], Nightingale et al. [152]  (To compare the effects of two different exercise programmes after hip fracture.) | 160; 84 | 1:1 | Higher dose weight-bearing exercise (*inpatient and home*); inpatient program of weight-bearing exercise twice daily for a total of 60 min per day for 16 weeks, progressed by reducing support and increasing height of exercise tools and number of repetitions. Followed by home visits and a structured home exercise program. | Lower dose weight-bearing exercise (*inpatient and home*); inpatient program of five exercises in sitting or lying plus a small amount of walking using parallel bars or walking aids for a total of 30 min each day for 4 weeks, progressed by increasing repetitions and resistance. Followed by weekly home visits and a structured home exercise program. | 1. Knee extensor strength in the fractured leg  2. Walking speed | Functional abilities, balance abilities, pain, fear of falling, quality of life, length of stay in hospital, residential status and community service utilization after discharge, adverse events and adherence with the treatment programs. | ↔ primary outcomes  ↑ functional abilities, balance abilities, falls efficacy and quality of life, with ↓ pain, for people with cognitive impairment assigned to intervention group.  ↑ choice stepping reaction time but performance remained at around 4 months after fracture. |
| Oldmeadow et al. [21]  (To investigate the effect of early ambulation (EA) after hip fracture surgery on patient and hospital outcomes.) | 60; 79.4 | 1:1 | Early ambulation group (*inpatient*); routine postoperative medical and nursing clinical care, commence walking with a physiotherapist (s) as soon as possible on postoperative day 1 or 2. | Delayed ambulation group (*inpatient*); routine postoperative medical and nursing clinical care, did not commence walking until postoperative day 3 or 4. | Functional level,  represented by the distance walked and the level of assistance required to transfer from supine to sit, sit to stand and to negotiate one step, on day 7 post-surgery. | Discharge destination and length of stay in the acute care. | ↑ functional recovery  ↓ length of stay if patients were clinically stable and commenced walking within 48 hours. |
| Orwig et al. [50], Yu-Yahiro et al. [51]  (To determine  whether a yearlong home-based exercise program initiated following usual care could be administered to older patients with hip fracture and improve outcomes.) | 180; 82.4 | 1:1 | In-Home Exercise (*Home*); strength training and aerobics components, participants were expected to perform aerobic activity ≥3 days per week and strength training ≥2 days per week for 30 minutes, either supervised or independently, for a period of 12 months. | Usual Care (*Outpatient / Home*); physician-prescribed post-fracture standard of care for patients with hip fracture. | Bone mineral density at the contralateral femur. | 1. Total lean body mass and fat mass  2. Hours spent exercising and kilocalories expended in a week  3. 6-minute walk test  4. Lower Extremity Gain Scale  5. Measure of gait and global balance  6. Grip strength  7. Lower Extremity Physical Activities of Daily Living  8. Geriatric Depression Scale  9. Health-related QoL  10. Feasibility and challenges of  administering an intensive home-based exercise program in this population of older adults | ↑ activity level but ↔ improvement in other targeted outcomes.  Rate of participation and enjoyment of the program was high. |
| Peterson et al. [41]  (To determine the outcome of implementing an intensive rehabilitation program for people who had sustained a hip fracture.) | 70; 78.5 | 19;16 | Treatment (*Home*); patients scheduled to attend physical therapy sessions biweekly for 8 weeks (60 mins per session), consisting of circuit training concomitant with an individualised balance and gait training programme. | Control (*Inpatient and home*); no intervention. | Standardised measures of strength, balance, gait and functional performance | Demographic data, standardised questionnaires on e.g. depression, life events, social network | Study was not able to show an effect of intervention to improve rehabilitation after hip fracture, due to low compliance. |
| Pol et al. [61, 62]  (To test the effects of an intervention involving sensor monitoring-informed occupational therapy on top of a  Cognitive behavioural treatment (CBT)-based coaching therapy on daily functioning in older patients after hip fracture.) | 240; 83.8 | 1:1:1 | 1. Cognitive behavioural treatment (CBT)-based occupational therapy (*Inpatient to home*); received coaching aimed at the recovery in daily functioning based on principles of CBT as well as usual care, followed by 4 home visits by an occupational therapist in the first 4 weeks after discharge, followed by 4 telephone consultations.  2. CBT-based occupational therapy with sensor monitoring (*Inpatient to home*); same as above as well as sensor monitoring which consists of a wearable physical activity monitor, motion sensors placed in the main spaces in the patients’ house and a gateway. Followed by 4 home visits by an occupational therapist in the first 4 weeks after discharge, followed by 4 telephone consultations. | Care as usual (*Inpatient for most, carried on to home for a minority*) | Patient-reported daily functioning at 6 months after the start of the rehabilitation | 1. Performance satisfaction in daily functioning at six month  2. Physical functioning  3. Timed up and Go  4. ADL 15 index score  5. Level of sense of safety  6. Fear of falling  7. Falls efficacy scale  8. Health related quality of life | ↑ patient reported daily functioning at 6 months for intervention of sensor monitoring-informed OT coaching. |
| Portegijs et al. [35], [143], Pakkala et al. [144], Edgren et al. [36]  (To study the effects of resistance training on muscle strength parameters, mobility, and balance.) | 46; 74 | 1:1 | Training Group (*senior gym*); 12-week individually  tailored training program that was organized twice a week (1–1.5h) in a senior gym and supervised by an experienced physiotherapist. | Control Group (*Home*); no intervention, encouraged to continue their lives as usual and maintain their pre-study level of physical activity during the 12-week trial. | 1. Isometric knee extension torque (KET) and leg extension power (LEP) measured in the weaker and stronger leg and the asymmetric deficit  2. 10-m walking speed, dynamic balance test, and self-reported outdoor mobility. | Sense of coherence | ↑ muscle strength and power  ↔ sense of coherence  ↓ self-reported difficulties, especially activities of daily living (ADL), even several years after a hip fracture. |
| Resnick et al. [147]  (To test the impact of a self-efficacy based intervention, the Exercise Plus Program, and the different components of the intervention, on self-efficacy, outcome expectations, and exercise behavior among older women post–hip fracture.) | 208; 81.0 | 1:1:1:1 | 1. Exercise Plus (*Home*); exercise sessions with an exercise trainer, sessions incorporated aerobic, strengthening and stretching exercises. Participants were told to perform aerobic activity at least 3 days per week and strength training 2 days per week for 30min. Includes education about the benefits of exercise post-hip fracture, verbal encouragement through goal setting and positive reinforcement etc.  2. Exercise Only (*Home*); exercise sessions with trainer but not exposed to the plus components of the intervention and not provided with any education about exercise, verbal feedback, interventions to decrease unpleasant sensations or encouragement to exercise  3. Plus Only (*Home*); sessions with trainer focused only on the plus components (i.e., education about exercise, verbal encouragement, removal of unpleasant sensations, and cueing) | Routine Care (*inpatient and home*); inpatient physical and occupational therapy  based on the functional needs of the individual and in  most cases a single home therapy evaluation for safety | 1. Self-efficacy for walking/exercise scale  2. Exercise behavior based on the Yale Physical Activity Survey and the Step Activity Monitor (SAM) | Nil | ↑ reported time in exercise activities  ↔ self-efficacy and outcome expectations |
| Salpakoski et al. [45], Edgren et al. [8], Turunen et al. [46], ss et al. [47], Portegijs et al. [48]  (To investigate whether a home-based rehabilitation program for community-dwelling older people with recent hip fracture is more effective than standard care in improving mobility recovery and reducing disability.) | 81; 80 | 1:1 | Intervention (*Home*); standard care after discharge home and individually tailored 12-month physical activity and rehabilitation intervention implemented in the participants’ homes. Included 5 to 6 home visits by a physiotherapist. | Control (*Home*); standard care after discharge home included a written home exercise program from the hospital or health care centre, a referral to physiotherapy was occasionally prescribed. | 1. Asymmetrical leg extension power deficit  2. Lower extremity performance and functional balance  3. Mobility disability | Self-reported or performance-based mobility | ↑ mobility recovery, with more apparent benefits in balance or physical performance in the long-term rather than seen short-term. |
| Sherrington et al. [27]  (To assess the effects of weight-bearing and non-weight-bearing exercise on strength, balance,  gait and functional performance among older inpatients following hip fracture.) | 80; 81 | 1:1 | 1. Weight-bearing exercise (*Inpatient*); carried out exercises in weight-bearing positions for a period of 2 weeks.  2. Non weight-bearing exercise (*Inpatient*); carried out exercises in a non-weight-bearing (supine) position for a period of 2 weeks. | Nil | Standardised measures of strength, balance, gait and functional performance | Self-report on balance, health, fall risk and quality of sleep | ↔ strength, balance, gait and functional performance in both groups. |
| Sherrington et al. [145]  (To compare the effects of weight-bearing and non–weight-bearing home exercise programs and a control program on physical ability (strength, balance, gait, functional  performance) in older people who have had a hip fracture.) | 120; 79 | 1:1:1 | 1. Weight-bearing home exercise (*Home*); prescribed exercises in a weight-bearing position, with follow-up assessment and revision done at 1-month and 4-month.  2. Non-weight-bearing exercise (*Home*); prescribed exercises in a non-weight-bearing position, with follow-up assessment and revision done at 1-month and 4-month. | Control; no intervention | 1. Strength  2. Balance  3. Gait  4. Functional performance | 1. Self-reported fall risk  2. Balance  3. Health  4. Quality of night-time sleep  5. Pain  6. Mobility and activity level  7. Impairments relating to activities of daily living (ADLs) | ↑ balance and functional ability in weight-bearing exercise group  ↔ strength and gait in weight-bearing exercise group, but more people in this group were able to walk unaided at the 4-month retest. |
| Sherrington et al. [53]  (To determine the effect of a home exercise program  on strength, postural control, and mobility following hip  fracture.) | 42; 79 | 1:1 | Treatment (*Home or institutional care*); weight-bearing exercise, at least once a day, where number of repetitions initially prescribed ranged from 5 to 50, for a period of 1 month, carried out at home. | Control (*Home or institutional care*); no intervention. | 1. Quadriceps strength  2. Postural sway  3. Functional reach  4. Weight-bearing ability  5. Walking velocity  6. Self-rated fall risk | Nil | ↑ lower limb strength and walking velocity |
| Singh et al. [39]  (To test a novel, evidence-based treatment strategy to improve long-term outcomes after hip fracture by targeting  sarcopenia with 12 months of high-intensity progressive resistance training.) | 124; 79 | 1:1 | HIPFIT (O*utpatient clinic*); 2 days per week for 12 months of geriatrician-supervised high-intensity progressive resistance training in the outpatient clinic. Monthly phone call and monthly residential visit by their trainer. | Usual Care (O*utpatient clinic*); includes orthogeriatric care, rehabilitation service, other medical and allied health consultation as required, and physiotherapy. | 1. Date and cause of death (if relevant)  2. Nursing home residence at time of acute and rehabilitation hospital discharge  3. Activities of daily living (ADL) and instrumental activities of daily living (iADL) | Nil | ↓ mortality, nursing home use and assistive device use  ↑ ADL independence in toileting and transferring |
| Suwanpasu et al. [40]  (To determine the effects of a physical activity enhancing program (PEP) on the level of physical activity of elderly patients after surgical treatment of hip fracture.) | 46; 75.2 | 1:1 | Treatment (*Outpatient clinic*); four phases of physical training and efficacy-based intervention comprising assessment, preparation, practicing, and evaluation phases with face-to-face contact and five telephone calls for 7 weeks post-surgery. | Control (*Home*); usual standard care. | Standardised measure of physical activity performance | Demographic data, cognitive condition, pre-fracture physical activity | ↑ physical activity |
| Sylliaas et al. [37, 38]  (To assess the effect of a 3-month strength-training programme on functional performance  and self-rated health in a group of home-dwelling older hip fracture patients.) | 150; 82.5 | 2:1 | Intervention (*Outpatient clinic*); 12 weeks of exercises conducted by a physiotherapist using a combination of group and individual sessions, includes exercises on stationary bicycle or treadmill based on participants’ one-repetition maximum (1-RM) voluntary strength.  Subsequently, a new randomisation of participants to continue or discontinue with strength exercise. Intervention is an extended 12 weeks of exercise sessions once a week and to complete a home training programme once a week, exercises were conducted by a physiotherapist using a combination of group and individual sessions. | Control (*Outpatient clinic*); usual lifestyle (12 weeks after hip fracture), no restrictions placed on their exercise activities. | 1. Berg Balance Scale (BBS) score at 24 weeks after fracture (after 12 weeks of training)  2. Berg Balance Scale (BBS) score at 36 weeks after fracture (after 24 weeks of training) | 1. Strength – sit-to-stand test and maximum step high test  2. Mobility – Timed Up-and-Go test, maximum gait speed and 6-min walk test  3. Activities of Daily Living  4. Self-rated health  Measured after 12 weeks and 24 weeks training period | ↑ balance, strength, gait distance and functional performance and  ↔ self-rated health after first 12 weeks of exercises.  ↔ balance after 12 weeks of extended exercises but may improve strength, endurance, self-reported activities of daily living, and self-perceived health. |
| Taraldsen et al. [146]  (To evaluate the clinical effectiveness and cost-effectiveness of a home-based exercise program delivered four months following hip-fracture surgery.) | 143; 83.4 | 1:1 | Intervention (*Home*); home-based programme consisted of five weight-bearing exercises, starting 4 months post-surgery, supervised by a physiotherapist twice weekly for 10 weeks, each session lasting approximately 45 minutes. | Control (*Inpatient*); treatment as usual, which included a variety of different approaches, from no follow-up at all to quite extensive interdisciplinary rehabilitation in their homes or in an institution. | Gait speed | 1. Gait variables  2. Physical activity  3. Mobility  4. Basic and instrumental ADL  5. Cognitive function  6. Health-related quality of life | ↑ gait speed (immediate and lasting (6 months after intervention))  ↔ self-reported function |
| Uy et al. [30]  (To determine the effectiveness of interdisciplinary  rehabilitation for women with hip fracture who were residents  of nursing homes.) | 11; 81.5 | 1:2 | Intervention Group (*Inpatient*); inpatient interdisciplinary rehabilitation program provided using the system of accelerated rehabilitation. | Control Group; discharge back to the nursing home soon after hip surgery. | 1. Barthel index  2. Timed 2.44-m walk | Nil | ↔ Barthel index and gait velocity  (data suggested some participants benefited but numbers of participants are too small to draw definitive conclusions) |
| van Ooijen et al. [28, 29]  (To determine if both conventional treadmill training and C-Mill gait  adaptability treadmill training result in better outcomes related to walking ability than usual care due to the enhanced training intensity.) | 70; 83.4 | 1:1:1 | 1. Adaptability treadmill (AT) training (*Inpatient*); 15 of UPT sessions replaced by adaptability treadmill training, with visual context projection and without bodyweight support other than the handrail.  2. Conventional treadmill (*Inpatient*); 15 of the UPT sessions replaced by treadmill walking without projection of visual context and used no body weight support other than the handrail. | Usual Physical Therapy (UPT) (*Inpatient*); 6 weeks of inpatient training – 30 sessions of conventional physical therapy. | 1. Walking ability – e.g., Performance Oriented Mobility Assessment (POMA), Timed Up-and-Go test (TUG), 10-min walking test  2. Walking adaptability – e.g., 10-min walking test with obstacles and 10-min walking test with cognitive task | 1. Fear of falling – Falls Efficacy Scale International (FES-I)  2. Fall incidence  3. General health – e.g., VAS, Hip Disability and Osteoarthritis Score – Quality of life (HOOS-Q) | ↔ walking ability, fear of falling and fall incidence |
| **Multidisciplinary, multifactorial intervention programs** | | | | | | | |
| Cook et al. [116], Zusman et al. [117]  (To examine  the effect of a multidisciplinary clinic on sedentary behavior and physical activity and provide descriptions of activity patterns over 1 year for men and women.) | 53; 80 | 1:1 | Intervention (*Outpatient*); usual care plus outpatient management in an enhanced post-fracture clinic designed based on the need to assess both fall risk factors and bone health, led by a geriatrician with physiotherapist and occupational therapist. | Control (*Outpatient*); standard postoperative management after hip fracture provided within the local public health authority, which may have included prescribed calcium and Vitamin D and home rehabilitation services. | Mobility as measured by the Short Physical Performance Battery (SPPB) at 12 months | Sedentary behaviour and physical activity | ↔ sedentary behaviour and physical activity |
| Crotty et al. [118, 119]  (To compare hospital and home settings for the rehabilitation of  patients following hip fracture.) | 66; 82.5 | 1:1 | Home Care (*home*); patients were discharged from acute care within 48 hours of randomization and promptly visited by therapist from home rehabilitation interdisciplinary team (team coordinator, physiotherapist, occupational therapist, speech pathologist, social worker and therapy aid). Goals and therapy frequency was tailored to participants, where progress was revised at weekly case conferences. Prior to discharge, necessary modifications, installation of equipment or assistive aids in the home environment were made. | Conventional care (*inpatient*); patients received routine hospital care and rehabilitation in hospital, which included inpatient services, development of care pathways and discharge planning. | 1. Physical dependence  2. Incidence of falls | 1. Social dependence  2. Balance confidence  3. Quality of life (QoL)  4. Carer strain  5. Patient and carer satisfaction  6. Use of community services | ↑ physical independence and more confidence that they would avoid falling whilst undertaking activities of daily living for the least disabled group of fallers.  ↓ caregiver burden, possibly due to higher involvement in the decision making process and patients were only accepted into the trial if caregivers consented. |
| Galvard and Samuelsson [112]  (To compare the rehabilitation of hip fracture patients between the orthopedic department and the geriatric hospital.) | 371; 78.3 | 1:1 | Geriatric hospital (*Inpatient*); usual care plus once weekly visit by an orthopaedic surgeon who would decide on further treatment. | Orthopaedic department (*Inpatient*); usual care. | 1. Primary mortality  2. Discharge destination  3. Total first-year mortality  4. Number of hip prostheses during the first postoperative year. | Nil | ↔ comparable outcomes |
| Huusko et al. [120, 121]  (To evaluate the effect of intensive geriatric  rehabilitation on demented patients with hip fracture.) | 243; 80 | 1:1 | Intervention group (*Inpatient to home*); transferred to geriatric ward for about 2 weeks of intensive rehabilitation to promote early ambulation, self-motivation and function. An occupational therapist evaluated the need for daily living aids. Discharge and follow-up planning were checked at weekly team meeting with patient. Patients discharged to independent living were visited 10 times by a physiotherapist for individual exercises in the patient’s home during the first 2 weeks aft discharge. Participants were followed up for first 12 months after operation. | Control group (*Inpatient*); referred to local hospital wards for standard care. | 1. Continuous length of hospital stay (LOS)  2. Functional recovery  3. Mortality  4. Activities of daily living (ADL) and instrumental activities of daily living (IADL) | Mini mental state examination to screen for dementia | ↓ LOS  ↑ independence in IADL  ↑ patients with mild or moderate dementia to return to community and independent living |
| Karlsson et al. [122, 123], Berggren [124]  (To evaluate if Geriatric Interdisciplinary Home Rehabilitation could improve walking ability for  older people with hip fracture compared with conventional geriatric care and rehabilitation. A secondary aim was to investigate the postoperative length of hospital stay (LOS).) | 205; 82.9 | 1:1 | Geriatric Interdisciplinary Home Rehabilitation (GIHR) (*Inpatient to home*); multifactorial rehabilitation program, including comprehensive geriatric assessment (CGA) with focus on detection, prevention, and treatment of postoperative complication. Nearly daily home visits from someone in the GIHR team (nurse, occupational therapist and physiotherapists) during the first days after discharge. | Conventional Geriatric Care and Rehabilitation (*Inpatient*); interdisciplinary rehabilitation using CGA, rehabilitation after discharge were referred to primary health care, geriatric outpatient rehabilitation unit or physiotherapists and occupational therapists in residential care facilities. | 1. Walking ability indoors and outdoors  2. Gait speed  3. Postoperative length of hospital stay (LOS) | 1. Independence in ADL  2. Complications, readmissions and total days spent in hospital after discharge | ↔ walking ability in the short and long term  ↓ LOS  ↔ complications, readmissions, or days spent in hospital after discharge |
| Kennie et al. [113], Reid et al. [114]  (To compare postoperative collaborative  care between orthopaedic surgeons and  physicians in geriatric medicine with routine orthopaedic  care in elderly women with proximal femoral  fracture.) | 108;79 (median of treatment group) | 1:1 | Treatment (*Inpatient*); transferred to orthopaedic beds in a peripheral hospital, a general practitioner provided day to day medical attention, a consultant physician in geriatric medicine attended 2 ward rounds and 1 conference of the multidisciplinary team each week. An orthopaedic specialist’s advice was available on demand. | Control (*Inpatient*); physiotherapy, occupational therapy, and orthotic services, regular attention on orthopaedic ward rounds, and the demand for beds encouraged early discharge. | 1. Physical independence  2. Residence after discharge  3. Length of hospital stay (LOS) | 1. Independence in activities of daily living  2. Mental status  3. Carer strain  4. Life satisfaction index | ↓ LOS  ↑ functional independence and independent living |
| Naglie et al. [115]  (To compare the effectiveness of inpatient  interdisciplinary care with that of usual care for elderly patients with hip fracture.) | 279; 84.2 | 1:1 | Interdisciplinary care ward (*Inpatient*); routine assessment and care by an internist-geriatrician, physiotherapist, occupational therapist, social worker and clinical nurse specialist, as well as twice-weekly interdisciplinary rounds to set goals for patients and monitor their progress. Received routine postoperative care and daily medical care by senior internal medicine resident supervised by an internist-geriatrician. | Usual care ward (*Inpatient*); patients only have access to allied health care professionals if consultation was requested, had limited access to an OT or clinical nurse specialist, no interdisciplinary rounds, and only received routine postoperative surgical care. | Proportion of patients alive with no decline in ambulation, transfers in and out of a chair and bed, and place of residence | Modified Barthel Index scores, instrumental activities of daily living (IADL), 6-month health care utilization | ↔ 3- and 6-month outcomes |
| **Osteoporosis management / Fracture prevention post-discharge** | | | | | | | |
| Colón-Emeric et al. [164], Lyles et al. [166], Eriksen et al. [167], Colón-Emeric [168], Boonen et al. [169], Adachi et al. [163], Prieto-Alhambra et al. [165], Magaziner et al. [170]  (To test the efficacy and safety of zoledronic acid (at a dose of 5 mg) administered intravenously once yearly for the prevention of new clinical fractures in women and men who had undergone recent surgical repair of a hip fracture.) | 2127; 74.5 | 1:1 | Zoledronic acid (*Outpatient*); dose of 5mg, intravenous infusion within 90days after the surgical repair of a hip fracture and every 12 months thereafter for the duration of the study. Thereafter, daily supplementation with oral calcium and vitamin D. | Placebo (*Outpatient*); placebo infusion within 90days after surgical repair of hip fracture, followed by daily supplementation with oral calcium and vitamin D. | New clinical fracture, excluding facial and digital fractures and fractures in bone containing metastases. | 1. Change in bone mineral density in the nonfractured hip  2. New vertebral, nonvertebral and hip fractures  3. Prespecified safety end points including death  4. Delayed hip fracture healing | ↓ new clinical fracture (with and without cognitive impairment)  ↑ survival rate  ↑ quality of life at 24 months  ↑ total hip (TH) and femoral neck (FN) BMD  ↔ fracture healing |
| Davis et al. [127]  (To determine whether a novel Patient Empowerment and Physician Alerting (PEPA) intervention  would improve the proportion of seniors who were investigated and treated for osteoporosis after hip fracture.) | 48; 81.5 | 1:1 | PEPA (Patient Empowerment and Physician Alerting) Intervention (*Inpatient to outpatient*); usual care plus three  Elements  (i) Osteoporosis information and letter for participants to encourage them to return to their Primary Care Physicians (PCP) for further investigation.  (ii) Request for participants to take a letter from orthopaedic surgeon to the PCP alerting them to the hip fracture and encouraging osteoporosis investigation.  (iii) Telephone call at 3 months and 6 months to determine whether osteoporosis investigation and treatment had occurred. | Usual Care (*Inpatient to outpatient*);  (i) Usual care  (ii) Osteoporosis information and letter for participants to encourage them to return to their PCPs for further investigation | Diagnosis and Management  Questionnaire (DMQ) to determine the proportion  of participants who were offered one or more osteoporosis  ‘best practices’  Clinical outcomes: (i) investigation  (dual-energy x-ray absorptiometry [DXA] scan, yes/no)  (ii) treatment (bisphosphonate therapy, yes/no; calcium and  vitamin D, yes/no; exercise prescribed, yes/no) | Nil | ↑ clinical management and patient – primary care physician communications on osteoporosis investigation and treatment |
| Glendenning et al. [171], [172]  (To determine if ergocalciferol and cholecalciferol are equipotent therapies in  vitamin D-insufficient hip fracture patients.) | 95; 83 | 1:1 | 1. Cholecalciferol (*Inpatient to home*); 1000 IU/day for 3 months.  2. Ergocalciferol (*Inpatient to home*); 1000 IU/day for 3 months.  Both include supplemental calcium (calcium carbonate  600 mg daily). | Nil | Total serum 25OHD measured by high performance liquid chromatography (HPLC). | Radioimmunoassay (RIA) measured 25-hydroxyvitamin D (25OHD), intact parathyroid hormone  (iPTH), and bioactive (1–84) whole PTH (wPTH). | ↑ total 25OHD levels in cholecalciferol group but ↔ calculated free and bioavailable vitamin D metabolite concentrations in both groups |
| Grant et al. [129]  (To assess whether vitamin D3 and calcium, either alone or in combination, were effective in prevention of secondary fractures.) | 5292; 77 | 1:1:1:1 | Intervention (*Home*); participants were assigned two tablets with meals daily consisting of  1. 800 IU vitamin D3  2. 1000 mg calcium (given as carbonate)  3. 800 IU vitamin D3 + 1000 mg calcium  Participants were asked to take tablets until trial closure (between 24 and 63 months). | Placebo (*Home*); 2 placebo tablets daily until trial closure (between 24 and 63 months). | All new low-energy fractures | Health status, hospital admission, change of residence, falls, possible adverse events | ↔ incidence of new fractures, other strategies should be considered for secondary prevention |
| Majumdar et al. [125], Beaupre et al. [126]  (To compare an osteoporosis case manager intervention with usual care to improve quality of care for patients with hip fracture.) | 220; 74 | 1:1 | Osteoporosis Case Manager (*Outpatient*); In addition to usual care, Case Manager provided additional one-to-one counseling about the importance of bone mineral density (BMD) testing and the ability of bisphosphonate therapy and other treatments to reduce the risk of future fractures, arranged for outpatient BMD test and for local community pharmacies to dispense prescriptions written by a study physician for alendronate, 70mg/week, or risendronate, 35mg/week, for patients with low bone mass. | Usual Care (*Outpatient*); Study personnel provided counseling about fall prevention and the need for additional intake of calcium and vitamin D, provided educational materials for patients and caregivers to discuss with their primary care physician, and arranged BMD test. | Receipt of bisphosphonate therapy within 6 months of hip fracture. | Bone mineral density testing, appropriate care (bone mineral density testing and treatment if bone mass was low), and intervention costs. | ↑ quality of osteoporosis care (osteoporosis treatment, BMD testing, delivery of appropriate care)  ↓ all-cause mortality with oral bisphosphonates |
| Miki et al. [128]  (To compare the effect of osteoporosis management initiated by the orthopaedic team and osteoporosis management  initiated by the primary care physician on the rates of treatment at six months.) | 62; 79.2 | 1:1 | Intervention Group (*Inpatient to outpatient*); osteoporosis evaluation in the hospital and prior to discharge, were given a follow-up appointment between two weeks and one month postoperatively in a specialized orthopaedic osteoporosis clinic, started on 1500 mg of calcium and 800 IU of vitamin D3 daily prior to discharge. | Control Group (*Inpatient to outpatient*); prior to discharge, patients and their families were instructed to approach their primary care physicians for an osteoporosis evaluation, started on 1500 mg of calcium and 800 IU of vitamin D3 daily prior to discharge. | Number of patients on osteoporosis treatment at 6 months after fracture | Nil | ↑ rates of early osteoporosis treatment |
| **Post-operative Anaemia** | | | | | | | |
| Parker [136]  (To determine if oral iron therapy is beneficial for the treatment of anemia after surgery for the treatment of a hip  fracture.) | 300; 82 | 1:1 | Iron therapy (*Inpatient to home*); 28 days of oral iron therapy (ferrous sulfate, 200mg twice daily). | No iron therapy (*Inpatient to home*) | Correction of anemia at the time of outpatient follow-up visit 6 weeks after discharge. | Nil | ↔ clinically relevant benefit in correcting anemia |
| Prasad et al. [137]  (To establish the effect of oral iron  supplementation on haemoglobin level at 4 weeks post-operative in elderly patients with fractured  neck of femur undergoing surgical treatment.) | 66; 82 | 1:1 | Study group (*Inpatient to home*); oral ferrous sulphate 200mg 3 times a day from post-operative day 2 for 4 weeks. | Control group (*Inpatient to home*); no iron supplements. | Haemoglobin levels 4 weeks post-operation. | Nil | ↑ haemoglobin value at 4 weeks |
| Zauber et al. [135]  (To assess the efficacy of oral iron therapy in the recovery of  patients' hemoglobin levels after major surgery.) | 79; 75 | 37:42 | Iron supplement (*Inpatient*); oral ferrous sulfate dose of 325 mg/day, 4 times a day for the period of their hospitalization, starting from the 2^nd^ postoperative day. | Control (*Inpatient*); no iron supplement | Changes in haemoglobin levels and reticulocyte counts over 2- to 3-week follow up | Nil | ↔ haemoglobin levels for patients with adequate iron stores |
| **Supported Discharge** | | | | | | | |
| Krichbaum et al. [138]  (To determine the effectiveness of a  Nursing intervention model, the gerontologic advanced practice nurse post-acute care coordinator (GAPN PACC) care model to improve elders’ outcomes following hip fracture.) | 33; 78.87 | 1:1 | Treatment group (*Inpatient to home*); Gerontologic Advance Practice Nurse (GAPN) Post-acute Care Coordinator. 6 months of care activities including: GAPN–elder interactions once a week in the first month post-discharge from the hospital and twice per week until 6 months after surgery. | Control group (*Inpatient to home*); care after hip fracture  according to the hospital and individual surgeon’s protocols and had no assigned continuous care coordinator or care manager for the post-acute care period. | 1. Self-rated health  2. Level of depression  3. Functional status based on the ability to perform activities of daily living (ADL) and instrumental ADLs (IADL)  4. Living situation | Nil | ↑ most ADLs and IADLs (mobility, household chores and personal care) at 12 months |
| **Falls prevention (inpatient, post-discharge)** | | | | | | | |
| Di Monaco et al. [174]  (To assess the effectiveness of a single telephone call by an occupational therapist in reducing the proportion of fallers and improving the adherence to targeted recommendations for fall prevention after hospital discharge in hip-fracture women.) | 153; 79 | 1:1 | Intervention group (*Home*); usual rehabilitation protocol plus a telephone call by an occupational therapist at a median of 18 days after discharge, to check environmental hazards, behaviours in activities of daily living, and use of assistive devices and reinforced targeted modifications to prevent falls. | Control group (*Inpatient*); usual rehabilitation protocol. | Proportion of fallers | Improve adherence to targeted recommendation for fall prevention after hospital discharge | ↔ proportion of fallers or adherence to targeted fall prevention recommendations |
| Lockwood et al. [134]  (To investigate whether home assessment visits prior to hospital  discharge for patients recovering from hip fracture reduce falls and prevent hospital readmissions, within the first 30 days and 6 months after discharge home.) | 77; 82.15 | 1:1 | Intervention (*Inpatient and home*); usual care that included assessment and management by a multidisciplinary team; and a single home visit in which an occupational therapist accompanied the subject on a short home visit (approximately 1 hour) between 1 and 5 days prior to discharge, and provided education, advice and recommendations on equipment, home adaptations and community support services. | Control (*Inpatient*); usual care that included assessment and management by a multidisciplinary team. | 1. Falls  2. Hospital readmissions | 1. Concern about falling  2. Functional independence  3. Independence in instrumental activities of daily living  4. Functional ability or participation  5. Health-related quality of life | ↓ hospital readmissions  ↑ functional independence at 6 months which may have ↓ risk of falls |
| Scheffers-Barnhoorn et al. [173]  (To evaluate the effect of the Fear of falling InTervention in HIP fracture geriatric rehabilitation (FIT-HIP) intervention in patients with fear of falling (FoF) in geriatric rehabilitation (GR) after hip fracture.) | 77; 82.5 | 1:1 | Treatment (*Inpatient*); a multicomponent cognitive behavioural intervention conducted by physiotherapist, embedded in usual care in geriatric rehabilitation. Intervention is carried out for the duration of patients’ admission to GR unit, which averaged to 6 weeks. | Control (*Inpatient*); usual care. | Mean difference in Tinetti Performance Oriented Mobility Assessment, Mean difference in Falls Efficacy Scale International (FES-I) | Self-reported activity restriction due to Fear of Falling (FoF), standardised measure on (in)dependence in walking ability, sociodemographic information, therapy intensity, adverse events | ↔ FoF or functional outcome in early rehabilitation |
| Stenvall et al. [130, 131], Berggren et al. [132], Olofsson et al. [133]  (To evaluate if a postoperative multidisciplinary,  multifactorial intervention program could reduce inpatient  falls and fall-related injuries in patients with femoral neck  fractures.) | 199; 82.2 | 1:1 | Intervention group (*Inpatient*); geriatric unit specializing in geriatric orthopaedic patients – comprehensive geriatric assessments, management and rehabilitation with active prevention, detection, and treatment of postoperative complications such as falls, delirium, pain and decubitus ulcers. | Control group (*Inpatient*); specialist orthopaedic unit following the conventional postoperative routines. | 1. Falls during hospitalization – number of falls and time lapse to first fall after admission, injuries resulting from fall  2. Length of stay  3. Morbidity and mortality  4. Modified Organic Brain Syndrome Scale (OBS Scale)  5. Geriatric Depression Scale  6. Vision and hearing | 1. Walking ability and ADL performance at 4 and 12 months postoperative  2. Readmissions and in-hospital days after discharge at 4 and 12 months postoperative  3. Falls at 4 and 12 months postoperative  4. Mini Nutritional Assessment (MNA) scale | ↓ inpatient falls, even in patients with dementia, ↔ falls after discharge  ↑ independence in P-ADL (bathing, dressing , toileting, transferring, continence, and feeding) performance at 4 months and 12 months despite shorter hospitalization  Nutritional intervention may ↓ delirium, decubitus ulcers and hospitalization |
| **Medication, Nutrition and Supplementation** | | | | | | | |
| Adunsky et al. [88]  (To demonstrate an improvement in overall physical functional performance in  patients who had recently experienced a hip fracture, following  administration of MK-0677 (non-peptidyl GH-secretagogue mimetic) 25-mg once daily for 24 weeks relative  to placebo, and assess the safety and tolerability of MK-0677 in this  patient population.) | 123; 78.7 | 1:1 | MK-0677 25mg (*Inpatient to home*); daily MK-0677 25mg for 24 weeks, with supplemental vitamin-D_3_ (400IU/day) and multidisciplinary rehabilitation program of the individual centers. | Placebo (*Inpatient to home*); daily matching placebo for 24 weeks, with supplemental vitamin-D_3_ (400IU/day) and multidisciplinary rehabilitation program of the individual centers. | Change in functional power measurements:  1. Lower extremity strength and power  2. Physical function performance  3. Self-reported physical function | Safety and tolerability of MK-0677 | May ↑ functional power  No definitive conclusion regarding efficacy as study was terminated early due to adverse experiences |
| Anbar et al. [63]  (To evaluate  whether nutritional support guided by repeated measurements of  REE improved outcomes in geriatric patients following surgery for hip fractures and compared this to usual nutritional therapy.) | 50; 83 | 1:1 | Study group (*Inpatient*); oral nutritional supplements (ONS) started 24 h after surgery, provided in the form of Ensure plus (355 kcal/237ml and 13.5g protein) or Glucerna (237kcal/237ml and 9.9g protein), patient, family and caregivers were educated regarding the importance of nutritional support and more attention  was given to personal food preferences. | Control group (*Inpatient*); usual hospital food (standard or texture-adapted) and a fixed dose of ONS if already prescribed prior to hospitalization. | 1. Postoperative complications  2. Hospital length of stay | 1. Energy intake  2. Calculated energy balance | ↓ postoperative complications and length of hospital stay |
| Aquilani et al. [75]  (To investigate whether supplementation with essential amino acids  (EAAs) could improve hypoAlb and anaemia in rehabilitative elderly subjects with HF surgery and mild hypoAlb and anaemia.) | 112; 82.3 | 1:1 | EAA Group (*Inpatient to home*); oral nutritional mixture supplement, which provided 8g of EAAs/day for 60 days, plus rehabilitation. | Casein Group (*Inpatient to home*); similar isocaloric, isonitrogenous (casein) product for 60 days, plus rehabilitation. | Baseline circulating proteins | Nil | May ↑ recovery of hypoalbumenimia and anaemia in more than two thirds and one third, respectively, of inflamed elderly patients after hip fracture surgery |
| Aspenberg et al. [86], Malouf-Sierra et al. [87]  (To study the effects of teriparatide in  comparison with risedronate on recovery after pertrochanteric hip fractures.) | 171; 76.8 | 1:1 | Teriparatide (*Inpatient to home*); started on 20µg teriparatide subcutaneous injection once daily plus oral placebo once weekly for 26 weeks, after which treatment was unblinded and patients continued with the same treatment for a total of 78 weeks. | Risedronate (*Inpatient to home*); placebo subcutaneous injection once daily plus oral risedronate 35mg once weekly for 26 weeks, after which treatment was unblinded and patients continued with the same treatment for a total of 78 weeks. | Change in lumbar spine BMD from baseline to 78 weeks. | Functional outcomes: functional mobility, self-reported hip pain, patient-reported health status. | ↓ pain and ↑ functional mobility between 6 and 26 weeks  ↑ lumbar spine and femoral neck BMD |
| Bhandari et al. [85]  (To further evaluate the potential effect of teriparatide (bone anabolic drug) on  fracture healing.) | 159; 70 | 1:1 | Teriparatide (*Inpatient to home*); once daily subcutaneous injection of 20µg teriparatide, including supplemental calcium (≤ 1000mg/day) and vitamin D (≤ 4000 IU/day) for 6 months. | Placebo (*Inpatient to home*); once daily subcutaneous injection of 20µg placebo (identical pen injection devices), including supplemental calcium (≤ 1000mg/day) and vitamin D (≤ 4000 IU/day) for 6 months. | Effect of 6 months of teriparatide treatment versus placebo on the proportion of patients with no revision surgery 12 months after internal fixation of a low-trauma femoral neck fracture | 1. Radiographic evidence of fracture healing  2. Pain control  3. Recovery of ambulation  4. Composite successful fracture healing  5. Adverse events | ↔ frequency of revision surgery or radiographic fracture healing at 12 months, pain control and functional outcomes  ↑ recovery of ambulation |
| Botella-Carretero et al. [154]  (To evaluate the efficacy of oral nutritional supplements (ONS)  on the nutrition status of normally nourished or only mildly  undernourished geriatric patients submitted to surgery for hip fracture.) | 90; 83.8 | 2:1 | 1. Protein-powder-ONS (*Inpatient to home*); protein supplementation in the form of commercial protein powder (10-g packets, with each providing 9 g of protein and 38 kcal) dissolved in water or in the diet’s milk or soup, to aim at 36 g of protein per day (4 packets per day). 2. Energy-protein-ONS (*Inpatient to home*); energy and protein supplements by means of commercial enteral nutrition for oral intake (200-mL bricks, with each providing 18.8 g of protein and 250 kcal) to aim at 37.6 g of protein and 500 kcal per day (2 bricks per day). | Control; no intervention. | Nutrition status of patients  at discharge from hospital, by means of changes in serum albumin, prealbumin, and retinol-binding globulin (RBG). Changes in weight, BMI, midbrachial circumference, and tricipital fold were also considered. | Tolerance to the prescribed ONS, length of hospital stay, postoperative complications, and the time from surgery to the start of mobilization as included in the rehabilitation program. | ↔ nutrition status in normally nourished or only mildly undernourished geriatric patients with hip fracture submitted to surgery, who have short hospital stay and no postoperative complications |
| Duncan et al. [70]  (To examine how improved attention to nutritional status and dietary intake, achieved through the employment of  dietetic assistants (DAs), will affect postoperative clinical outcome among elderly women with hip fracture.) | 302; >65 | 1:1 | DA Care (*Inpatient*); conventional care plus 2 part-time dietetic assistants (one of them present on the ward 6h per day, 7 days a week) who worked closely with a specialist dietician to ensure that patients allocated to them received appropriate help in meeting their nutritional needs. | Conventional (*Inpatient*); routine provision of oral nutritional supplements to all patients. | Postoperative mortality in the acute trauma unit | 1. Inpatient and 4-month mortality  2. Length of stay (LOS)  3. Acute ward complication rate 4. Energy intake and nutritional  status | ↓ postoperative mortality at 4 months  ↑ energy intake and nutritional status  ↔ LOS |
| Eneroth et al. [64, 65]  (To evaluate nutritional status and fluid and energy intake during the first ten days of hospitalisation in a selection of otherwise healthy patients with a hip  fracture.) | 80; 81 | 1:1 | Treatment group (*Inpatient*); hospital diet and 1000ml Vitrimixs daily for three days from day 1, followed by a seven day oral treatment with Fortimels, 2x200ml daily. | Control group (*Inpatient*); ordinary hospital food and beverage. | Evaluate the fluid and energy intake during hospital stay and the difference  between actual daily intake and the required  intake in otherwise healthy patients with hip fractures without mental impairment | 1. Analyse whether supplementary nutritional  intake in a randomised prospective setting lowered the ordinary food and beverage intake.  2. Complications: (1) infections (wound infection,  urinary infection, or pneumonia), (2) other complications  (thrombophlebitis, deep venous thrombosis, pulmonary embolism,  pulmonary edema, or myocardial infarction), and (3) death | ↑ energy and fluid intake in  patients with hip fracture to near needed levels  ↓ complication rates and mortality at 120 days |
| Espaulella et al. [155]  (To determine whether a nutritional supplement may (i) help elderly patients return to pre-fracture functional levels 6 months post-fracture and (ii) decrease fracture-related complications and mortality.) | 171; 82.6 | 1:1 | Treatment (*Inpatient to outpatient*); nutritional supplement, providing 149 calories per dose (20 g of protein, 800 mg of calcium, 25 IU of vitamin D3 and other minerals and protein), 200 ml/day, once a day for 60 days. | Placebo (*Inpatient to outpatient*); placebo supplement, providing 155 calories per dose (mainly carbohydrates), 200 ml/day, once a day for 60 days. | Functional status (Barthel index score, mobility index score, use of walking aids), nutritional status, complications | Compliance to treatment | Slight ↓ in post-operative complications  ↔ recovery of functional status and length of hospital stay |
| Flodin et al. [92, 93]  (To evaluate the effects of postoperative treatment with calcium, vitamin D, and bisphosphonates (alone or together) with nutritional supplementation on total hip and total body bone mineral density (BMD).) | 79; 79 | 1:1:1 | 1. Intervention N (*Inpatient to home*); same as control plus 200ml package twice daily, each containing 20g of protein and 300kcal for the first 6 months following hip fracture and was combined with risedronate, 35mg once weekly for 12 months.  2. Intervention B (*Inpatient to home*); same as control plus risedronate, 35mg once weekly for 12 months. | Controls (*Inpatient to home*); two daily doses of calcium 1g and cholecalciferol for 12 months. | 1. Body composition  2. Handgrip strength  3. Health-related quality of life | 1. Bone resorption marker C-terminal telopeptide of collagen I (serum CTX-I)  2. Serum levels of 25-hydroxy vitamin D (25OHD) and parathyroid hormone (PTH) | ↑ total body BMD and total hip BMD  ↔ handgrip strength (HGS) and health-related quality of life (HRQoL) inter-group  ↑ HGS and HRQoL within group N |
| Harwood et al. [74]  (To compare the effects of different calcium and vitamin D supplementation regimens on bone biochemical markers, bone mineral density and rate of falls in elderly women post-hip fracture.) | 150; 81.2 | 1:1:1:1 | 1. Injected vit D (*Inpatient*); single injection of 300,000 units of vitamin D_2._  2. Injected vit D + oral Ca (*Inpatient to home*); injected vit D_2_ + 1 g/day oral calcium.  3. Oral vit D + oral Ca (*Inpatient to home*); 800 units/day oral vitamin D_3_ + 1 g/day oral calcium. | Control; no treatment. | 25-hydroxyvitamin D (25OHD), Intact parathyroid hormone (PTH), Bone mineral density (BMD) | Vital status, Falls, Mobility at 3 months | ↓ parathyroid hormones, falls  ↑ bone density  Effects may be more marked with calcium co-supplementation. |
| Hedström et al. [71]  (To investigate whether growth hormone (GH) given postoperatively  could increase the levels of serum insulin-like growth factor-I (IGF-I) in  elderly patients with hip fracture and reduce the loss of lean body mass and body mineral content (BMC) after a hip fracture without major side-effects.) | 20; 83.9 | 1:1 | Treatment (*Inpatient*); mean growth hormone (Genotropin, Kabi-Pharmacia) dose of 5.8 IU/day, injected subcutaneously once daily, for a period of 4 weeks, starting from 2-5 days post-operation. | Placebo (*Inpatient*); equivalent placebo volume injected subcutaneously once daily for a period of 4 weeks, starting from 2-5 days post-operation. | Serum insulin-like growth factor-I (IGF-I), IGF-I binding protein-1 (IGFBP-1), Body composition (fat and lean body mass, muscle volume, bone mineral density, subcutaneous fat), bone mineral content (BMC) | Quadriceps muscle force, blood samples content (e.g. hemoglobin concentration, blood platelet count) | ↑ serum IGF-I, BMC and lean body mass |
| Hedström et al. [81]  (To investigate  whether an anabolic steroid given with vitamin D and calcium could reduce postoperative catabolism and give a better outcome after a fracture of the hip without serious  adverse effects.) | 63; 80.5 | 1:1 | Anabolic group (*Inpatient to outpatient*); treatment for 1 year with nandrolone decanoate (35mg intramuscularly every 3^rd^ week), a daily supplement of 1alpha-hydroxylated vitamin D3 (alphacalcidol 0.25µg) and calcium (500mg). | Control group (*Inpatient to outpatient*); treatment for 1 year with daily calcium (500mg) only. | 1. Bone mineral density (BMD)  2. Thigh muscle volume | 1. Harris hip score  2. Pain assessment  3. Gait speed  4. Activities of daily living (ADL) score | ↑ body composition, BMD and clinical function |
| Hitz et al. [157]  (To evaluate the effect of 1 year of treatment with calcium and vitamin D on bone mineral density (BMD) and bone markers in patients with a recent low-energy fracture.) | 29; 73.9 | 2:3 | Treatment (*Inpatient to home*); oral calcium carbonate dose of 3000 mg/day + oral cholecalciferol dose of 1200 IU/day, with a multivitamin tablet of 200 IU of cholecalciferol. Tablets are taken 3 times daily, for a period of 1 year. | Placebo (*Inpatient to home*); placebo tablets with multivitamin tablet of 200 IU of cholecalciferol. Tablets are taken 3 times daily, for a period of 1 year. | Bone mineral density (BMD), physical performance in standardised test, Serum concentration (e.g. Vitamin D status, parathyroid hormone, osteocalcin) | Nil | ↑ vitamin D concentration and BMD, related to physical performance |
| Huusko et al. [82]  (To evaluate the  short-term outcome of intranasal calcitonin treatment of  elderly hip fracture patients on pain, bone loss, functional  recovery, and length of hospital stay.) | 229; 80.1 | 1:1 | Treatment (*Inpatient to outpatient*); intranasal salmon calcitonin 200 IU/day for 3 months. | Placebo (*Inpatient to outpatient*); placebo nasal spray for 3 months. | Pain, length of hospital stay, bone mineral density (BMD) loss, functional recovery in activities and instrumental activities of daily living, complications | Fusion of hip fractures treated with internal fixation using a screw or a nail | ↓ bone loss and pain  ↔ functional recovery, length of stay, mortality, or in complications  ↑ fusion of hip fractures treated with internal fixation with a screw or nail |
| Invernizzi et al. [76]  (To evaluate the impact of a 2-month rehabilitative protocol combined with dietetic counselling, with or without essential  amino acid supplementation, on functioning in hip fracture patients.) | 32; 79.03 | 1:1 | 1. Group A (*Home*); 2 months of essential amino acid supplementation and specific rehabilitative program consisting of 5 sessions of 40 min each per week for 2 weeks, supervised by an experienced physiotherapist, and received a dietetic counselling.  2. Group B (*Home*); specific rehabilitative program consisting of 5 sessions of 40 min each per week for 2 weeks, supervised by an experienced physiotherapist, and received a concomitant dietetic counselling, without essential amino acid supplementation. | Nil | 1. Appendicular muscle strength  2. Physical performance  3. Level of assistance | 1. Nutritional assessment  2. Health-related quality of life | ↑ function and ↓ disability, in particular sarcopenic patients |
| Kim et al. [89]  (To determine whether the timing of the post-operative administration of bisphosphonate affects fracture healing and the rate of complication following an intertrochanteric fracture.) | 90; 76.1 | 1:1:1 | Groups A, B and C (*Inpatient to home*); all received oral bisphosphonate (Risendronate, 35mg) weekly with a daily calcium intake of 1200mg/day and cholecalciferol at 800 IU/day.  Group A started one week after surgery, Group B one month after surgery and Group C three months after surgery. Bisphosphonate treatment was continued for a minimum of one year. | Nil | Time to healing of the fracture | The incidence of complications, including excessive displacement and revision surgery | ↔ all outcomes |
| Laiz et al. [77]  (To determine whether 3-monthly supplementation of an oral vitamin D widely used in Spain (calcifediol) plus daily exercise could influence survival at one and four years after surgery for osteoporotic hip fracture.) | 88; 82.5 | 1:1 | Intervention group (*Home*); 3-monthly single dose of 3 mg of calcifediol taken orally, and individual instruction that is specifically developed by the investigators and the physiotherapists about exercises for lower limbs and an illustrated leaflet. | Non-intervention group (*Home*); 3-monthly single dose of placebo taken orally, and standard health recommendations to execute physical activity and muscle-strengthening to improve balance and prevent falls. | Survival at one-year and four-year follow up. | 1. New fractures  2. Medical complications  3. Compliance with anti-osteoporotic treatment at one-year post surgery | ↑ survival rate  ↓ medical complications |
| Mak et al. [94, 95]  (To evaluate the effect of an initial loading dose of vitamin D to improve rehabilitation outcomes following  hip fractures.) | 218; 83.9 | 1:1 | Active (*Inpatient to outpatient*); oral loading dose of vitamin D3 (5 tablets of 50,000 IU) within 96 hours or up to 7 days post-surgery, oral maintenance vitamin D3 and calcium and will follow the usual hip fracture rehabilitation pathway. | Placebo (*Inpatient to outpatient*); 5 placebo tablets identical in appearance to the active tablets, within 96 hours or up to 7 days post-surgery, oral maintenance vitamin D3 and calcium and will follow the usual hip fracture rehabilitation pathway. | Gait velocity over 2.4 m measured at weeks 2, 4, and 26, with baseline  assumed to be 0 immediately following surgery | 1. Number of falls, fractures and hospitalizations  2. Activities of daily living  3. Quality of life  4. 25-OHD  and calcium levels  5. Grip strength  6. Adherence to calcium and  vitamin D supplements  7. Adverse events (including cardiovascular  events and death) | ↔ gait velocity, fractures at 4 weeks  ↓ number of falls, pain  ↑ vitamin D (25-OHD) |
| Malafarina et al. [66, 67]  (To assess  whether oral nutritional supplementation (ONS) improves muscle mass and nutritional markers (BMI, proteins)  in elderly patients with hip fracture.) | 107; 85.4 | 1:1 | Intervention group (IG) (*Inpatient*); standard diet plus oral liquid nutritional supplement enriched with β-hydroxy-β-methylbutyrate (HMB), calcium and vitamin D (2 bottles a day, 1 in the morning and 1 in the afternoon). | Control group (CG) (*Inpatient*); standard diet. | 1. BMI  2. Muscle mass  3. Functional situation | Nil | ↑ muscle mass and functional recovery |
| Mw et al. [68]  (To investigate the clinical, nutritional and rehabilitation effects of an oral nutritional supplementation  (ONS) in an inpatient rehabilitation setting.) | 121; 81.3 | 1:1 | ONS group (*Inpatient*); The oral nutritional supplementation  (ONS) was a drink of about 240 ml (18–24 g protein and 500 kcal per day) in volume given twice daily on top of the standard hospital diet, for a maximum duration of 4 weeks (or until discharge, whichever came first) in addition to best medical care. Also given oral vitamin D supplement of 800–1,000 IU per day and  calcium tablets containing elemental calcium of 1,200 mg. | Control group (*Inpatient*); oral vitamin D supplement of 800–1,000 IU per day and calcium tablets containing elemental calcium of 1,200 mg.  Both groups received rehabilitation therapy and regular case conference review until assessed to be fit to be discharged. | 1. Serum albumin level  2. Body mass index (BMI)  3. Functional  independence measure (FIM) score  4. Elderly  mobility scale (EMS) | 1. Frequency and severity of complications  2. Length of stay in rehabilitation ward  3. Mortality and accident and emergency  department attendance within 6 months after discharge  4. Mid-arm circumference  (MAC), triceps skin fold (TSF), serum insulin-like growth factor-1 (IGF-1) level, bilateral quadriceps strength  and dominant hand grip strength.  5. Patient tolerability, compliance  and adverse effects due to the supplementation | May ↓ LOS and number of complications  ↔ muscle strength and other nutritional parameters |
| Neumann et al. [156]  (To compare clinical outcomes with a standard (Ensure) or a high-protein (Boost HP) liquid nutritional supplement for older adults recovering from hip fracture surgery in a rehabilitation hospital.) | 46; 83.2 | 1:1 | High-protein (*Inpatient to outpatient*); standard diet with daily dose of 30 g/d protein of high protein liquid nutritional supplement Boost HP for 28 days. | Control (*Inpatient to outpatient*); standard diet with daily dose of 17.8 g/d protein of regular liquid nutritional supplement Ensure for 28 days. | Change in Mobility subscale of Functional Independence Measure (FIM) between rehabilitation admission and discharge | Length of rehabilitation stay, laboratory measures (i.e., serum albumin, prealbumin and C-reactive protein), physical activity energy expenditure by 7-d triaxial accelerometry, dietary intake by three random, telephonic, 24-h dietary recall | ↔ mobility subscale of FIM  May ↑ protein status and serum albumin |
| Niitsu et al. [69]  (To investigate the effect of resistance training in combination  with whey protein intake in the early postoperative period.) | 38; 79.7 | 1:1 | Whey protein group (*Inpatient*); Ingestion of whey protein (32.2g of protein, 2.0g of lipid, 3.8g of carbohydrate and other ingredients) started the day after surgery, in addition to rehabilitation, for 2 weeks. | Control group (*Inpatient*); Same rehabilitation programme, no placebo nutritional supplementation given. | 1. Knee extension strength  2. Activities of daily living (ADL)  3. C-reactive protein  4. Rest pain and motion pain | Nil | ↑ knee extension strength in both lower limbs and ADL (transfer, walking and toilet use)  ↔ C-reactive protein and pain |
| Papaioannou et al. [96]  (To examine whether large loading doses in addition to daily vitamin D  offered any advantage over a simple daily low-dose vitamin D regimen for increasing vitamin D levels.) | 65; 78.4 | 1:1:1 | 1. Group B (*Inpatient to home*); 50,000 IU vitamin D_2_ oral loading dose on Day 1, followed by daily tablet of 1,000 IU vitamin D_3_ for 90 days.  2. Group C (*Inpatient to home*); 100,000 IU vitamin D_2_ oral loading dose on Day 1, followed by daily tablet of 1,000 IU vitamin D_3_ for 90 days. | Group A (*Inpatient to home*); Placebo bolus loading dose on Day 1, followed by daily tablet of 1,000 IU vitamin D_3_ for 90 days. | Serum 25-hydroxy vitamin D (25-OHD) at baseline, 4-weeks and 3-months | Nil | ↔ 25-OHD levels  patients with acute hip fracture may benefit from a higher daily dose of vitamin D |
| Rondanelli et al. [73]  (To assess the efficacy on pain (assessed by VAS) of a supplement with a special formulation of essential amino acids (EAA) versus placebo in elderly patients admitted for rehabilitation management after surgical treatment of hip fracture.) | 40; 83.4 | 1:1 | Treatment (*Inpatient*); powdered amino acids supplement of 6.6 gram taken with water or milk, taken twice a day for 4-weeks. | Control (*Inpatient*); placebo consisting an isocaloric amount of maltodextrin, with the same appearance and flavour as the intervention product, taken twice a day for 4 weeks. | Pain | Body composition, nutritional status, quality of life, muscle function, hip functionality | ↓ pain, associated with ↑ duration of physio session |
| Schürch et al. [91]  (To investigate whether oral protein supplements benefit bone metabolism in patients with recent hip fracture.) | 82; 80.7 | 1:1 | Treatment (*Inpatient to home*); oral protein supplement, containing 20 g of proteins, 3.1 g of lipids, 35.7 g of carbohydrates and other vitamins and minerals, 65 g/day, 5 days a week for 6 months. | Placebo (*Inpatient to home*); isocaloric dose of placebo supplement, containing 54.4g of carbohydrates, 5 days a week for 6 months. | Bone mineral density, biochemical markers of bone remodelling, calciotropic hormone levels, biochemically evaluated nutritional and immunologic status, muscle strength | Length of hospital stay | ↑ serum levels of insulin-like growth factor-I and muscle strength  ↓ proximal femur bone loss and length of hospital stay |
| Sloan et al. [72]  (To determine the safety and efficacy of the anabolic  steroid nandrolone in elderly patients with hip fractures.) | 29; 82 | 1:1 | Nandrolone (*Inpatient*); weekly injections of nandrolone decanoate (2mg/kg), for the duration of hospital stay or until 4 weeks, whichever is earlier. | Control (*Inpatient*); placebo injections for the duration of hospital stay or until 4 weeks, whichever is earlier. | 1. Functional status  2. Blood composition  3. Standard anthropometrics  4. Grip strength  5. Rehabilitation outcomes (days to bear weight on fractured leg with assistance, days to mobilize independently without assistance)  6. Length of stay | Nil | ↔ biochemical parameters, grip strength, rehabilitation outcomes, length of stay or functional endpoints |
| Tidermark et al. [78], Tengstrand et al. [79], Carlsson et al. [80]  (To evaluate the effects of a protein-rich liquid supplementation,  alone or in combination with the anabolic steroid nandrolone decanoate, on body composition, activities of daily living (ADL) status and the health-related quality of  life (HRQoL) after a femoral neck fracture.) | 59; 82.9 | 1:1:1 | 1. PR (nutrition group) (*Inpatient to home*); protein-rich formula (Fortimel®, 200ml/day, 20g protein/day) for 6 months  2. PR/N (combined therapy) (*Inpatient to home*); protein-rich formula (Fortimel®, 200ml/day, 20g protein/day) and nandrolone decanoate (Deca-Durabol® 25mg i.m./3 weeks for 6 months. Intramuscular nandrolone injections were given by a research nurse in the home of the patients.  All patients received additional calcium (1g) and vitamin D (400IE) daily. | C (controls) (*Inpatient to home*); standard treatment, all patients received additional calcium (1g) and vitamin D (400IE) daily. | 1. Nutritional status  2. Functional assessment – activities of daily living (ADL) status and muscle function  3. Quality of life (EQ5D) | Nil | ↑ lean body mass, ADL status, quality of life, total body bone mineral density, protein intake without adversely affecting appetite |
| Unnanuntana et al. [90]  (To investigate the effect of bisphosphonate initiation on  short-term functional recovery in femoral neck fracture patients  at 2 versus 12 weeks after hemiarthroplasty.) | 100; 76.6 | 1:1 | 1. BIS initiation at week 2 (*Inpatient to home*); Risedronate 35mg/week at 2 weeks after surgery  2. BIS initiation at week 12 (*Home*); Risedronate 35mg/week at 12 weeks after surgery  All patients were instructed to take calcium (1000mg/day) and Vitamin D supplementation indefinitely. | Nil | Functional recovery – Barthel Index, EuroQol 5D, visual analog scale, 2-min walk test, and timed get-up-and-go test | Nil | ↔ short-term functional recovery  Suggested to correct low serum calcium and vitamin D status prior to or at the time of bisphosphonate initiation |
| Wyers et al. [83, 84]  (To evaluate  the effect of nutritional intervention on nutritional status, functional status, total length of stay, postoperative  complications and cost-effectiveness.) | 152; 76.5 | 1:1 | Intervention group (*Inpatient to home*); dietetic counselling and oral nutritional supplementation (ONS) for 3 months following surgery. Dietician had 10 contacts with each patient (2 during hospitalization and 8 thereafter), ONS comprise of 400ml and 40g per day of protein consumed in-between meals. Usual physical and exercise therapy daily during hospitalization and after discharge. | Control group (*Inpatient to home*); usual nutritional care – dietetic advice or ONS were only provided if prescribed by the medical doctor in charge. Usual physical and exercise therapy daily during hospitalization and after discharge. | Total length of stay (LOS) in hospital and rehabilitation clinics until 6 months postoperatively | 1. Nutritional outcomes like energy and nutrient intake, body weight and height, handgrip strength; body mass index  2. Functional outcomes  3. Postoperative complications (until 6 months)  4. Incidence of subsequent fractures  5. Mortality (until 5 years) | ↑ nutritional intake and status  ↔ LOS or clinical outcomes |
| **Optimizing Clinical Management** | | | | | | | |
| Dallimore et al. [97]  (To determine whether physiotherapy patient education, delivered using iPad applications, was effective in eliciting greater patient recall and satisfaction as compared to patient education delivered using a paper-based booklet, among a cohort of patients who had undergone hip surgery.) | 42; 70.5 | 8:13 | Intervention (*Inpatient*); received information on hip surgery physiotherapy via an iPad Air, where contexts were presented in a multimedia format, available in three languages. Intervention was carried out during patients’ first four 30-minute postoperative physiotherapy sessions. | Control (*Inpatient*); received information on hip surgery physiotherapy via a standard A5-sized paper booklet, written in English and had pictures to augment contents. Intervention was carried out during patients’ first four 30-minute postoperative physiotherapy sessions. | Patient satisfaction (of patient education received), Patient recall (of education content) | Nil. | ↑ patient satisfaction and recall of physiotherapy patient education |
| Houwing et al. [105]  (To investigate the effect of nutritional supplementation on incidence of pressure ulcers (PU) in hip-fracture patients at risk of  developing PU.) | 103; 81 | 1:1 | Supplement (*Inpatient*); high-protein nutritional supplement enriched with arginine, zinc and antioxidants dose of 400 ml/day, starting immediately postoperatively for a period of 4 weeks or until discharged. | Placebo (*Inpatient*); non-caloric, water-based supplement containing only sweeteners, colourants and flavourings dose of 400 ml/day for a period of 4 weeks or until discharged. | Development of pressure ulcers (PU) (incidence, incidence stage, first day of PU, no. of days of PU, total maximum wound size) | Nil. | ↔ outcomes but intervention possibly contributed to a delayed onset and progression of PU in patients with hip fractures at risk of developing PU |
| Jobory et al. [104]  (To compare two treatment regimes, one with and one without postoperative precautions in hemiarthroplasty patients, in terms of dislocation rate and patient-reported outcome.) | 394; 84.4 | 1:1 | Precaution group (*Inpatient*); standard postoperative hip precautions including limited flexion of the hip to 90° and limited adduction of the hip, mandatory assistive equipment to use for at least 3 months, and knee brace for up to 6 weeks. | Non-precaution group (*Inpatient*); no restrictions on mobility – encouraged to move freely during the recovery phase and assistive equipment were prescribed only if needed. | Dislocation rate | Patient reported outcome | Precautions not associated with the risk of dislocation when direct lateral approach is used  ↔ patient reported outcome |
| Leegwater et al. [158, 159]  (To evaluate the efficacy of  continuous -flow cryocompresssion (CFC) therapy on pain in the first 72 postoperative hours of hip fracture patients.) | 125; 78.6 | 1:1 | Continuous-flow cryocompression therapy (CFCT) (*Inpatient*); receive 10 – 12 CFCT of 30 min each during first 72h postoperative. | Control (*Inpatient*); only asked to value numeric rating scale (NRS) pain at rest. | Numeric rating scale (NRS) pain the first 72 postoperative hours. | Analgesic use; postoperative haemoglobin change and transfusion incidence; functional outcome; length of stay; delirium incidence; location of rehabilitation; patient-reported health outcome; complications and feasibility. | ↓ NRS pain at 72h  ↔ secondary outcomes |
| Lenze et al. [98]  (To test out Enhanced Medical Rehabilitation, an intervention designed to increase patient engagement in, and intensity of, daily physical and occupational therapy sessions in post-acute care rehabilitation.) | 26; 78.3 | 1:1 | EMR (Enhanced Medical Rehabilitation) (*Inpatient*); a set of behavioral skills for therapists to integrate into their OT/PT practice to increase the intensity of, and the patient’s engagement in all therapeutic sessions. | Standard-of-care therapy (*Inpatient*). | 1. Therapy intensity  2. Therapy engagement using the Rehabilitation Participation Scale  3. Functional and performance outcomes usin Barthel Index, gait speed and six-minute walk | Nil | ↑ therapy intensity, therapy engagement, gait speed, six-minute walk and Barthel Index score |
| Lundström et al. [101]  (To examine whether a postoperative  multi-factorial intervention program can reduce delirium and improve outcome in patients with  femoral neck fractures.) | 199; 82 | 1:1 | Intervention program (*Inpatient*); postoperative care in geriatric unit specializing in geriatric orthopedic patients. | Control group (*Inpatient*); postoperative care in the Orthopedic Department according to usual postoperative care routines. | Number of days of postoperative delirium | 1. Complications during hospitalization  2. Length of stay  3. In-hospital and one-year mortality | ↓ days of delirium, other complications and length of stay |
| Oude Voshaar et al. [99], Burns et al. [100]  (To examine the effect of pain, depression, cognitive impairment and fear of falling directly and 6 weeks after hip fracture surgery on functional outcome  at 6 months.) | 187; 79.8 | 1:1 | Intervention group (*Inpatient*):  1. Nurse-led intervention; 6 weekly sessions consisting of meetings with a psychiatric nurse from after surgery to discharge  2. Cognitive Behaviour Therapy (CBT) intervention; maximum of 7 sessions with an assistant psychologist. | Treatment-as-usual group (*Inpatient*). | Depressive  symptoms at 6 weeks, using  Geriatric Depression Scale and Hospital Anxiety and Depression Scale for mood | 1. Functional outcomes (up-and-go test, gait test and functional reach, impact of hip fracture of daily living)  2. Psychological outcomes (depression, pain, fear of falling) | ↔ depression, pain, functional recovery or length of stay  Fear of falling and cognitive functioning may be more important than pain and depression to predict functional recovery after hip fracture surgery |
| Papadopoulos et al. [102]  (To investigate whether postoperative ondansetron administration has a favorable effect on postoperative  delirium and 30th day cognitive function and pain in patients undergoing surgery with general anaesthesia due to  femoral or hip fracture.) | 106; 71.3 | 1:1 | Group A (ondansetron) (*Inpatient*); postoperatively 4ml of ondansetron (8mg) daily i.v. for 5 days. | Group B (placebo) (*Inpatient*); postoperatively 4ml of placebo daily i.v. for 5 days. | Postoperative delirium and 30^th^ day cognitive function | Postoperative pain | ↓ delirium, depression, pain  ↑ functionality, independence, cognitive status |
| Parkinson et al. [103]  (To reduce the prevalence and severity of post-surgery continence problems among patients, aged from 60-years, undergoing surgery for fractured neck of femur (#NOF), using a best practice “case-management model” multifactorial intervention.) | 45; 81 | 1:1 | Intervention (*Inpatient*); multifactorial best practice “case-management model” | Control (*Inpatient*); normal care | Incontinence severity after surgery, Incontinence Score was calculated from self-reported  patient bladder function information across twelve bladder related symptoms | Nil | ↔ incontinence status (small sample size)  A simple screening tool would ensure focused assessment and treatment for those most at risk of incontinence after fractured neck of femur, which would be more acceptable to staff and a more efficient use of resources. |
| **Prevention of venous thromboembolism (VTE)** | | | | | | | |
| Eriksson et al. [109], Dobesh [110] (To evaluate the benefit-to-risk ratio of a 2.5mg once-daily subcutaneous injection of fondaparinux sodium compared with placebo.) | 659; 79 (median) | 1:1 | Fondaparinux Sodium (*Inpatient to home*); subcutaneous injection of 2.5mg of fondaparinux sodium once-daily, for 25 – 31 days from day of surgery. | Placebo (*Inpatient to home*); subcutaneous injection of 2.5mg of fondaparinux sodium once-daily, for 6 – 8 days from day of surgery, followed by placebo up to 25 – 31 days. | 1. Venous thromboembolism (VTE), defined as deep vein thrombosis, pulmonary embolism, or both.  2. Major bleeding, including fatal bleeding, retroperitoneal, intracranial, or intraspinal bleeding, bleeding that involved any other critical organ, bleeding index of 2 or more. | 1. Total, proximal, and distal deep vein thrombosis, and symptomatic VTE.  2. Death, other bleeding, transfusion requirements, and any other adverse events. | ↓ risk of VTE by 96% (well tolerated without increasing the risk of major bleeding) |
| Eriksson et al. [160]  (To investigate the antithrombotic  potential of TTP889, which inhibits up to 90% of factor (F)IXa.) | 261; 73.5 | 1:1 | TTP889 (a small molecule that inhibits up to 90% of FIXa activity at therapeutic doses) (*Inpatient*); first dose of oral TTP889 300mg once daily, 12 – 48 h after standard prophylaxis, treatment administered each morning after food and continued for 3 weeks (± 2 days) until mandatory bilateral venography. | Placebo (*Inpatient*); first dose of placebo, 12 – 48 h after standard prophylaxis, treatment administered each morning after food and continued for 3 weeks (± 2 days) until mandatory bilateral venography. | 1. Total Venous thromboembolism (VTE) events (venographic or symptomatic DVT, or pulmonary embolism (PE)]  during treatment. | individual  components of DVT (total, proximal and distal), symptomatic  VTE, major VTE (a composite of proximal DVT and PE), VTE-related death, and all-cause mortality during treatment. | ↔ VTE events (lack of antithrombotic potential for TTP889 at this dose in this clinical setting) |
| Fisher et al. [106]  (To evaluate the efficacy and safety of extended thromboprophylaxis with the ultra-low molecular-weight heparin semuloparin compared with placebo in patients  undergoing hip fracture surgery.) | 469; 71 (median for intervention group) | 2:1 | Semuloparin (*Inpatient*); (run-in phase) once daily open-label semuloparin 20mg od subcutaneous injections for 7 – 10 days after surgery, first injection administered 8 hrs (±1) after surgery. Followed by subcutaneous injections of semuloparin (20mg, or 10mg if estimated CrCl of <30ml/min at randomization) for 19 – 23 days, first injection was administered ≥ 12 hrs, and no later than 24 hrs (± 2) after the last injection of the run-in phase. | Placebo (*Inpatient*); (run-in phase) once daily open-label semuloparin 20mg od subcutaneous injections for 7 – 10 days after surgery, first injection administered 8 hrs (±1) after surgery. Followed by subcutaneous injections of placebo for 19 – 23 days, first injection was administered ≥ 12 hrs, and no later than 24 hrs (± 2) after the last injection of the run-in phase. | Bleeding events,  blood transfusions, hemoglobin level, AEs and laboratory  data during the main safety analysis period (defined as the  period from the first injection of the study treatment of the  double-blind period until the last injection of the study  treatment of the double-blind period plus three calendar days). | Nil | ↓ Venous thromboembolism or all-cause death  Favorable benefit-to-risk profile of extended duration thromboprophylaxis with semuloparin after hip fracture surgery beyond the 7-10 days run-in period, and confirms the need for continuing thromboprophylaxis for ≥4 weeks |
| Fuji et al. [161]  (To investigate the safety and efficacy of edoxaban in Japanese patients  undergoing hip fracture surgery.) | 92; 76.1 | 2:1 | Edoxaban (*Inpatient*); 30mg once daily, initiated within 6 – 24 hours after surgery, treatment for 11 – 14 days. Concomitant use of mechanical physiotherapy (intermittent pneumatic compression or elastic stockings) was permitted. | Enoxaparin (*Inpatient*); 2000 IU sc every 12 hours, initiated within 24 – 36 hours after surgery, treatment for 11 – 14 days. Concomitant use of mechanical physiotherapy (intermittent pneumatic compression or elastic stockings) was permitted. | Incidence of major or clinically relevant non-major (CRNM) bleeding and the incidence of any bleeding event (major, CRNM, or minor bleeding) from the start of treatment to  completion day of treatment, inclusive. | Incidence of individual bleeding events, AEs, ADRs, vital  signs and laboratory test data. | ↔ major or CRNM bleeding events  ↑ incidence of any bleeding event (may be due to lower body weight) |
| Hamilton et al. [107]  (To evaluate the benefits and risks of prophylactic anticoagulants in patients after hip fracture surgery.) | 76; 77 | 1:1 | Anticoagulation group (*Inpatient*); Phenindione, an oral anticoagulant was administered as soon as subjects were able to swallow after operation. | Control group (*Inpatient*); surgeon was responsible to treat subjects who showed clinical or phlebographic evidence of venous thrombosis, usually by anticoagulation. | Prolongation of the prothrombin time to two to two and a half times the control | Nil | Early use of a prophylactic anticoagulant ↓ the incidence of venous thrombosis |
| Lassen et al. [162]  (To compare semuloparin and enoxaparin after  major orthopedic surgery.) | 987; 75.5 (SAVE-HIP2) | 1:1 | 1. Enoxaparin (*Inpatient*); 40 mg o.d., first dose given either preoperatively (12 ± 1h before surgery) or postoperatively (12 ± 1h after surgery) and administered for 7 – 10 days after surgery.  2. Semuloparin (*Inpatient*); 20 mg o.d., first dose given 8 ± 1h after surgery and administered for 7 – 10 days after surgery. | Nil | Composite of any deep vein thrombosis, non-fatal pulmonary embolism or all-cause death | Major bleeding, clinically relevant non-major (CRNM) bleeding, and any clinically relevant bleeding (major bleeding plus CRNM) | No superiority demonstrated in semuloparin  ↔ safety profile of both semuloparin and enoxaparin |
| Lowe et al. [108]  (To investigate the efficacy of postoperative subcutaneous ancrod in preventing deep vein thrombosis (D.V.T.) after hip fracture, and to determine whether preoperative plasma-fibrinogen and blood-viscosity are associated with D.V.T. in these patients.) | 110; 73.9 | 1:1 | Ancrod (*Inpatient*); subcutaneous injection with four one-ml ampoules of ancrod (‘Arvin’, 70units/ml) on return to the ward at the end of the operation. Another ampoule was injected subcutaneously on each of the next four mornings. | Saline (*Inpatient*); subcutaneous injection with four one-ml ampoules of saline (in identical ampoules) on return to the ward at the end of the operation. Another ampoule was injected subcutaneously on each of the next four mornings. | Deep vein thrombosis (D.V.T.), pulmonary embolism, bleeding, wound or fracture complications, and blood transfusion in the 10 days after surgery. | Late fracture complications detected at clinic follow-up 6 months after surgery. | ↓ incidence of D.V.T.  Study not large enough to determine outcome for incidence of pulmonary embolism |
| Tang et al. [111]  (To compare the efficacy and safety of Rivaroxaban alone, Enoxaparin alone and Enoxaparin followed by Rivaroxaban with regard to the anticoagulant therapy.) | 287; 69.7 | 1:1:1 | 1. The Rivaroxaban group (*Inpatient to outpatient*); orally administered a dosage of 10mg/d at 6h following operation, lasting for 28 consecutive days.  2. The low-molecular-weight heparin group (*Inpatient to outpatient*); administered subcutaneously with Enoxaparin at a dosage of 4000IU/d, 12h following operation, lasting for 28 consecutive days.  3. The sequential therapy group (*Inpatient to outpatient*); Enoxaparin at a dosage of 4000IU/d 12h following the surgery for 1w, and then with oral Rivaroxaban at 10mg 1time/d which lasted for 28 consecutive days. | Nil | Incidence of postoperative VTE | 1. Compliance  2. Treatment costs  3. Incidences of adverse effects namely, bleeding and wound complications | ↓ incidence of VTE, postoperative drainage, treatment cost; ↑ patient compliance in the sequential therapy group compared to the Rivaroxaban group |
| **Others – group learning / motivational interviewing** | | | | | | | |
| Elinge et al. [139]  (To examine whether a group learning programme would influence the participants’  perceived activity performance and ability to participate in social life after a hip fracture.) | 35;73.4 | 3:2 | Group learning programme (*Outpatient*); led by a geriatric team (consisting of a dietitian, an occupational therapist, a physician, a physiotherapist and a social worker) in groups of 5 to 8, participants met for two hours weekly for 10 weeks, one hour of education on osteoporosis and falls prevention, and one hour of physical training with weight-bearing exercises. Participants also received an individually tailored home training programme. | Control (*Outpatient*); no intervention | Self-perceived ability to perform activities of daily living (Barthel ADL index), ability to participate in social life after a hip fracture | Participant’s activity preference | ↑ ability to resume meaningful participation in social life  ↓ difficulties in performing personal ADL |
| O'Halloran et al. [140]  (To investigate if motivational interviewing improved physical activity, self-efficacy, quality of  life, mobility and mental health in people living in the community after hip fracture.) | 25; 82.6 | 1:1 | Motivational interviewing (*Home*); usual care with telephone based motivational interviewing provided in 8 30-minute sessions delivered weekly by a physiotherapist. | Usual care (*Home*); follow-up with general practitioner or a community physiotherapist when required. | Physical activity levels as measured by an accelerometer | 1. Self-efficacy  2.Health-related quality of life  3. Mobility  4. Mental health | ↑ physical activity, self-efficacy, health-related quality of life  ↓ anxiety and depression |

**Supplemental table 4**: List of studies excluded during full text screening

| **Title** | **Reason for exclusion** |
| --- | --- |
| Prospective randomized controlled trial on the effect of fondaparinux sodium for prevention of venous thromboembolism after hip fracture surgery | Inappropriate study design |
| Pneumatic wound compression after hip fracture surgery did not reduce postoperative blood transfusion: A randomized controlled trial involving 292 fractures | Inappropriate intervention |
| Effect of alendronate in elderly patients after low trauma hip fracture repair | Jadad Score < 3 |
| Facilitated bone mineral density testing versus hospital-based case management to improve osteoporosis treatment for hip fracture patients: additional results from a randomized trial | Inappropriate study design |
| To evaluate the effectiveness of a discharge-planning programme for hip fracture patients | Jadad Score < 3 |
| Does nutritional intervention for patients with hip fractures reduce postoperative complications and improve rehabilitation? | Inappropriate study design |
| Home rehabilitation after hip fracture. A randomized controlled study on balance confidence, physical function and everyday activities | Inappropriate study design |
| Long-term effects of home rehabilitation after hip fracture - 1-year follow-up of functioning, balance confidence, and health-related quality of life in elderly people | Inappropriate study design |
| Orthopaedic management improves the rate of early osteoporosis treatment after hip fracture. A randomized clinical trial | Inappropriate study design |
| A single home visit by an occupational therapist reduces the risk of falling after hip fracture in elderly women: a quasi-randomized controlled trial | Inappropriate study design |
| Adherence to recommendations for fall prevention significantly affects the risk of falling after hip fracture: post-hoc analyses of a quasi-randomized controlled trial | Inappropriate study design |
| Comparison of daily, weekly, and monthly vitamin D3 in ethanol dosing protocols for two months in elderly hip fracture patients | Inappropriate study design |
| Individual nutrition therapy and exercise regime: a controlled trial of injured, vulnerable elderly (INTERACTIVE trial) | Inappropriate study design |
| Cost-effectiveness of individualized nutrition and exercise therapy for rehabilitation following hip fracture | Inappropriate study design |
| Potential mediators of the mortality reduction with zoledronic acid after hip fracture | Inappropriate study design |
| Treatment and prevention of depression after surgery for hip fracture in older people: cost-effectiveness analysis | Inappropriate study design |
| Low back pain after unstable extracapsular hip fractures: randomized control trial on a specific training | Inappropriate patient population |
| Tape blisters after hip surgery: can they be eliminated completely? | Inappropriate intervention |
| The use of graduated compression stockings in association with fondaparinux in surgery of the hip. A multicentre, multinational, randomised, open-label, parallel-group comparative study | Inappropriate intervention |
| A comparative study of organized class-based exercise programs versus individual home-based exercise programs for elderly patients following hip surgery | Inappropriate study design |
| Efficacy of a comprehensive geriatric intervention in older patients hospitalized for hip fracture: a randomized, controlled trial | Inappropriate intervention |
| A randomized clinical trial of the effectiveness of a discharge planning intervention in hospitalized elders with hip fracture due to falling | Inappropriate intervention |
| Effects on function and quality of life of postoperative home-based physical therapy for patients with hip fracture | Inappropriate study design |
| A pilot investigation of the short-term effects of an interdisciplinary intervention program on elderly patients with hip fracture in Taiwan | Inappropriate intervention |
| Interdisciplinary intervention decreases cognitive impairment for older Taiwanese with hip fracture: 2-year follow-up | Inappropriate study design |
| Subtrochanteric fractures in bisphosphonate-naive patients: results from the HORIZON-recurrent fracture trial | Inappropriate study design |
| Nightly enteral nutrition support of elderly hip fracture patients: a pilot study | Jadad Score < 3 |
| The effects of calcitonin on acute bone loss after pertrochanteric fractures. A prospective, randomised trial | Inappropriate intervention |
| Nutritional supplements after hip fracture: poor compliance limits effectiveness | Jadad Score < 3 |
| Effect of a video intervention on functional recovery following hip replacement and hip fracture repair | No access to full article |
| Intensive physical therapy after hip fracture. A randomised clinical trial | No access to full article |
| Intensive physical training in geriatric patients after severe falls and hip surgery | Jadad Score < 3 |
| Reducing delirium after hip fracture: a randomized trial | Inappropriate intervention |
| Clinical pathway for fractured neck of femur: a prospective, controlled study | Inappropriate intervention |
| Deep vein thrombosis prophylaxis in hip fractures: a comparison of the arteriovenous impulse system and aspirin | No access to full article |
| The effect of 25-dihydroxyvitamin D on the bone mineral metabolism of elderly women with hip fracture | Jadad Score < 3 |
| Pressure sores and tube feeding in patients with a fracture of the hip: a randomized clinical trial | Jadad Score < 3 |
| Nightly enteral nutrition support of elderly hip fracture patients: a phase I trial | Jadad Score < 3 |
| The management of elderly patients with femoral fractures. A randomised controlled trial of early intervention versus standard care | Inappropriate intervention |
| Comparison of the use of a foot pump with the use of low-molecular-weight heparin for the prevention of deep-vein thrombosis after total hip replacement. A prospective, randomized trial | Inappropriate intervention |
| Systematic home-based physical and functional therapy for older persons after hip fracture | Inappropriate study design |
| Randomized trial comparing early postoperative irradiation vs. the use of nonsteroidal antiinflammatory drugs for prevention of heterotopic ossification following prosthetic total hip replacement | Inappropriate patient population |
| Benefits of supplementary tube feeding after fractured neck of femur: a randomised controlled trial. 1983 | Jadad Score < 3 |
| Plastic adhesive drapes and wound infection after hip fracture surgery | Jadad Score < 3 |
| The A-V Impulse System reduces deep-vein thrombosis and swelling after hemiarthroplasty for hip fracture | Jadad Score < 3 |
| Postoperative mental impairment in hip fracture patients. A randomized study of reorientation measures in 223 patients | Inappropriate intervention |
| Subcutaneous ancrod in prevention of deep vein thrombosis after hip replacement surgery | Inappropriate patient population |
| A multi-component cognitive behavioural intervention for the treatment of fear of falling after hip fracture (FIT-HIP): protocol of a randomised controlled trial | Inappropriate study design |
| A trial assessing N-3 as treatment for injury-induced cachexia (ATLANTIC trial): does a moderate dose fish oil intervention improve outcomes in older adults recovering from hip fracture? | Inappropriate study design |
| Alendronate and raloxifene therapy in the early period after hip fracture | Jadad Score < 3 |
| An interdisciplinary intervention for older Taiwanese patients after surgery for hip fracture improves health-related quality of life | Inappropriate intervention |
| Blood transfusion and overall quality of life after hip fracture in frail elderly patients--the transfusion requirements in frail elderly randomized controlled trial | Inappropriate intervention |
| Blood transfusion and risk of infection in frail elderly after hip fracture surgery: the TRIFE randomized controlled trial | Inappropriate intervention |
| Blood transfusion strategy and risk of postoperative delirium in nursing homes residents with hip fracture. A post hoc analysis based on the TRIFE randomized controlled trial | Inappropriate intervention |
| Comparative effectiveness of fragility fracture integrated rehabilitation management for elderly individuals after hip fracture surgery: A study protocol for a multicenter randomized controlled trial | Inappropriate study design |
| COMplex Fracture Orthopedic Rehabilitation (COMFORT) - Real-time visual biofeedback on weight bearing versus standard training methods in the treatment of proximal femur fractures in the elderly: study protocol for a multicenter randomized controlled trial | Inappropriate study design |
| Comprehensive and subacute care interventions improve health-related quality of life for older patients after surgery for hip fracture: a randomised controlled trial | Inappropriate intervention |
| Comprehensive care improves health outcomes among elderly Taiwanese patients with hip fracture | Inappropriate intervention |
| Trajectories of Nutritional Status and Cognitive Impairment among Older Taiwanese with Hip Fracture | Inappropriate intervention |
| Two-year effects of an interdisciplinary intervention on recovery following hip fracture in older Taiwanese with cognitive impairment | Inappropriate intervention |
| Two-year effects of interdisciplinary intervention for hip fracture in older Taiwanese | Inappropriate intervention |
| Comprehensive care improves physical recovery of hip-fractured elderly Taiwanese patients with poor nutritional status | Inappropriate intervention |
| Comprehensive geriatric care for patients with hip fractures: a prospective, randomised, controlled trial | Inappropriate intervention |
| Effects of interventions on trajectories of health-related quality of life among older patients with hip fracture: a prospective randomized controlled trial | Inappropriate intervention |
| Continuous femoral nerve block versus fascia iliaca compartment block as postoperative analgesia in patients with hip fracture | Inappropriate intervention |
| Cranberry juice concentrate does not significantly decrease the incidence of acquired bacteriuria in female hip fracture patients receiving urine catheter: a double-blind randomized trial | Inappropriate intervention |
| Delirium outcomes in a randomized trial of blood transfusion thresholds in hospitalized older adults with hip fracture | Inappropriate intervention |
| Early serum IGF-I response to oral protein supplements in elderly women with a recent hip fracture | Inappropriate patient population |
| Effect of in-hospital comprehensive geriatric assessment (CGA) in older people with hip fracture. The protocol of the Trondheim Hip Fracture trial | Inappropriate study design |
| Physical behavior and function early after hip fracture surgery in patients receiving comprehensive geriatric care or orthopedic care--a randomized controlled trial | Inappropriate intervention |
| Who benefits from orthogeriatric treatment? Results from the Trondheim hip-fracture trial | Inappropriate intervention |
| Effect of melatonin on incidence of delirium among patients with hip fracture: a multicentre, double-blind randomized controlled trial | Inappropriate intervention |
| Effectiveness of a physical exercise intervention program in improving functional mobility in older adults after hip fracture in later stage rehabilitation: protocol of a randomized clinical trial (REATIVE Study) | Inappropriate study design |
| Effectiveness of task specific gait and balance exercise 4 months after hip fracture: protocol of a randomized controlled trial--the Eva-hip study | Inappropriate study design |
| Effectiveness of the computerized balance rehabilitation after hip fracture surgery: A study protocol of a prospective and open-label clinical trial | Inappropriate study design |
| Effects of 12-month home-based physiotherapy on duration of living at home and functional capacity among older persons with signs of frailty or with a recent hip fracture - protocol of a randomized controlled trial (HIPFRA study) | Inappropriate study design |
| Promoting mobility after hip fracture (ProMo): study protocol and selected baseline results of a year-long randomized controlled trial among community-dwelling older people | Inappropriate study design |
| Effects of ulinastatin on inflammatory response and cognitive function after hip arthroplasty for the elderly patients with femoral neck fracture | Jadad Score < 3 |
| Effects of Zoledronate on Mortality and Morbidity after Surgical Treatment of Hip Fractures | Inappropriate study design |
| Efficacy of ultrasound-guided fascia iliaca compartment block after hip hemiarthroplasty: A prospective, randomized trial | Inappropriate intervention |
| Enhanced interdisciplinary care improves self-care ability and decreases emergency department visits for older Taiwanese patients over 2 years after hip-fracture surgery: A randomised controlled trial | Inappropriate intervention |
| Evaluation of patient-centered rehabilitation model targeting older persons with a hip fracture, including those with cognitive impairment | Inappropriate study design |
| Functional recovery of older hip-fracture patients after interdisciplinary intervention follows three distinct trajectories | Inappropriate intervention |
| Hip and pelvic fracture patients with fear of falling: development and description of the "Step by Step" treatment protocol | Inappropriate study design |
| Hydrotherapy after total hip arthroplasty: a follow-up study | Inappropriate patient population |
| Improving community ambulation after hip fracture: protocol for a randomised, controlled trial | Inappropriate study design |
| Integrated care pathway for hip fractures in a subacute rehabilitation setting | Inappropriate study design |
| Lateral femoral cutaneous nerve block after total hip arthroplasty: a randomised trial | Inappropriate patient population |
| Liberal or restrictive transfusion in high-risk patients after hip surgery | Inappropriate intervention |
| Liberal versus restrictive blood transfusion strategy: 3-year survival and cause of death results from the FOCUS randomised controlled trial | Inappropriate intervention |
| Patient safety in elderly hip fracture patients: design of a randomised controlled trial | Inappropriate study design |
| PERFECTED enhanced recovery (PERFECT-ER) care versus standard acute care for patients admitted to acute settings with hip fracture identified as experiencing confusion: study protocol for a feasibility cluster randomized controlled trial | Inappropriate study design |
| Pilot randomized trial of donepezil hydrochloride for delirium after hip fracture | Inappropriate intervention |
| Postoperative blood transfusion strategy in frail, anemic elderly patients with hip fracture: the TRIFE randomized controlled trial | Inappropriate intervention |
| Postoperative red blood cell transfusion strategy in frail anemic elderly with hip fracture. A randomized controlled trial | Inappropriate intervention |
| Randomised trial of blood transfusion versus a restrictive transfusion policy after hip fracture surgery | Inappropriate intervention |
| Salmon calcitonin (Miacalcic ns 200 IU) in prevention of bone loss after hip replacement | Inappropriate study design |
| The administration of intermittent parathyroid hormone affects functional recovery from pertrochanteric fractured neck of femur: a protocol for a prospective mixed method pilot study with randomisation of treatment allocation and blinded assessment (FRACTT) | Inappropriate study design |
| The administration of intermittent parathyroid hormone affects functional recovery from trochanteric fractured neck of femur: a randomised prospective mixed method pilot study | Inappropriate study design |
| The effect of weekly risedronate on periprosthetic bone resorption following total hip arthroplasty: a randomized, double-blind, placebo-controlled trial | Inappropriate patient population |
| The effects of habitual functional training on physical functioning in patients after hip fracture: the protocol of the HIPFRAC study | Inappropriate study design |
| Translating Delirium Prevention Strategies for Elderly Adults with Hip Fracture into Routine Clinical Care: A Pragmatic Clinical Trial | Inappropriate study design |
| Developing a multidisciplinary rehabilitation package following hip fracture and testing in a randomised feasibility study: Fracture in the Elderly Multidisciplinary Rehabilitation (FEMuR) | Inappropriate study design |
| Enhancement of hip fracture healing in the elderly: Evidence deriving from a pilot randomized trial | Inappropriate study design |
| Fracture in the Elderly Multidisciplinary Rehabilitation (FEMuR): a phase II randomised feasibility study of a multidisciplinary rehabilitation package following hip fracture | Duplicate |
| Fracture in the Elderly Multidisciplinary Rehabilitation (FEMuR): study protocol for a phase II randomised feasibility study of a multidisciplinary rehabilitation package following hip fracture [ISRCTN22464643] | Duplicate |
| The effects of melatonin versus placebo on delirium in hip fracture patients: study protocol of a randomised, placebo-controlled, double blind trial | Inappropriate study design |
| Effectiveness of a tele-rehabilitation intervention to improve performance and reduce morbidity for people post hip fracture - study protocol for a randomized controlled trial | Inappropriate study design |
| The application of "upper-body yoga" in elderly patients with acute hip fracture: a prospective, randomized, and single-blind study | Inappropriate study design |
| Influence of postoperative analgesia on systemic inflammatory response and postoperative cognitive dysfunction after femoral fractures surgery: a randomized controlled trial | No access to full article |
| Prevention of postoperative bleeding in hip fractures treated with prosthetic replacement: efficacy and safety of fibrin sealant and tranexamic acid. A randomised controlled clinical trial (TRANEXFER study) | Inappropriate intervention |
| Multifactorial intervention for hip and pelvic fracture patients with mild to moderate cognitive impairment: study protocol of a dual-centre randomised controlled trial (OF-CARE) | Inappropriate study design |
| Should we provide outreach rehabilitation to very old people living in Nursing Care Facilities after a hip fracture? A randomised controlled trial | Inappropriate intervention |
| Adherence to Home-Based Rehabilitation in Older Adults With Diabetes After Hip Fracture | Inappropriate intervention |
| Effects of nutritional intervention upon bone turnover in elderly hip fracture patients. Randomized controlled trial | Inappropriate intervention |
| Supplemented amino acids may enhance the walking recovery of elderly subjects after hip fracture surgery | Inappropriate study design |
| Prospective randomised study of an orthopaedic geriatric inpatient service | Jadad Score < 3 |
| Benefits of supplementary tube feeding after fractured neck of femur: a randomised controlled trial | Inappropriate study design |
| A comparison of the effects of three oral bisphosphonates on the peripheral skeleton in postmenopausal osteoporosis: The trio study | Inappropriate patient population |
| A critical appraisal of bleeding events reported in venous thromboembolism prevention trials of patients undergoing hip and knee arthroplasty | Inappropriate study design |
| A liberal blood transfusion strategy after hip fracture surgery does not increase the risk of infection in frail elderly | Inappropriate intervention |
| A liberal blood transfusion strategy improves survival in nursing home residents with hip fracture | Inappropriate intervention |
| A model of BMD changes after alendronate discontinuation to guide postalendronate BMD monitoring | Inappropriate intervention |
| A novel delayed-release risedronate 35 mg once-a-week formulation taken with or without breakfast: 2-year BMD data | Abstract only |
| A phase iib study of MK-5442 calcium sensing receptor (CASR) antagonist in bisphosphonate-treated patients | No access to full article |
| A randomised, double-blinded clinical study on the efficacy of multimedia presentation using an iPad for patient education of postoperative hip surgery patients in a public hospital in Singapore | Duplicate |
| A randomized alendronate-controlled trial of romosozumab: Results of the phase 3 active-controlled fracture study in postmenopausal women with osteoporosis at high risk | Abstract only |
| A randomized alendronate-controlled trial of romosozumab: Results of the phase 3 ARCH Study (Active-contRolled fraCture study in postmenopausal women with osteoporosis at High risk) | Duplicate |
| A randomized controlled trial of early initiation of osteoporosis assessment and management in the acute setting of the fracture clinic | No access to full article |
| A randomized controlled trial to evaluate intensity of community-based rehabilitation provision following stroke or hip fracture in old age: Results at 12-month followup | Inappropriate patient population |
| A randomized open-label study to evaluate the safety and efficacy of denosumab and ibandronate in postmenopausal women sub-optimally treated with daily or weekly bisphosphonates | Abstract only |
| A randomized, double-blind, controlled trial of melatonin versus placebo in delirium | Abstract only |
| A randomized, double-blind, placebo-controlled clinical trial on the treatment of vitamin D insufficiency in postmenopausal women | Inappropriate patient population |
| A single bisphosphonate infusion does not accelerate fracture healing in high tibial osteotomies | Inappropriate patient population |
| A systematic review and adjusted indirect comparison of oral anticoagulants | Inappropriate study design |
| A systematic review and meta-analysis of proportions of thrombosis and bleeding in patients receiving venous thromboembolism (VTE) prophylaxis after orthopedic surgery (OS). an update | Abstract only |
| Abaloparatide significantly reduces vertebral and nonvertebral fractures and increases BMD regardless of baseline risk | Abstract only |
| Action observation treatment in the rehabilitation of post-surgical orthopaedic patients: A randomised controlled trial | Abstract only |
| Adherence to nutrition supplements among patients with a fall-related lower limb fracture | No access to full article |
| Advancement of physical process by mental activation: A prospective controlled study | Inappropriate patient population |
| Against all odds: Results from the zest trial in long term care residents | Duplicate |
| Against all odds: Results from the zest trial in long term care residents | No access to full article |
| Alendronate sodium/vitamin D(3) combination tablet versus calcitriol for osteoporosis in chinese postmenopausal women: A 6-month, randomized, open-label, active-comparator controlled study with a 6-month extension phase | Inappropriate patient population |
| Alfacalcidol in men with osteoporosis: A prospective, observational, 2-year trial on 214 patients | Inappropriate patient population |
| An intervention study exploring the effects of providing older adult hip fracture patients with an information booklet in the early postoperative period | Duplicate |
| An intervention study exploring the effects of providing older adult hip fracture patients with an information booklet in the early postoperative period | Inappropriate study design |
| An RCT to determine the effect of a heel elevation device in pressure ulcer prevention post-hip fracture | Inappropriate intervention |
| Anti-fracture efficacy of zoledronate in subgroups of osteopenic postmenopausal women: secondary analysis of a randomized controlled trial | Inappropriate patient population |
| Aspirin versus anticoagulation for prevention of venous thromboembolism major lower extremity orthopedic surgery: a systematic review and meta-analysis | Inappropriate study design |
| Aspirin versus anticoagulation for prevention of venous thromboembolism major lower extremity orthopedic surgery: A systematic review and meta-analysis | Duplicate |
| Aspirin versus anticoagulation for the prevention of venous thromboembolism in orthopedic patients after lower extremity reconstructive surgery - A systematic review | Inappropriate study design |
| Assessment of Nutrition and Supplementation in Patients With Hip Fractures | Inappropriate intervention |
| Association between fermented milk product intake and bone health in postmenopausal women: A systematic review | Inappropriate patient population |
| Back to the future – Feasibility of recruitment and retention to patient education and telephone follow-up after hip fracture: A pilot randomized controlled trial | Inappropriate study design |
| Benefit-to-risk profile of the ultra-low-molecularweight heparin (ULMWH) semuloparin for prevention of venous thromboembolism (vte): A meta-analysis of 3 major orthopaedic surgery studies | Inappropriate study design |
| Better efficacy of minodronic acid than raloxifene chloride observed in postmenopausal women with multicenter, open-label randomized controlled head-to-head trial, japanese osteoporosis intervention trial (JOINT-04) | Inappropriate patient population |
| Bisphosphonate use in women and men who are at high risk for new fractures and living in long-term care homes: The vitamin D osteoporosis study (ViDOS) | Abstract only |
| Bisphosphonates for periprosthetic bone loss after joint arthroplasty: A meta-analysis of 14 randomized controlled trials | Inappropriate study design |
| Bisphosphonates for Secondary Prevention of Osteoporotic Fractures: A Bayesian Network Meta-Analysis of Randomized Controlled Trials | Inappropriate study design |
| Bisphosphonates reduce fracture risk in postmenopausal women with diabetes: Results from FIT and HORIZON trials | Abstract only |
| Blood transfusion and delirium at 45-days post-hip fracture: Longterm outcomes from the focus cognitive ancillary study | Inappropriate intervention |
| Blood transfusion strategies in recovery from physical disability and overall quality of life in frail elderly with hip fracture | Inappropriate intervention |
| Blood transfusion strategy and risk of postoperative delirium in nursing homes residents with hip fracture | Inappropriate intervention |
| BMD after hip fractures: Response to annual i.v. Zoledronic acid 5 mg | Abstract only |
| Bmd response to a novel delayed-release risedronate 35 mg once-a-week formulation taken with or without breakfast: One year results | Abstract only |
| Bone mineral density increases with monthly I.V. ibandronate injections contribute to its fracture risk reduction in primary osteoporosis: 3-year analysis of the phase III mover study | Duplicate |
| Bone mineral density increases with monthly i.v. ibandronate injections contribute to its fracture risk reduction in primary osteoporosis: 3-year analysis of the phase III mover study | No access to full article |
| Bone safety of a novel delayed-release risedronate 35 mg once-a-week assessed by bone histology and histomorphometry | Inappropriate patient population |
| Calcium plus vitamin D supplementation and risk of fractures: an updated meta-analysis from the National Osteoporosis Foundation | Inappropriate patient population |
| Cholinergic enhancers for preventing postoperative delirium among elderly patients after hip fracture surgery: A meta-analysis | Inappropriate intervention |
| Clinical and economic consequences of using fondaparinux or enoxaparin for the prevention of venous thromboembolism in hip surgery in brazil | Abstract only |
| Clinical and radiological outcome of total hip replacement five years after pamidronate therapy | Inappropriate patient population |
| Clinical comparison of efficacy and safety of two teriparatide formulations: Osteofortil® and forteo® | Abstract only |
| Clinical efficacy of monthly I.V. Ibandronate vs. Daily oral risedronate in patients with primary osteoporosis: The phase III mover study | Abstract only |
| Clinical impact of bleeding complications with direct oral anticoagulants for the prevention of venous thromboembolism in orthopaedic surgery: A systematic review and meta-analysis of randomized controlled trials | Abstract only |
| Clinical significance of multidisciplinary team collaboration for the treatment of hip fractures in the elderly | Abstract only |
| Clinical trials express: Fracture risk reduction with denosumab in Japanese postmenopausal women and men with osteoporosis: Denosumab Fracture Intervention Randomized Placebo Controlled Trial (DIRECT) | Inappropriate intervention |
| Closing the gap in postfracture care at the population level: A randomized controlled trial | Inappropriate patient population |
| Closing the Postfracture Care Gap Using Administrative Health Databases: Design and Implementation of a Randomized Controlled Trial | Inappropriate patient population |
| Combination ofteriparatide and deno-sumab accelerate proximal femur fracture healing | Abstract only |
| Combination therapies for the treatment of osteoporotic fractures are not created equal: A network meta-analysis study | Abstract only |
| Combined (mechanical and pharmacological) modalities for the prevention of venous thromboembolism in joint replacement surgery | Inappropriate patient population |
| Comparative effectiveness of Low-Molecular-Weight Heparins versus other anticoagulants in major orthopedic surgery: A systematic review and meta-analysis | Inappropriate study design |
| Comparative treatment outcomes in patients with prior fracture previously treated with a bisphosphonate: Results from the denosumab/ibandronate and denosumab/risedronate trials | Inappropriate patient population |
| Comparison of 3,000 and 5,000 IU aXa/day certoparin in the prevention of deep-vein thrombosis after total hip replacement | Inappropriate intervention |
| Comparison of mean pain score by applying with and without skin traction in patients with hip fracture | Inappropriate intervention |
| Comparison of the effects of three oral bisphosphonate therapies on the peripheral skeleton in postmenopausal osteoporosis: the TRIO study | Inappropriate patient population |
| Comparison of the Efficacy and Safety of Aspirin and Rivaroxaban Following Enoxaparin Treatment for Prevention of Venous Thromboembolism after Hip Fracture Surgery | Inappropriate study design |
| Comparison of the postoperative analgesic efficacies of intravenous acetaminophen and fascia iliaca compartment block in hip fracture surgery: A randomised controlled trial | Inappropriate intervention |
| Comprehensive geriatric assessment for prevention of delirium post hip fracture: A systematic review of randomised controlled trials | Duplicate |
| Comprehensive geriatric assessment for prevention of delirium post hip fracture: A systematic review of randomised controlled trials | No access to full article |
| Continued fracture risk reduction after 12 months of romosozumab followed by denosumab through 36 months in the phase 3 FRAME (FRActure study in postmenopausal woMen with ostEoporosis) Extension | Abstract only |
| CORR Insights(®): Does Teriparatide Improve Femoral Neck Fracture Healing: Results From A Randomized Placebo-controlled Trial | Inappropriate study design |
| Correlates of fear of falling and falls efficacy in geriatric patients recovering from hip/pelvic fracture | Inappropriate study design |
| Correlations between 25(OH)D and BMD change in postmenopausal osteoporotic women and other secondary analyses of a 1-year trial of weekly alendronate (ALN) plus vitamin D 5600 IU vs. Standard care | Duplicate |
| Correlations between 25(OH)D and bmd change in postmenopausal osteoporotic women: Secondary analyses of a 1-year trial of weekly alendronate (ALN) plus vitamin D(3) 5600 iu vs. standard care | Abstract only |
| Cost-effectiveness of nutritional intervention in hip fracture patients: A multi-centre randomised controlled trial (RCT) | Duplicate |
| Effect of nutritional intervention on length of stay, postoperative complications, functional status and mortality in hip fracture patients: A multi-centre randomised controlled trial (RCT) | Duplicate |
| Sun integrated nutritional intervention in the elderly after hip fracture. A process evaluation | Duplicate |
| Effect of nutritional intervention on nutritional intake and status in hip fracture patients: A multicentre randomised controlled trial (RCT) | Abstract only |
| Cost-effectiveness of two inexpensive postfracture osteoporosis interventions: Results of a randomized trial | Inappropriate patient population |
| Denosumab administration is not associated with fracture healing complications in postmenopausal women with osteoporosis: Results from the freedom trial | Abstract only |
| Denosumab compared with risedronate in glucocorticoid-treated subjects: Results from the final 24-month analysis of a randomized, double-blind, double-dummy study | Abstract only |
| Denosumab for the treatment of men with low bone mineral density | Abstract only |
| Denosumab for the treatment of men with low bone mineral density: 24-month results from the adamo trial | Abstract only |
| Denosumab leads to significantly greater increases in bone mineral density than ibandronate and risedronate in postmenopausal women at high risk for fracture who were previously treated with an oral bisphosphonate | Abstract only |
| Denosumab reduced osteoporotic fractures in postmenopausal women with osteoporosis with prior fracture: Results from freedom | Abstract only |
| Denosumab treatment in postmenopausal women with osteoporosis does not interfere with fracture-healing: Results from the FREEDOM trial | Inappropriate patient population |
| Denosumab treatment in postmenopausal women with osteoporosis for up to 9 years: Results through year 6 of the freedom extension | Abstract only |
| Do Immediate Postoperative Radiographs Change Patient Management after Fracture Fixation? A Systematic Review | Inappropriate study design |
| Does pulse lavage reduce hip hemiarthroplasty infection rates? | Inappropriate intervention |
| Drug prescribing in the elderly hip fracture patient-results from the Trondheim Hip Fracture Trial | Abstract only |
| Drug prevention aganist periprosthetic osteolysis 5 years after total hip arthroplasty: A controlled randomized evaluation | Non-English |
| Effect of 3 year Denosumab Treatment on Hip Structure in Japanese Postmenopausal Women and Men with Osteoporosis | Abstract only |
| Effect of alendronate on radiographic fracture healing after surgery for low-energy distal radius fractures | Abstract only |
| Effect of calcitonin on postoperative parameters in patients with hip fractures: Focus on the patients' ADL and QOL | Abstract only |
| Effect of Calcium β-Hydroxy-β-Methylbutyrate (CaHMB), Vitamin D, and Protein Supplementation on Postoperative Immobilization in Malnourished Older Adult Patients with Hip Fracture | No access to full article |
| Effect of haloperidol dosing frequencies on the duration and severity of delirium in elderly hip fracture patients. A prospective randomized trial | Abstract only |
| Effect of HBM rehabilitation exercises on depression, anxiety and health belief in elderly patients with osteoporotic fracture | Inappropriate intervention |
| Effect of humor on pain of patient with femur fracture | Abstract only |
| Effect of intravenous methylprednisolone in prevention of arterial hypoxemia due to fat embolism syndrome in patients with long bone fractures of lower limb - A double blind randomized trial | Inappropriate patient population |
| Effect of once-yearly i.v. zoledronic acid in men after hip fracture: Results from the horizon-recurrent fracture trial | Abstract only |
| Effect of once-yearly zoledronic acid in men after recent hip fracture: Results from horizon recurrent fracture trial | Abstract only |
| Effect of once-yearly zoledronic acid in men after recent hip fracture: Results from horizon recurrent fracture trial | Abstract only |
| Effect of osteoporosis medications on refracture and mortality following hip fracture surgery in postmenopausal women: A prospective randomized trial | Abstract only |
| Effect of reversal of neuromuscular blockade with sugammadex versus usual care on bleeding risk in a randomized study of surgical patients | Inappropriate patient population |
| Effect of structured exercise interventions in the early phase to improve physical functioning after hip fracture: A systematic review and meta-analysis | Abstract only |
| Effect of teriparatide compared with risedronate on back pain and incident vertebral fractures in postmenopausal women with osteoporotic vertebral fractures | Abstract only |
| Effect of teriparatide compared with risedronate on reduction of back pain and new vertebral fractures in postmenopausal women with osteoporotic vertebral fractures | Abstract only |
| Effect of teriparatide on bone mineral density and fracture in postmenopausal osteoporosis: Meta-analysis of randomised controlled trials | Inappropriate intervention |
| Effect of teriparatide or risedronate in bmd and fracture recovery in elderly patients with a recent pertrochanteric hip fracture: Final results of a 78-week randomized clinical trial | Abstract only |
| Effect of teriparatide or risedronate on pertrochanteric hip fractures recovery: 26-week results of a randomized clinical trial | Abstract only |
| Effect of the cathepsin K inhibitor, ONO-5334, on biochemical markers of bone turnover in the treatment of postmenopausal osteopenia or osteoporosis | Abstract only |
| Effect of the cathepsin k inhibitor, ONO-5334, on biochemical markers of bone turnover in the treatment of postmenopausal osteopenia or osteoporosis: 2-year results from the ocean study | Abstract only |
| Effectiveness of follow-up telephone calls to improve vitamin D+/-calcium compliance in elderly patients with hip fracture. A randomized study | Abstract only |
| Effectiveness of multicomponent interventions on incidence of delirium in hospitalized older patients with hip fracture: A systematic review | Inappropriate intervention |
| Effectiveness of Negative-Pressure wound Therapy following total Hip and Knee replacements | No access to full article |
| Effectiveness of oral nutritional supplementation for older women after a fracture: rationale, design and study of the feasibility of a randomized controlled study. | Inappropriate patient population |
| Effectiveness of sensor monitoring in a rehabilitation program for older patients after hip fracture: A three-arm stepped wedge randomized trial | Abstract only |
| Effectiveness of the early use of teriparitide after surgical treatment with intramedullary nails of proximal femoral fragility fractures | Abstract only |
| Effects of a diabetes-specific care model for hip fractured older patients with diabetes: A randomized controlled trial | Inappropriate intervention |
| Effects of a fast track surgery nursing program in perioperative care of older patients with a hip fracture | Inappropriate intervention |
| Effects of a multicomponent exercise program in physical function and muscle mass in sarcopenic/pre-sarcopenic adults | Inappropriate patient population |
| Effects of abaloparatide on major osteoporotic fracture incidence in postmenopausal women with osteoporosis - Results of the Phase 3 ACTIVE trial | Abstract only |
| Effects of abaloparatide on vertebral and non-vertebral fracture incidence in postmenopausal women with osteoporosis-results of the phase 3 active trial | Abstract only |
| Effects of antiresorptive treatment on nonvertebral fracture outcomes | Inappropriate intervention |
| Effects of comprehensive nursing on joint function and psychological rehabilitation of elderly type II diabetes mellitus patients with femoral neck fracture undergoing total hip arthroplasty | Inappropriate intervention |
| Effects of denosumab on bone turnover markers compared to zoledronic acid in severe osteoporotic women: A randomized head to head study | Abstract only |
| Effects of denosumab on fracture risk in japanese patients with osteoporosis-results of 2-year data from the denosumab fracture intervention randomized placebo controlled trial (DIRECT) | Abstract only |
| Effects of Dexmedetomidine on Postoperative Delirium and Expression of IL-1β, IL-6, and TNF-α in Elderly Patients After Hip Fracture Operation | Inappropriate intervention |
| Effects of evidence-based nursing on compliance behavior and quality of life of elderly patients after artificial femoral head replacement | Inappropriate intervention |
| Effects of odanacatib on bmd and overall safety in the treatment of osteoporosis in postmenopausal women previously treated with alendronate | Abstract only |
| Effects of Orem's Self-Care Model on the Life Quality of Elderly Patients with Hip Fractures | Inappropriate intervention |
| Effects of risedronate versus menopausal hormone therapy on bone mineral density in postmenopausal Korean women with hip fracture: a randomized, open-label trial | Abstract only |
| Effects of teriparatide compared with risedronate on recovery after pertrochanteric hip fracture results of a randomized, active-controlled, double-blind clinical trial at 26 weeks | Duplicate |
| Effects of teriparatide on hip and upper limb fractures in patients with osteoporosis: A systematic reviewand meta-analysis | Abstract only |
| Effects of the Cathepsin K inhibitor, ONO-5334, on BMD as measured by 3D QCT in the hip and the spine after 12 months treatment | Abstract only |
| Effects of the cathepsin K inhibitor, ONO-5334, on BMD as measured by 3D QCT in the hip and the spine after 12 months treatment | Abstract only |
| Effects of the cathepsin K inhibitor, ONO-5334, on BMD as measured by 3D QCT in the hip and the spine after 2 years of treatment | Abstract only |
| Effects of the cathepsin k inhibitor, ONO-5334, on BMD as measured by 3d QCT in the hip and the spine after 2years of treatment | Abstract only |
| Efficacy and safety of iron supplementation for the elderly patients undergoing hip or knee surgery: A meta-analysis of randomized controlled trials | Abstract only |
| Efficacy and safety of strontium ranelate in the treatment of male osteoporosis | Abstract only |
| Efficacy and safety of the cathepsin K inhibitor, ONO-5334, and alendronate on post-menopausal osteopenia or osteoporosis: 2-year results from the ocean study | Abstract only |
| Efficacy and safety of two teriparatide formulations: Osteofortil® and forteo® clinical comparison | Abstract only |
| Eighteen months of treatment with abaloparatide followed by six months of treatment with alendronate in postmenopausal women with osteoporosis - Results of the ACTIVExtend trial | Abstract only |
| Ergocalciferol and cholecalciferol induce comparable increases in vitamin d binding protein and free 25-hydroxy-itamin d with no significant change in free 1,25-dihydroxyvitamin d in hip fracture patients | Abstract only |
| Evidence for the treatment of osteoporosis with vitamin d in residential care and in the community dwelling elderly | Inappropriate intervention |
| Exercise for reducing fear of falling in older people living in the community | Inappropriate study design |
| Exercise prescription after fragility fracture in older adults: A scoping review | Inappropriate study design |
| Exercise to Reduce Mobility Disability and Prevent Falls After Fall-Related Leg or Pelvic Fracture: RESTORE Randomized Controlled Trial | Inappropriate patient population |
| Extended venous thromboembolism (VTE) prophylaxis after hip fracture surgery with the ultra-low-molecular-weight heparin (ULMWH) semuloparin | Abstract only |
| Fascia iliaca compartment block as a preoperative analgesic in elderly patients with hip fractures - effects on cognition | Inappropriate intervention |
| Feasibility of C-mill gait-adaptability training in older adults after fall-related hip fracture: User's perspective and training content | Abstract only |
| Final results from a population-based post-fracture intervention based upon administrative health data: A randomized controlled trial | Abstract only |
| FOCUS cognitive ancillary study: Randomized clinical trial of blood transfusion thresholds on delirium severity | Inappropriate intervention |
| FOCUS study: Transfusion trigger in patients with cardiovascular disease undergoing hip fracture surgery | Inappropriate intervention |
| FOCUS: Transfusion triggers after fractured hip repair | Inappropriate intervention |
| Frame study: the foundation effect of rebuilding bone with one year of romosozumab leads to continued lower fracture risk after transition to denosumab | Abstract only |
| FRAME Study: The Foundation effect of rebuilding bone with one year of romosozumab leads to continued lower fracture risk after transition to denosumab | Abstract only |
| Group exercise improves self-efficacy, balance and adl in older people with stroke or hip fracture: A randomised controlled study | Abstract only |
| High versus low dose vitamin D in acute hip fracture patients: A randomised, controlled trial | Abstract only |
| Higher response with bone mineral density (BMD) increase and bone turnover reduction following treatment with monthly injectable ibandronate (IBN) for patients (PTS) with osteoporosis in the mover study | Abstract only |
| Hip fracture accelerated surgical treatment and care track (HIP ATTACK) trial-feasibility pilot | Abstract only |
| Home visits by occupational therapists improve adherence to recommendations: Process evaluation of a randomised controlled trial | Duplicate |
| Home-Based Physical Therapy for Older Adults after Hip Fracture | Inappropriate study design |
| Home-based physiotherapy in patients following hip fracture surgery: A systematic review and meta-analysis of randomized controlled trials | Abstract only |
| Improving adherence with the use of hip protectors among older people living in nursing care facilities: A cluster randomized trial | Inappropriate intervention |
| Improving mobilityand reducing disability in older people through early high-dose vitamin D replacement following hip fracture (the revitahip trial): Preliminary results | Abstract only |
| Improving pain control following fractures in the emergency department: The PAINFREE initiative | Abstract only |
| In vivo vitamin D synthesis vs. medical supplementation in prevention of fractures in osteoporotic patents | Abstract only |
| Incontinence and nocturia in older adults after hip fracture: Analysis of a secondary outcome for a parallel group, randomized controlled trial | Duplicate |
| Indirect comparison meta-analysis of two enoxaparin regimens in patients undergoing major orthopaedic surgery: Impact on the interpretation of thromboprophylactic effects of new anticoagulant drugs | Inappropriate study design |
| Indirect comparison of teriparatide, denosumab, and oral bisphosphonates for the prevention of vertebral and nonvertebral fractures in postmenopausal women with osteoporosis | Inappropriate intervention |
| Individualized home-based exercise programs for older people to reduce falls and improve physical performance: A systematic review and meta-analysis | Inappropriate patient population |
| Indomethacin prophylaxis for heterotopic ossification after acetabular fracture surgery increases the risk for nonunion of the posterior wall | Inappropriate patient population |
| Indomethacin prophylaxis for heterotopic ossification after acetabular fracture surgery increases the risk for non-union of the posterior wall | Duplicate |
| Intensive glycemic control and fracture risk | Abstract only |
| Interdisciplinary intervention decreases cognitive impairment for older taiwanese individuals with hip fracture: Two-year follow-up | Inappropriate intervention |
| Intermittent versus indwelling urinary catheterisation in hip surgery patients: A randomised controlled trial with cost-effectiveness analysis | Inappropriate patient population |
| Long-term intensive family rehabilitation training for postoperative functional recovery in elderly hip fracture patients | Non-English |
| Management of fragility fractures: Impact of the optimus initiative on family physicians | Abstract only |
| Method of osteoporosis awareness practiced in post hip fracture patients | Abstract only |
| Methylprednisolone and inflammatory stress response in older people undergoing surgery for hip fracture: a secondary analysis of a randomized controlled trial | Inappropriate intervention |
| Mixed treatment comparison of prophylaxis regimens for the prevention of venous thromboembolism in total hip replacement, HIP fracture, total knee replacement and general surgery | Abstract only |
| Monthly cycles of teriparatide and raloxifene increase bmd comparable to continuous teriparatide: Is it possible to “open the anabolic window?” | Abstract only |
| Musculoskeletal rehabilitation after hip fracture: A review | Inappropriate study design |
| Myostatin as a potential biomarker to monitor sarcopenia in hip fracture patients undergoing a multidisciplinary rehabilitation and nutritional treatment: a preliminary study | Duplicate |
| Nalbuphine for analgesia after fracture surgery and its effect on circulating inflammatory factors | Inappropriate intervention |
| Nasal salmon calcitonin prevents bone microstructure alterations in early postmenopausal women | Inappropriate patient population |
| New horizons for zoledronic acid: Results of the HORIZON trials in postmenopausal women with osteoporosis and after hip fracture | Inappropriate study design |
| Nutritional assessment of older adults with hip fracture: A systematic review | Abstract only |
| Odanacatib anti-fracture efficacy and safety in postmenopausal women with osteoporosis: Results from the phase III long-term odanacatib fracture trial | Abstract only |
| Odanacatib anti-fracture efficacy and safety in postmenopausal women with osteoporosis: Results from the phase III long-term odanacatib fracture trial | Abstract only |
| Once-monthly oral ibandronate in postmenopausal osteoporosis: Translation and updated review | Inappropriate intervention |
| Once-yearly treatment with zoledronic acid continues to be effective in old age | Abstract only |
| One year of romosozumab followed by two years of denosumab maintains fracture risk reduction: Analysis for the Japanese population of phase 3 FRAME extension | Abstract only |
| Oral bisphosphonate use after hip fracture is associated with reduced mortality | Abstract only |
| Oral nutritional supplement (ONS) improved nutritional status in malnourished patients receiving hip fracture surgery | Abstract only |
| Oral rivaroxaban for the prevention of symptomatic venous thromboembolism after elective hip and knee replacement: The RECORD study programme | Abstract only |
| Ortho-geriatric models and optimal outcomes: A systematic review and meta-analysis | Abstract only |
| Otto Aufranc Award: A Multicenter, Randomized Study of Outpatient versus Inpatient Total Hip Arthroplasty | Inappropriate patient population |
| Outcomes of activities of daily living, cognition and mobility in the Trondheim Hip Fracture Trial. A randomized controlled trial | Abstract only |
| Outcomes of hip fractures: Rehabilitation programmes: Comprehensive Geriatric Assessment and Rehabilitation-a prerequisite for successful treatment of people who have suffered a hip-fracture | Abstract only |
| Overlapping and follow-up of alendronate to teriparatide results in continuing volumetric bone mass increase measured by quantitative computed tomography | Abstract only |
| Overlapping and follow-up of Alendronate to Teriparatide treatment results in maintenance of excess BMD gain | Abstract only |
| Overlapping and follow-up of alendronate to teriparatide treatment results in maintenance of excess BMD gain | Abstract only |
| Overlapping and follow-up of alendronate to teriparatide treatment results in maintenance of excess BMD gain | Abstract only |
| Overlapping and follow-up of alendronate to teriparatide treatment results in maintenance of excess BMD gain | Abstract only |
| Overlapping and follow-up of alendronate to teriparatide treatment results in maintenance of excess BMD gain | Abstract only |
| Overlapping and follow-up of alendronate to teriparatide treatment results in maintenance of excess BMD gain | Abstract only |
| Pain treatment in post-traumatic hip fracture in the elderly: Regional block vs. systemic non-steroidal analgesics | Inappropriate intervention |
| Parecoxib, propacetamol, and their combination for analgesia after total hip arthroplasty: a randomized non-inferiority trial | Inappropriate intervention |
| Patient blood management in elective total hip- And knee-replacement surgery (Part 1): A randomized controlled trial on erythropoietin and blood salvage as transfusion alternatives using a restrictive transfusion policy in erythropoietin-eligible patients | Inappropriate intervention |
| People living in nursing care facilities who are ambulant and fracture their hips: description of usual care and an alternative rehabilitation pathway | Inappropriate study design |
| Peri-operative enhanced recovery hip fracture care of patients with dementia (perfected): Cluster randomised control trial | Abstract only |
| PERI-OPERATIVE ENHANCED RECOVERY HIP FRACTURE CARE OF PATIENTS WITH DEMENTIA (PERFECTED): RCT RESULTS | Abstract only |
| Persistent effect of zoledronic acid in reducing the risk for fractures: Pooled analysis of horizon-PFT and RFT | Abstract only |
| Phase 3 fracture trial of odanacatib for osteoporosis-baseline characteristics and study design | Abstract only |
| Physician notification from administrative health data is a cost-effective post-fracture intervention: Interim report from a randomized controlled trial | Abstract only |
| Physiotherapy interventions for people with dementia and a hip fracture—a scoping review of the literature | Inappropriate study design |
| Platelet rich therapies for long bone healing in adults. | Inappropriate intervention |
| Pooled analysis of two randomized studies comparing fondaparinux 2.5 mg and enoxaparin 40 mg for preventing venous thromboembolism (VTE) in hip surgery using new study endpoints | Abstract only |
| Post-Acute Care for Older People Following Injury: A Randomized Controlled Trial | Inappropriate patient population |
| Post-acute physiotherapy for primary total hip arthroplasty: A cochrane systematic review | Inappropriate patient population |
| Post-alendronate DXA monitoring strategy based on 5-year changes in BMD | Abstract only |
| Post-fracture management of patients with hip fracture: A perspective | Inappropriate study design |
| Postoperative blood transfusion strategy in frail, anemic elderly patients with hip fracture | Inappropriate intervention |
| Postoperative comprehensive nursing care improved the prognosis and life quality of patients with minimally invasive retrograde intramedullary nail treatment for femur supracondylar fracture | Inappropriate patient population |
| Postoperative management of hip fractures: Interventions associated with improved outcomes | Inappropriate study design |
| Postoperative subdural analgesia with tramadol vs morphine in intertrocanteric fracture of hip | Abstract only |
| Preventing nonvertebral osteoporotic fractures with extended-interval bisphosphonates: Regimen selection and clinical application | Inappropriate intervention |
| Progressive resistance training reduces disability and improves physical performance in elderly people after surgical repair of hip fracture | Abstract only |
| Prolonged versus standard-duration venous thromboprophylaxis in major orthopedic surgery a systematic review | Inappropriate study design |
| Randomized controlled trial to assess the safety and efficacy of odanac atib in the treatment of men with osteoporosis | Abstract only |
| Randomized controlled trial to assess the safety and efficacy of odanacatib in the treatment of men with osteoporosis | Abstract only |
| Randomized trial of alendronate plus vitamin D(3) versus standard care in osteoporotic postmenopausal women with vitamin D insufficiency | Inappropriate patient population |
| Rapid onset and sustained efficacy (ROSE) study of zoledronic acid vs alendronate in postmenopausal women with osteoporosis: Quality of life (QOL), compliance and therapy preference | Abstract only |
| Reducing delirium in elderly patients with hip fracture: A multi-factorial intervention study | Inappropriate intervention |
| Reduction in the risk of clinical fractures after a single dose of zoledronic acid 5 mg | Abstract only |
| Reduction in the risk of clinical fractures after a single dose of zoledronic acid 5 milligrams | Inappropriate patient population |
| Reduction in the risk of clinical fractures after a single dose of zoledronic acid 5mg | Abstract only |
| Rehabilitation After Hip Fracture for Nursing Home Residents: A Controlled Feasibility Trial | Inappropriate study design |
| Rehabilitation of two-part fractures of the neck of the humerus (two-year follow-up) | Inappropriate patient population |
| Resolution of effects on bone turnover markers and BMD after discontinuation of long-term bisphosphonate use | Abstract only |
| Resolution of effects on bone turnover markers and bone mineral density after discontinuation of long-erm bisphosphonate use | Abstract only |
| Resolution of effects on bone turnover markers and bone mineral density after discontinuation of long-term bisphosphonate use | Abstract only |
| Responder analysis of the effects of abaloparatide (parathyroid hormone related peptide) and teriparatide on bone mineral density in postmenopausal women with osteoporosis: Results of the active trial | Abstract only |
| Response rates for hip, femoral neck and lumbar spine BMD are higher for patients treated with abaloparatide when compared to placebo or teriparatide - Results of the ACTIVE trial | Abstract only |
| Results of 2-year data from denosumab fracture intervention randomized placebo controlled trial (direct) | Abstract only |
| Results of the oracal trial: A phase 3 randomized trial of the safety and efficacy of orally administered recombinant salmon calcitonin tablets in postmenopausal women with osteoporosis | Abstract only |
| Risk factors for developing vertebral fractures after vertebroplasty | Abstract only |
| Role of acute negative pressure wound therapy over primarily closed surgical incisions in acetabular fracture ORIF: A prospective randomized trial | Inappropriate patient population |
| Role of dietary protein and exercise on tryptophan-kynurenine metabolism in older patients during muscle disuse | Abstract only |
| Role of nutritional supplementation in elderly patients with hip fractures | Inappropriate study design |
| Romosozumab improves lumbar spine bone mineral density and bone strength greater than alendronate as assessed by quantitative computed tomography and finite element analysis in the ARCH trial | Abstract only |
| Safety and efficacy of intravenous acetaminophen in the elderly after major orthopedic surgery: Subset data analysis from 3, randomized, placebo-controlled trials | Inappropriate intervention |
| Safety and tolerability of monthly I.V. Ibandronate injections: MOVER study 3-year analysis | Abstract only |
| Safety and tolerability of odanacatib therapy in postmenopausal women with osteoporosis: Results from the phase III long-term odanacatib fracture trial | Abstract only |
| Safetyand tolerability of monthly I.V. ibandronate injections: Mover study 3-year analysis | Abstract only |
| Semuloparin for prevention of venous thromboembolism after major orthopedic surgery: Results from three randomized clinical trials, SAVE-HIP1, SAVE-HIP2 and SAVE-KNEE | Inappropriate patient population |
| Severe prevalent vertebral fractures predict subsequent vertebral and non-vertebral fractures: A 3-year prospective study | Abstract only |
| Short-term effects of teriparatide versus placebo on bone biomarkers, structure, and fracture healing in women with lower-extremity stress fractures: A pilot study | Inappropriate patient population |
| Short-term functional recovery between immediate- and delayed bisphosphonate treatment in patients with femoral neck fractures: A randomized controlled trial | Abstract only |
| Short-term oral nutritional supplements and nutrition intervention in elderly patients after hip fracture surgery: A randomized controlled clinical trial | Abstract only |
| Single annual injectable treatment for postmenopausal osteoporosis | Inappropriate intervention |
| Subcutaneous methylnaltrexone for treatment of acute opioid-induced constipation: Phase 2 study in rehabilitation after orthopedic surgery | Inappropriate patient population |
| Subtrochanteric fractures: Results from the HORIZON-Recurrent fracture trial | Abstract only |
| Superior gains in bone mineral density and estimated strength at the hip for romosozumab compared with teriparatide in women with postmenopausal osteoporosis transitioning from bisphosphonate therapy: Results of the phase 3 open-label structure study | Abstract only |
| Systematic review and meta-analysis of the efficacy and safety of alendronate and zoledronate for the treatment of postmenopausal osteoporosis | Inappropriate intervention |
| Systematic review of raloxifene in postmenopausal Japanese women with osteoporosis or low bone mass (osteopenia) | Inappropriate intervention |
| Systematic review on interventions to improve osteoporosis investigation and treatment in fragility fracture patients | Inappropriate study design |
| Systematic review on interventions to improve osteoporosis investigation and treatment in fragility fracture patients | Abstract only |
| Ten years of denosumab treatment in postmenopausal women with osteoporosis: Results from the FREEDOM extension trial | Abstract only |
| Teriparatide accelerates proximal humerus fracture consolidation-the terafrap study | Abstract only |
| Teriparatide and antiresorptive combination treatment subsequent to 9 months of teriparatide monotherapy | Inappropriate patient population |
| Teriparatide and antiresorptive combination treatment subsequent to 9 months of teriparatide monotherapy | Abstract only |
| Teriparatide and antiresorptive combination treatment subsequent to 9 months of teriparatide monotherapy | Abstract only |
| Teriparatide and the risk of nonvertebral fractures in women with postmenopausal osteoporosis | Inappropriate intervention |
| Teriparatide compared with risedronate and the risk of clinical vertebral fractures: 2-year results of a randomized, double-dummy clinical trial | Inappropriate patient population |
| Teriparatide seems to improve recovery after pertrochanteric hip fracture: Comparison with risedronate in a randomized, controlled trial | Abstract only |
| The combined administration of systemic and topical tranexamic acid for total hip arthroplasty: Is it better than systemic? | Inappropriate patient population |
| The economic consequences of hip fractures: Impact of home exercise and high-dose vitamin D | Abstract only |
| The Effect of 1 Year of Romosozumab on the Incidence of Clinical Vertebral Fractures in Postmenopausal Women With Osteoporosis: Results From the FRAME Study | Inappropriate patient population |
| The effect of 6 versus 9 years of zoledronic acid treatment in osteoporosis: A randomized extension to the horizon-pivotal fracture trial (PFT) | Abstract only |
| The effect of 6 versus 9 years of zoledronic acid treatment in osteoporosis: A randomized second extension to the HORIZON-pivotal fracture trial (PFT) | Inappropriate intervention |
| The effect of a single early high-dose Vitamin D supplement on fracture union in patients with hypovitaminosis D: A prospective randomised trial | Inappropriate patient population |
| The effect of bone-active medication use on bone mineral density post-hip fracture | Abstract only |
| The effect of calcium B-hydroxy-B-methylbutyrate, vitamin D and protein supplementation on postoperative immobilization in elderly malnourished patients with hip fracture: A randomized controlled study | Abstract only |
| The effect of corticosteroids on the prevention of fat embolism syndrome after long bone fracture of the lower limbs: A systematic review and meta-analysis | Non-English |
| The effect of denosumab on bone mineral density (BMD) assessed by baseline bone turnover in men with low BMD | Abstract only |
| The effect of intravenous iron on erythropoiesis in older people with hip fracture | Inappropriate intervention |
| The effect of isolytic contraction and passive manual stretching on pain and knee range of motion after hip surgery: A prospective, double-blinded, randomized study | Inappropriate patient population |
| The effect of monthly I.V. Ibandronate injections on Japanese patients with high-risk primary osteoporosis: Subgroup analysis of the phase III mover study | Abstract only |
| The effect of skin traction on preoperative pain and need for analgesics in patients with intertrochanteric fractures: A randomized clinical trial | Inappropriate intervention |
| The effects of muscle strength and power training on mobility among older hip fracture patients | Duplicate |
| The effects of oral bisphosphonate therapy on the peripheral skeleton in postmenopausal osteoporosis: The trio study | Abstract only |
| The efficacy of rivaroxaban combined with aspirin on prevention of deep venous thrombosis in elderly patients with hip fracture | Abstract only |
| The importance of the orthopaedic surgeon in the prevention of a second fragility hip fracture | Abstract only |
| The influence of inpatient comprehensive geriatric care on elderly patients with hip fractures: A meta-analysis of randomized controlled trials | Inappropriate study design |
| The Normobaric oxygen paradox (NOP): A safe alternative to postoperative transfusions after hip surgery? | Abstract only |
| The orthogeriatric comanagement improves clinical outcomes compared with consultant geriatric service and traditional model | Abstract only |
| The placebo-controlled fracture study in postmenopausal women with osteoporosis: The foundation effect of rebuilding bone with one year of romosozumab leads to continued lower fracture risk after transition to denosumab | Abstract only |
| The TOronto ThromboProphylaxis Patient Safety Initiative (TOPPS): A cluster randomised trial | Inappropriate patient population |
| The Trondheim Hip-Fracture Trial - A cost effectiveness analysis of comprehensive geriatric care in hip-fracture patients | Abstract only |
| The ultra-low-molecular-weight heparin (ULMWH) semuloparin for prevention of venous thromboembolism (VTE) after hip fracture surgery | Abstract only |
| Therapeutic exercises for proximal femoral fracture of super-aged patients: Effect of walking assistance using body weight-supported treadmill training (BWSTT) | Abstract only |
| Tight calorie control (TICACOS) in geriatric hip fracture patients | Abstract only |
| Tight calorie control in geriatric hip fracture patients: Preliminary results of geriatric ticacos study | Abstract only |
| Time to onset of anti-fracture efficacy and persistence of effect of zoledronic acid 5 mg in women with osteoporosis or recent hip fracture | Abstract only |
| Time to onset of anti-fracture efficacy and persistence of effect of zoledronic acid 5 mg in women with osteoporosis or recent hip fracture | Abstract only |
| Timing of rehabilitation on length of stay and cost in patients with hip or knee joint arthroplasty: A systematic review with meta-analysis | Inappropriate study design |
| Timing of the initiation of bisphosphonates after surgery for fracture healing: a systematic review and meta-analysis of randomized controlled trials | Inappropriate study design |
| Topical application of tranexamic acid in primary total hip arthroplasty: A systemic review and meta-analysis | Inappropriate intervention |
| Traditional Chinese and western medicine for the prevention of deep venous thrombosis after lower extremity orthopedic surgery: a meta-analysis of randomized controlled trials | Inappropriate study design |
| Transfusion trigger trial for functional outcomes in cardiovascular patients undergoing surgical hip fracture repair (FOCUS): The principal results | Inappropriate intervention |
| Treatment of fear of falling in geriatric rehabilitation after hip fracture: Effects of a cluster randomized controlled trial (FIT-HIP) | Abstract only |
| Treatment with PTH 1-84 in male patients with severe osteoporosis - Results from a prospective 24 month open-label trial | Abstract only |
| Treatment with zoledronate subsequent to denosumab in osteoporosis: a randomized trial | Inappropriate intervention |
| Ultrasound-guided suprainguinal fascia iliaca compartment block versus periarticular infiltration for pain management after total hip arthroplasty: A randomized controlled trial | Inappropriate intervention |
| Use of alendronate for prevention of endoprostheses instability in osteoporosis | Abstract only |
| Use of fondaparinux in current clinical practice for thromboprophylaxis following major orthopedic surgery in France: The aristote study | Abstract only |
| Using a sequential explanatory mixed method to evaluate the therapeutic window of opportunity for initiating osteoporosis treatment following fragility fractures | Inappropriate patient population |
| Value of rapid rehabilitation nursing in patients with hip fracture and its influence on patients’ pain | No access to full article |
| Venous Thromboembolism Prophylaxis After Major Orthopaedic Surgery: A Pooled Analysis of Randomized Controlled Trials | Inappropriate study design |
| Vertebral fracture efficacy during risedronate therapy in patients using proton pump inhibitors | Inappropriate patient population |
| Vitamin D, at high doses, prevents fractures | Inappropriate intervention |
| Vitamin D, calcium, OR combined supplementation for the primary prevention of fractures in community-dwelling adults evidence report and systematic review for the US Preventive Services Task Force | Inappropriate intervention |
| VK5211, a novel selective androgen receptor modulator (SARM), significantly improves lean body mass in hip fracture patients: Results of a 12 week phase 2 trial | Abstract only |
| Weekly alendronate plus vitamin D(3) 5,600 IU vs. Standard care: Effect on serum 25(OH) vitamin D, bone turnover markers, and BMD in osteoporotic postmenopausal women with vitamin D inadequacy-1 year results of a randomized trial | Abstract only |
| Weekly alendronate plus vitamin D(3) 5600 IU vs. standard care: Effect on serum 25(OH) vitamin D, bone turnover markers, and BMD in osteoporotic postmenopausal women with vitamin D inadequacy-1-year results of a randomized trial | Abstract only |
| Weekly alendronate plus vitamin D3 5600IU vs. standard care: Effect on serum 25(OH) vitamin D, bone turnover markers, and BMD in osteoporotic postmenopausal women with vitamin D inadequacy - 1-Year results of a randomized trial | Abstract only |
| Which patients benefit from orthogeriatric treatment? Results from the Trondheim Hip Fracture Trial | Abstract only |
| Zoledronic acid improves health-related quality of life in patients with hip fracture: Results of HORIZON-RFT | Abstract only |
| Zoledronic acid improves health-related quality of life in patients with hip fracture: Results of HORIZON-RFT | Abstract only |
| Zoledronic acid improves Health-related quality of life in patients with hip fracture: Results of HORIZON-RFT | Abstract only |
| Zoledronic acid in preventing fractures in women with postmenopausal osteoporosis: A meta analysis | Inappropriate intervention |
| Zoledronic acid in the management of osteoporosis: The HORIZON trials | Inappropriate intervention |
| Zoledronic acid reduces recurrent clinical fracture in patients with hip fracture | Duplicate |
| Zoledronic acid reduces the increased risk conferred by further fractures | Abstract only |
| Zoledronic acid reduces the increased risk conferred by further fractures | Abstract only |
| Zoledronic acid substantially reduces the risk of morphometric vertebral and clinical fractures | Abstract only |
| Thromboembolic prophylaxis in hip fracture | Jadad score < 3 |
| Heparin versus danaproid for prevention of venous thromboembolism after hip surgery | Inappropriate study design |
| Nutritional care: the effectiveness of actively involving older patients | Inappropriate study design |
| Effect of Calcium Œ≤-Hydroxy-Œ≤-Methylbutyrate (CaHMB), Vitamin D, and Protein Supplementation on Postoperative Immobilization in Malnourished Older Adult Patients With Hip Fracture: a Randomized Controlled Study | Inappropriate study design |
| Breaking the cycle of recurrent fracture: implementing the first fracture liaison service (FLS) in British Columbia, Canada | Inappropriate study design |
| Anticoagulant prophylaxis, thromboembolism and mortality in elderly patients with hip fractures. A controlled clinical trial | No access to full article |
| Clinical study on effect of Osteoking in preventing postoperational deep venous thrombosis in patients with intertrochanteric fracture | Jadad score < 3 |
| Thromboembolic prophylaxis in orthopaedic trauma patients: a comparison between a fixed dose and an individually adjusted dose of a low molecular weight heparin (nadroparin calcium) | Inappropriate patient population |
| Rehabilitation Care for Hip Fracture | No results |
| HIP Fracture REhabilitation Programme | No results |
| Improving Community Ambulation After Hip Fracture | Duplicate |
| Hip Protector for Prevention of Hip Fracture | No results |
| Three Care Models for Elderly Patients With Hip Fracture | Inappropriate intervention |
| Treatment Efficacy of Leg Cycling as Part of Physiotherapy Treatment in Elderly Patients With Hip Fracture | No results |
| Intervention Program for Elderly Patients With Hip Fracture | Inappropriate intervention |
| Vibration Therapy as an Intervention for Enhancing Trochanteric Hip Fracture Healing in Elderly Patients | No results |
| Effects of Bisphosphonates and Nutritional Supplementation After a Hip Fracture | Duplicate |
| Training of Patients With Hip Fracture | No results |
| Randomized Controlled Trial for Exparel Hip Fracture | Inappropriate intervention |
| Effect of Leg Strengthening Exercise After Hip Fracture | No results |
| Acute Hip Fracture Study in Patients 65 Years or Greater | No results |
| High Intensity Physiotherapy for Hip Fractures | Duplicate |
| Pain Management in Geriatric Hip Fracture | No results |
| Physiotherapy, Nutritional Supplement and Anabolic Steroids in Rehabilitation of Patients With Hip Fracture. | Duplicate |
| A Comparison of Two Pain Control Techniques on Deliruim in Hip Fracture Patients | No results |
| Postoperative Blood Transfusion for Frail Elderly With Hip Fracture | Inappropriate intervention |
| Randomised Trial Comparing Iron Supplementation Versus Placebo in the Treatment of Anaemia After Hip Fracture | Duplicate |
| A Randomised, Controlled Comparison of Vitamin D Strategies is Acute Hip Fracture Patients | Duplicate |
| Study of Low-Magnitude, High-Frequency Vibration Treatment on Osteoporotic Hip Fracture Healing | No results |
| Nutritional Intervention for Geriatric Hip Fracture Patients | Duplicate |
| Use of Mobile App to Enhance Geriatric Hip Fracture Rehabilitation | No results |
| Postoperative Intervention Program Effectiveness in Hip Fracture Patients: A Randomized Clinical Trial | No results |
| Post-acute Care for Patients With Hip Fracture | No results |
| Improving Functional Recovery After Hip Fracture | No results |
| IV Iron in Association With Tranexamic Acid for Hip Fracture | Inappropriate intervention |
| Prevention of Delirium Among Elderly Patients With Hip Fractures | No results |
| The Effect of Supplementation of Vitamin D Deficiency in Older People With Acute Hip Fracture: | No results |
| The Stronger at Home Study | No results |
| Care Pathway for Sub-acute Hip Rehabilitation | Duplicate |
| Better Hips- Better Function | No results |
| Functional Later Rehabilitation in Older Adults: Effectiveness of Physical Exercises | Duplicate |
| Innovative Models in the Rehabilitation of the Elderly With Hip Fractures Through Technological Innovation | Duplicate |
| Impact of a Nutritional Supplement on the Recovery of the Nutritional Status of Patients With Spontaneous Hip Fracture | No results |
| Does Early Ambulation After Hip Fracture Surgery Accelerate Recovery? | No results |
| Effects of Long-term Intensive Home-based Physiotherapy on Older People With an Operated Hip Fracture or Frailty (RCT). | No results |
| Effects of Tranexamic Acid on Blood Loss and Transfusion Requirement Following Hip Fracture | Inappropriate patient population |
| Effect of a Medical Food Supplement in Hospitalized Patients Recovering From Surgery | No results |
| BHS5 - Testing the Effectiveness of the Exercise Plus Program | No results |
| Hyperprotein Nutritional Intervention in Elderly Patients With Hip Fracture and Sarcopenia | Duplicate |
| Study of Efficacy and Safety of Bimagrumab in Patients After Hip Fracture Surgery | No access to full article |
| The Outcome and Cost Analysis of Home-care Physical Therapy for Postoperative Hip Fracture Patients | No results |
| Abaloparatide and Pelvic Fracture Healing | No results |
| Testosterone Therapy After Hip Fracture in Elderly Women | No results |
| Transcutaneous Nerve Stimulation for Post-operative Acute and Chronic Pain | Inappropriate patient population |
| Postop Pain Control in Hip Fracture Surgeyr: Fascia Iliaca Compartment Block Versus Fracture Block | Inappropriate intervention |
| Transcutaneous Electrical Nerve Stimulation on Pain Intensity of Patients With Hip Arthroplasty | No results |
| Fragility Fracture Integrated Rehabilitation Management (FIRM) | No results |
| Evaluating the Ability of Zoledronic Acid to Reduce the Rate of Subsequent Osteoporotic Fractures After a Hip Fracture | Duplicate |
| RCT: Added Value of Coordinator for the Management of Hip Fracture Patients | No results |
| Trial of Osteoporosis Intervention Strategies in Hip Fracture Patients | No results |
| A Study That Will Compare the Effect of Two Drugs on Participants With Low Bone Mass and a Recent Hip Fracture | No access to full article |
| The Effects of a Home-based Occupational Therapy Tele-rehabilitation for Outpatients After Hip Fracture Surgery | No results |
| Effects of Exercise on Markers of Inflammation in Skeletal Muscle in Elderly Hip Fracture Patients | No results |
| Observation and Progressive Strength Training After Hip Fracture | No results |
| Post-operative Haloperidol Versus Placebo for Prevention of Post-operative Delirium After Acute Hip Surgery | No results |
| Starting a Testosterone and Exercise Program After Hip Injury | No results |
| Efficacy of a Post-Rehabilitation Exercise Intervention | Duplicate |
| Hip Fracture Study of GSK576428 (Fondaparinux Sodium) | Inappropriate study design |
| Crutch Use After Arthroscopic Hip Surgery | No results |
| A Care Model for Elderly Hip-fractured Persons With Cognitive Impairment and Their Family Caregivers | No results |
| Do Mobility Technicians Provide Benefit to Patients Recovering From Hip or Lower Extremity Long Bone Fracture Surgery? | No results |
| The Study of the Early Administration of Alendronate on Prevention of Bone Loss After Hip Fracture. | No results |
| Nocturnal Hypoxia in Geriatric Patients After Hip Fracture | No results |
| Preventing Loss of Weight, Fat Free Mass and Activities of Daily Living | No results |
| Recovery of Physical Functioning After Hip Fracture | No results |
| Secondary Fracture Prevention in Patients Who Suffered From Osteoporotic Fracture | Inappropriate patient population |
| NMES and Pelvic Fracture Rehabilitation | No results |
| A New Clinical Pathway for Patients With Fractured Neck of Femur | Duplicate |
| A Care Model for Hip-fractured Elderly Persons With Diabetes Mellitus | No results |
| Mobilizing Evidence Into Action to Improve Outcomes of Vulnerable Seniors | Duplicate |
| The Influence of Clinical Pharmacist on the Quality of Drug Prescribing and Rehabilitation Outcomes in Post-acute Hip Fractured Patients | Inappropriate study design |
| PTH(1-34) and Pelvic Fracture Healing - a Randomized Controlled Trial | No results |
| Role of Vacuum Assisted Closure (VAC) Device in Postoperative Management of Pelvic and Acetabular Fractures | Inappropriate patient population |
| The Use of NMES With Pelvic Fracture Rehabilitation | No results |
| Effectiveness of Two Types of Treatment in Restoring Muscle After Hip or Knee Surgery | Inappropriate patient population |
| Norwegian Capture the Fracture Initiative | Duplicate |
| Evaluation of Multidisciplinary Recovery After Surgery Program in Orthopedics and Traumatology | No results |
| Effect of Teriparatide on Hip Fracture Healing | No access to full article |
| Second Study of the Effect of Teriparatide on Hip Fracture Healing | No access to full article |
| Evaluation of the Effectiveness of Delirium Preventive Care Protocol | No results |
| Study To Assess FRacTure Healing With SclerosTin Antibody - Hip | No access to full article |
| Maximal Strength Training Following Hip Fracture Surgery: Impact on Muscle Mass, Balance, Walking Efficiency and Bone Density | No results |
| Function Focused Care: Fracture Care at Home | No results |
| Tight Caloric Balance in Geriatric Patients | No results |
| Effectiveness of Home-based Rehabilitation Program | Duplicate |
| To Investigate the Effect of Early Community-care Program on Fracture Hip Patient | No results |
| The Effect of Teriparatide on Bone Union in Unstable Intertrochanteric Fracture Patients Treated With PFNA | No results |
| Addition of Aerobic Training to Conventional Rehabilitation After Femur Fracture | Duplicate |
| Short-term Functional Recovery Between Early- and Late Bisphosphonate Treatment Following Hemiarthroplasty | Duplicate |
